# Routes to Advanced Intermediates in the Synthesis of Tetracarbocyclic Sesquiterpenoids Daphnenoid A and Artatrovirenols A and B

**DOI:** 10.1021/acs.orglett.3c04199

**Published:** 2024-02-19

**Authors:** Jiarui Zong, Kirsten E. Christensen, Jeremy Robertson

**Affiliations:** Department of Chemistry, University of Oxford, Chemistry Research Laboratory, Mansfield Road, Oxford OX1 3TA, United Kingdom

## Abstract

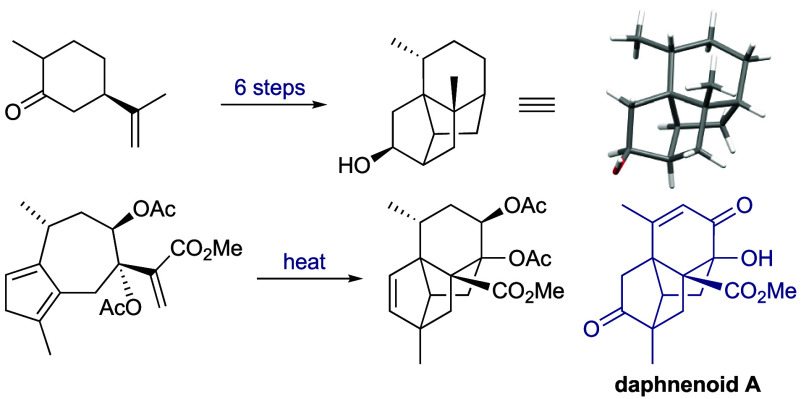

A short route from dihydrocarvone is described, which led to the tetracarbocyclic core common to artatrovirenol A and B and daphnenoid A. A variant of this route afforded guaia-4,6-dien-3-one (from *Enterospermum madagascarensis*) and its epimer. From 2-(2-oxoethyl)furan, a 15-step sequence then delivered the complete carbon skeleton and all functionality necessary for daphnenoid A. Key steps in the route include diastereoselective intramolecular oxidopyrylium cycloaddition, oxa-bridge cleavage under “push–pull” conditions, and intramolecular Diels–Alder cycloaddition.

The sesquiterpenes and their oxidized derivatives, the sesquiterpenoids, form a major class of natural products with structures based on 1.5 (*sesqui-*) C_10_-monoterpene units, the first established being those of α-santalol^1^ and farnesol.^2^ Their wide-ranging biological properties and vast range of structural types have attracted the efforts of synthetic chemists for more than a century, with many of these efforts patterned on biosynthetic speculations.^3^ Recently, a new caged-ring sesquiterpenoid subclass has emerged in two papers reporting the structures (Figure 1) of artatrovirenols A **1** and B **2** from *Artemisia atrovirens*,^4^ and daphnenoid A **3** from *Daphne penicillata*.^5^ The same tetracyclic ring system is also found in the prenylated phloroglucinol garcinielliptone enantiomers HG and HH from *Garcinia subelliptica*.^6^

**Figure 1:**
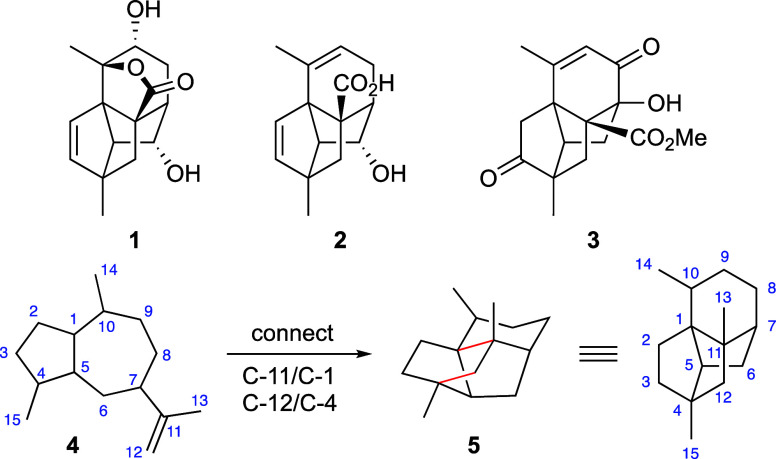
Structures of artatrovirenols A **1** and B **2**, daphnenoid A **3**, and the (biosynthetic) relationship between their core framework **5** and the guaiane framework **4**.^9^

Tetracarbocyclic frameworks are rare in sesquiterpenoids, those containing cyclopropanes excepted; therefore, this new sesquiterpene subclass is of interest not least from a biosynthetic standpoint. Both Chen’s^4^ and Song’s^5^ reports associate these novel structures with the guaiane system **4**. The new framework **5** can be derived from **4** both conceptually and biosynthetically by connecting the isopropenyl Δ^11,12^ carbons to C-1 and C-4, respectively. Building on our recent studies of simple guaianes,^7^ our group set out to explore the synthesis of daphnenoid A based on an intramolecular Diels–Alder (IMDA) cycloaddition or an equivalent process from an appropriate guaianoid precursor. Very recently, Zhu’s group reported the total synthesis of (−)-artatrovirenol A by an unrelated synthetic strategy,^8^ which has prompted us to disclose our current progress in this area.

Retrosynthetically, it was envisaged that the new ring system would be constructed from enone **7** (Scheme 1) by using a silylation-induced (Mukaiyama) Michael/Michael-type reaction via dienol ether **6**. In this scheme, the appropriately configured protected 3°-alcohol in this enone would arise by elimination of the 1,7-oxa bridge in **8**, the product of intramolecular oxidopyrylium cycloaddition via **9**. Completing the analysis led back to a 2,5-disubstituted furan of general form **10**, although it was appreciated that the combination of a furfurylic alcohol, a β,γ-unsaturated ketone, and a methylene flanked by both electron releasing (furan) and electron withdrawing (carbonyl) functionality would render this specific precursor somewhat fragile.

**Scheme 1:**
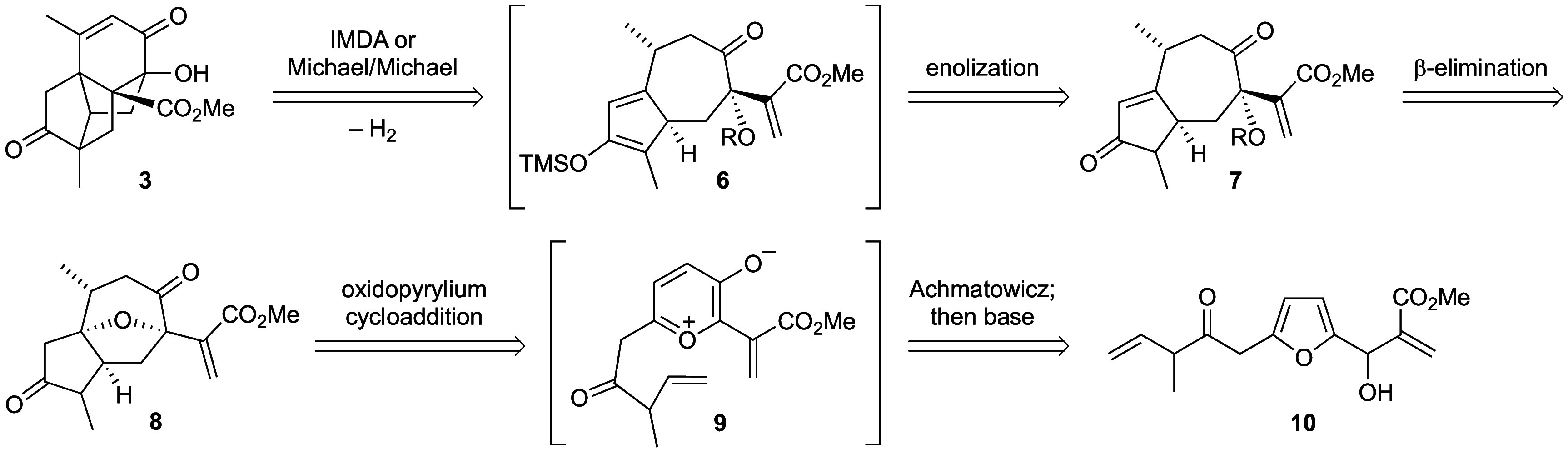
Retrosynthetic Analysis of Daphnenoid A

Before embarking on the route outlined above, a preliminary investigation of the viability of the key formal cycloaddition step (*cf*. **7** → **3**) was undertaken (Scheme 2). The known ring-expansion product **11**(10) of dihydro-(*R*)-carvone was alkylated to give 1,4-diketone **12** after ester hydrolysis and decarboxylation. Intramolecular aldol condensation gave a complex mixture of diastereomeric and regioisomeric enones **13**, but this was considered to be of no consequence because the thermal silylating conditions^11^ for the subsequent step were expected to generate some equilibrium concentration of **14**,^12^ the only intermediate capable of undergoing [4 + 2]-cycloaddition at a reasonable rate. In the event, and despite no dienophilic activation,^13^ the crude reaction product consisted mainly of the desired tetracyclic ketone **15**. This ketone was found to be unstable toward purification by chromatography on silica gel; therefore, ketone reduction was carried out, which enabled a pure sample of alcohol **16** to be obtained whose structure was confirmed by single-crystal X-ray diffraction.^14^

**Scheme 2:**
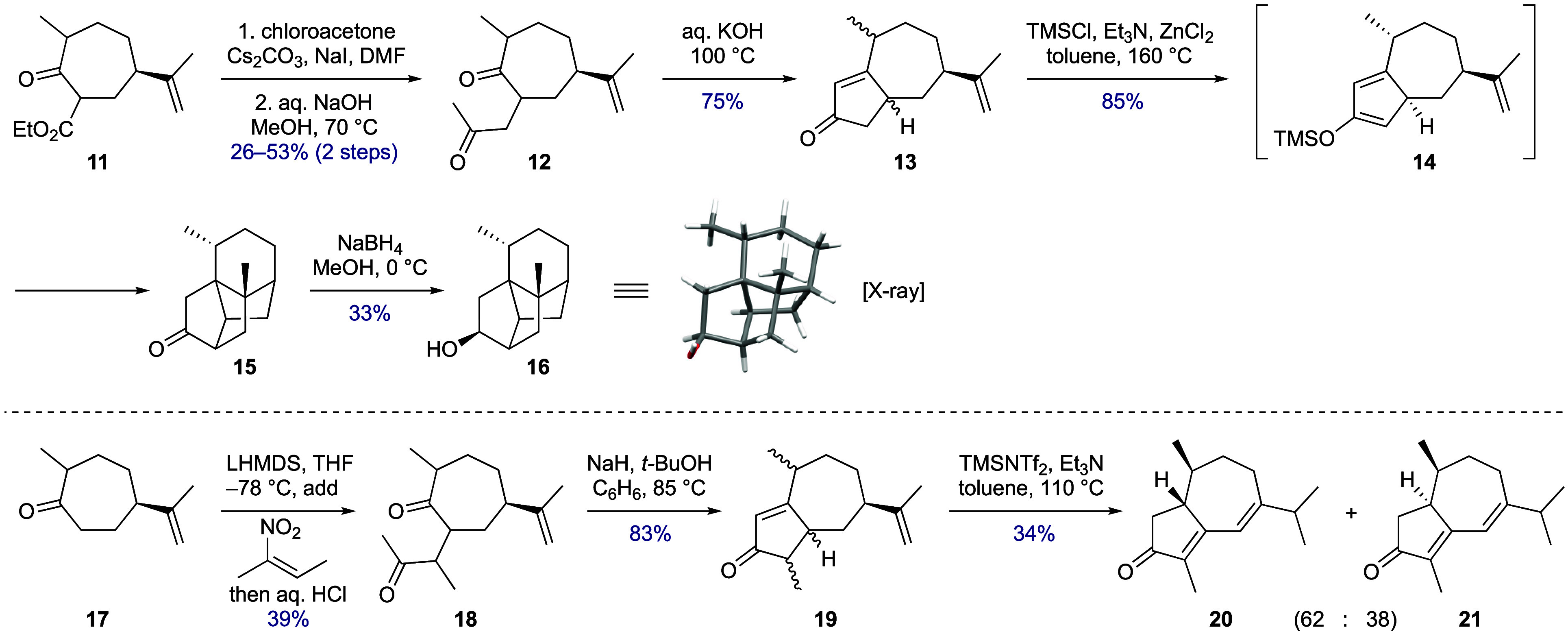
Model Studies of the IMDA-Equivalent Cycloaddition

Adapting this route to incorporate the C-15 methyl group required for the complete sesquiterpene skeleton was not achieved. Although a suitable substrate **19** was prepared from the same keto-ester **11**, attempts to access the [4 + 2]-cycloadduct failed, and the only tractable result was alkene isomerization to deliver known^15^ dienones **20** (a sesquiterpenoid from *Enterospermum madagascarensis*)^16^ and **21**.

It was concluded that the dienophile in the C-15 methyl-bearing enone would require electronic activation, and so, the route outlined in Scheme 1 was initiated. The first phase of the synthesis proceeded smoothly, with little in the five steps^17−19^ to hydroxypyrone **26** (Scheme 3) requiring comment. The *anti-*crotylation^20^ product (**S2**, see Supporting Information) derived from aldehyde **22** was targeted in order to allow the two substituents in the projected cycloadduct to be both *cis* and *exo-*disposed,^21^ thus easing the course of the cycloaddition. The use of classical methods for initiating formation and cycloaddition of the oxidopyrylium derived from **26** were unproductive or inefficient; however, Suga’s mild conditions using Boc-anhydride to activate the hydroxyl group, in combination with triethylamine as catalyst,^22^ produced the separable diastereomeric *exo-*cycloadducts **27** and **28** in ∼80% combined yield. In this reaction, the major isomer is that expected from Sammes’ studies of related reactions.^23^ Conjugate addition of lithium dimethylcuprate to the more exposed α-face of the enone, followed by desilylation and oxidation, afforded tricyclic diketone **30**, from which it was envisaged that mild treatment with basic or Lewis acidic reagents would induce enone formation with cleavage of the C-1–O bond (→ **31**). In the event, no reagent combinations were able to achieve this elimination, presumably because any ring-opening remained hidden by rapid reclosure of the so-formed 3°-alcohol.

**Scheme 3:**
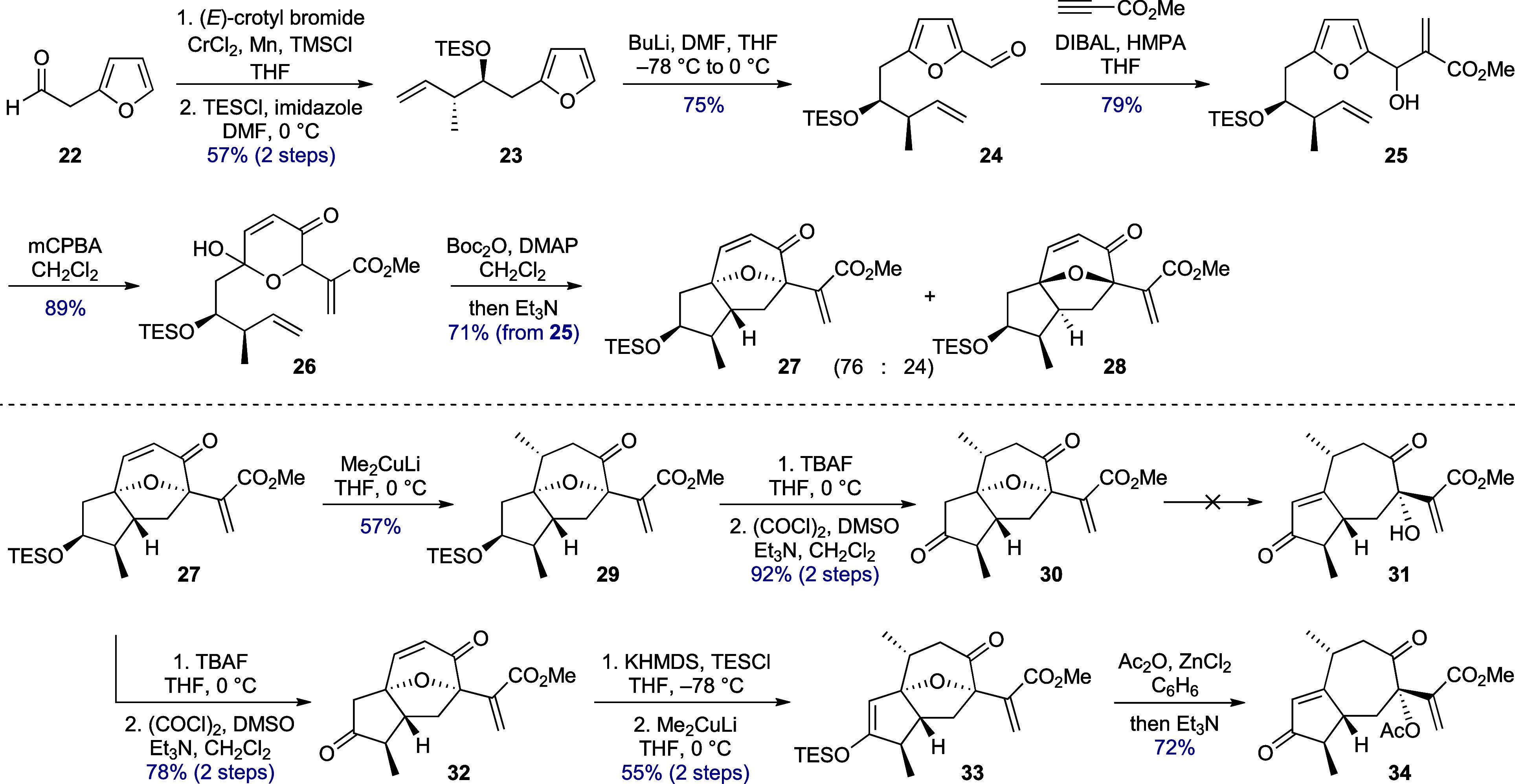
Oxidopyrylium Cycloaddition and Elimination of the 1,7-Oxa Bridge

Taking inspiration from Mascareñas’ “push–pull” approach to oxa-bridge cleavage in related structures,^24^ the steps from cycloadduct **27** were reordered to enable the two ketones (in **32**) to be distinguished. Thus, the “pushing” silyl enol ether functionality was introduced prior to methyl 1,4-addition, and then, the Lewis acidic “pulling” conditions were applied to so-formed **33** with the inclusion of acetic anhydride to capture liberated 3°-alcohol and prevent its recyclization. This was effective, and the acetoxy enedione **34** was accessed in readiness for the proposed IMDA-equivalent step. Again, forward progress was thwarted since all attempts to effect cycloaddition using variants of the conditions used for the formation of tetracycle **15** led to decomposition. Here, reaction mixtures usually developed a purple coloration that was attributed to azulene formation by acetate elimination and aerial oxidation.

To obviate the need to add activating reagents for the IMDA cycloaddition and reduce the likelihood of azulene formation, a preformed diene component was targeted and the oxidation state of the substrate was lowered (Scheme 4). First, the C-8 ketone was reduced, and the resulting alcohol acetylated during the β-elimination step to form the enone (→ **35**). Luche reduction of the C-3 ketone then gave cyclopentenol **36**. For the dehydration step, Burgess reagent was chosen based on its preference for a *syn-*elimination mechanism;^25^ from this, a major cyclopentadiene regioisomer **37** was obtained. Once more, the incorrect placement of the diene was not envisaged to be problematic and, indeed, heating this substrate in toluene (in a pressure tube) initiated clean IMDA cycloaddition, with the tetracyclic cycloadduct **38** this time being perfectly stable toward chromatographic purification.

**Scheme 4:**
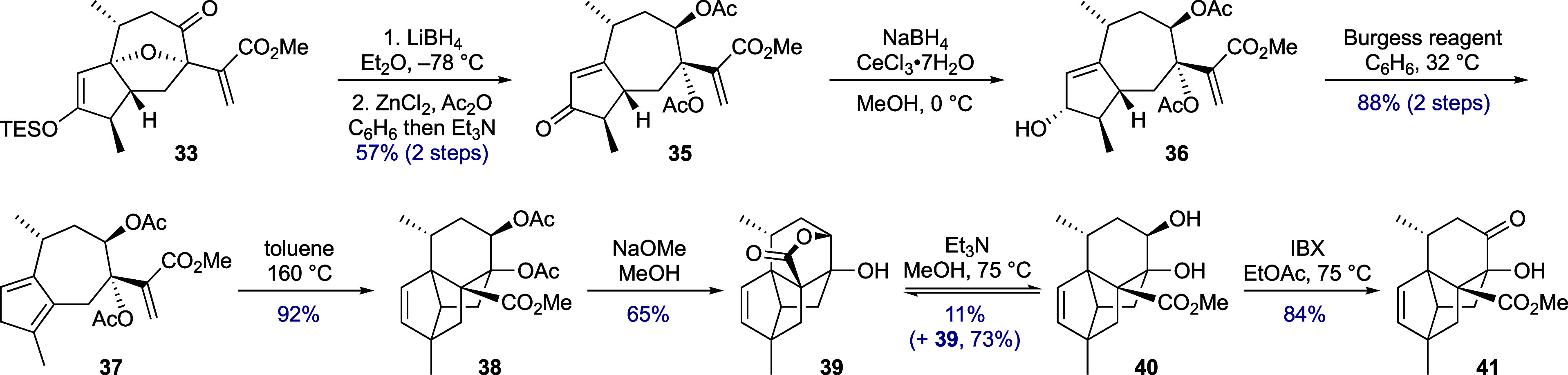
Completion of the Tetracyclic Core and Progress towards Daphnenoid A

From this point, a potential end-sequence to daphnenoid A was projected: (i) double deacetylation, (ii) oxidation of the C-8 alcohol to the required enone, and (iii) formation of the C-3 carbonyl group by either hydroboration/oxidation or Wacker oxidation. In practice, methanolysis of triester **38** gave pentacyclic lactone **39**, similar in structure to artatrovirenol A. Heating this lactone with triethylamine in methanol established an equilibrium with diol **40**, allowing a sample of the latter to be separated. An attempt to access the C-8–C-10 enone directly with IBX^26^ led to decomposition, but under milder conditions, ketone **41** was formed, which is currently our most advanced intermediate en route to daphnenoid A.^27,28^

The synthetic chemistry summarized in Schemes 3 and 4 comprises a 15-step sequence to tetracyclic intermediate **38**, from which some redox adjustments remain necessary to reach daphnenoid A. It is, however, conceivable that a variant of the route to form compound **15** (Scheme 2) could deliver the complete carbon skeleton more quickly, from which chemical and enzymatic C–H hydroxylation^29^ would then offer access to this interesting class of tetracarbocyclic sesquiterpenoids and their analogues. Efforts to that end are ongoing in our laboratories.

## Data Availability

The data underlying this study are available in the published article and its Supporting Information.

## Supporting Information Available

Experimental procedures, characterization data, copies of ^1^H and ^13^C NMR spectra for all compounds, and crystallographic details (PDF)
