# Supplementary material for: Routes to Advanced Intermediates in the Synthesis of Tetracarbocyclic Sesquiterpenoids Daphnenoid A and Artatrovirenols A and B
Source: Org Lett. 2024 Feb 19;26(8):1556–60. doi: 10.1021/acs.orglett.3c04199 (PMC10913076; doi:10.1021/acs.orglett.3c04199)
Supplement: Supplementary file 1 — ol3c04199_si_001.pdf [file ol3c04199_si_001.pdf]

Supporting Information

Routes to Advanced Intermediates in the Synthesis of Tetracarbocyclic Sesquiterpenoids Daphnenoid A and Artatrovirenols A and B

Jiarui Zong,[1] Kirsten E. Christensen,[1] and Jeremy Robertson[1,2*]

[1]Department of Chemistry, University of Oxford, Chemistry Research Laboratory, Mansfield Road, Oxford, OX1 3TA (UK)
[2]Oxford Suzhou Centre for Advanced Research, Ruo Shui Road, Suzhou Industrial Park, Jiangsu, 215123 (P. R. China)

* jeremy.robertson@chem.ox.ac.uk

Table of Contents

General methods S2

Experimental procedures and characterisation data

(1) For Scheme 2 S3

(2) For Scheme 3 S7

(3) For Scheme 4 S15

^{1}H and ^{13}C NMR spectra for synthesised compounds S21

Selected NOESY spectra S55

Crystallographic details for 16 S58

General methods

Procedures are presented in the order given in the Schemes.

All solvents for anhydrous reactions were obtained dry from Grubbs solvent dispenser units after being passed through an activated alumina column under argon. Commercially available reagents were, in general, used as supplied; amines and dipolar aprotic solvents were purified by standard methods before use. "Petrol" refers to the fraction of light petroleum ether boiling in the range of 30–40 °C; "ether" refers to diethyl ether. Unless stated otherwise, all reactions were carried out in oven-dried glassware and under an inert atmosphere (N_2 or Ar as specified); reactions performed above ambient temperature were heated using a thermostatically-controlled oil bath. Silica gel chromatography was carried out using Geduran Silicagel 60, particle size 40–63 µm. Thin-layer chromatography (TLC) was conducted using Merck aluminium-backed Silicagel 60 F254 fluorescent treated silica; visualisation was enabled by UV light (λ_{max} = 254 nm) and staining with potassium permanganate or phosphomolybdic acid solution to give the retention factors (R_f) quoted. Compound names are as generated by PerkinElmer ChemDraw Professional 22.2. Melting points (mp) were recorded (uncorrected) in degrees Celsius (°C), using a Griffin MFB-700-010U melting point apparatus. IR spectra were recorded on a Bruker Tensor 27 FT-IR spectrometer as a thin film on a diamond ATR module; only selected absorption maxima (ν_{max}) are reported, in wavenumbers (cm^{-1}). ^1H and ^{13}C NMR spectra were recorded using Bruker AVIII 700, AVIII 600, NEO 600, AVIIIHD 500, NEO 400 Nanobay, and AVIIIHD 400 spectrometers using the solvents specified. Chemical shifts are quoted in ppm downfield of tetramethylsilane (δ = 0) and referenced in MestReNova to the appropriate solvent peak: $CDCl_3$, 7.26/77.16; C_6D_6, 7.16/128.06. Coupling constants (*J*) are quoted in Hz, rounded to the nearest 0.5 Hz. All ^1H NMR spectra are reported as follows: ppm (number of protons, multiplicity, coupling constants). Structural assignments were made using a combination of 1D NMR experiments supported by 2D NOESY, HSQC, and HMBC experiments where necessary. High-resolution mass spectra (HRMS) were recorded by the staff at the Chemistry Research Laboratory (University of Oxford) using a Bruker Daltonics MicroTOF spectrometer; mass-to-charge ratios (*m*/*z*) are reported in Daltons.

Experimental procedures and characterisation data

1. For Scheme 2

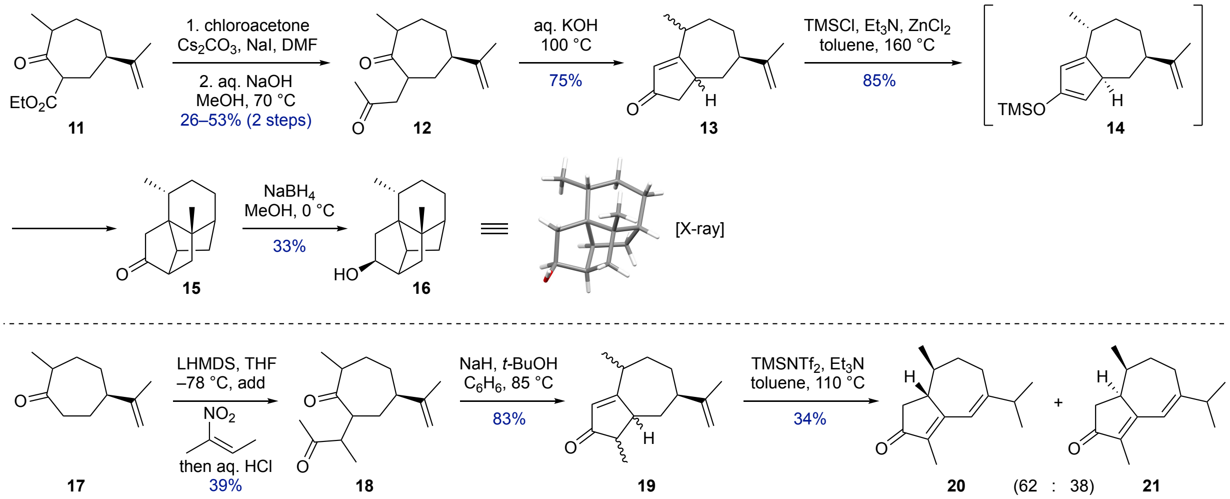

11 12 13 14 15 16 17 18 19 20 21

*Ethyl (6R)-3-methyl-2-oxo-1-(2-oxopropyl)-6-(prop-1-en-2-yl)cycloheptane-1-carboxylate (**S1**)*

To a solution of keto-ester **11** (0.14 g, 0.59 mmol) in DMF (6 mL) was added NaI (0.11 g, 0.73 mmol), Cs_2CO_3 (0.34 g, 1.0 mmol) and chloroacetone (60 µL, 0.75 mmol). The mixture was stirred at RT for 18 h then water (10 mL) was added. The mixture was extracted with ether (3 × 20 mL) and the combined organic extracts were washed with brine (2 × 10 mL), dried over Na_2SO_4, and concentrated. The residue was purified by chromatography (pentane/ethyl acetate, 5:1) to give diketone **S1** as a pale yellow oil (93.5 mg, 54%) and as a single diastereomer (not assigned). [Larger scale reactions gave **S1** (210 mg, 36% and 2.31 g, 46%) from **11** (476 mg, 2.00 mmol and 4.10 g, 17.2 mmol respectively)]. R_f 0.15 (pentane/ethyl acetate, 5:1); $[\alpha]_D^{25}$ –56 (*c* 0.57, CH_2Cl_2); IR ν_{max}/cm^{-1} 2979m, 2933m, 1734s, 1706s, 1208m; ^1H NMR (500 MHz, $CDCl_3$) δ_H 4.68 – 4.64 (2H, m), 4.24 – 4.15 (2H, m), 3.14 (1H, d, *J* = 17.5 Hz), 2.99 (1H, d, *J* = 17.5 Hz), 2.92 (1H, dtd, *J* = 11.0, 6.5, 2.0 Hz), 2.17 – 2.09 (2H, m) overlaying 2.13 (3H, s), 1.97 (1H, dd, *J* = 14.5, 10.5 Hz), 1.89 – 1.82 (1H, m), 1.73 – 1.66 (4H, m), 1.64 – 1.57 (1H, m), 1.53 (1H, tdd, *J* = 13.0, 11.5, 3.5 Hz), 1.27 (3H, t, *J* = 7.0 Hz), 1.13 (3H, d, *J* = 6.5 Hz); ^{13}C NMR (126 MHz, $CDCl_3$) δ_C 211.7, 205.3, 172.6, 150.7, 109.0, 61.5, 61.3, 51.9, 45.8, 44.6, 39.0, 36.2, 33.4, 30.4, 20.7, 18.0, 14.3; HRMS (ESI+) *m/z* $[M+Na]^+$ calcd for $C_{17}H_{26}NaO_4$, 317.1723; found, 317.1727.

*(4R)-7-Methyl-2-(2-oxopropyl)-4-(prop-1-en-2-yl)cycloheptan-1-one (**12**)*

To a solution of diketo-ester **S1** (0.14 g, 0.47 mmol) in methanol (10 mL) was added a solution of NaOH (45.0 mg, 1.13 mmol) in water (2.5 mL). The mixture was stirred at 80 °C for 3 h then cooled to RT, diluted with hydrochloric acid (10

mL, 1.0 M) and the aqueous layer extracted with ether (3 × 10 mL). The combined organic extracts were washed with brine (2 × 10 mL), dried over Na_2SO_4, and concentrated to give the title compound, an oil, as a complex mixture of diastereomers (77.5 mg, 73%) which was used directly in the next step without purification. [A larger scale reaction gave **12** (1.74 g, 99%) from **S1** (2.31 g, 7.85 mmol)]. R_f 0.31 (pentane/ethyl acetate, 4:1); IR ν_{max}/cm^{-1} 2929m, 1706s, 1645w, 1465m, 891m; ^{1}H NMR (400 MHz, $CDCl_3$) δ_H (integrals approximate) 4.70 – 4.61 (2H, m), 3.30 – 3.02 (2H, m), 2.92 – 2.83 (0.5H, m), 2.55 – 2.44 (0.5H, m), 2.39 – 2.25 (1H, m), 2.24 – 2.09 (4H, m), 1.97 – 1.85 (1.5H, m), 1.82 – 1.46 (6.5H, m), 1.39 – 1.28 (0.5H, m), 1.27 – 1.18 (0.5H, m), 1.17 – 1.01 (3H, m); HRMS (ESI+) *m/z* $[M+K]^+$ calcd for $C_{14}H_{22}KO_2$, 261.1251; found, 261.1261.

*(7R)-4-Methyl-7-(prop-1-en-2-yl)-4,5,6,7,8,8a-hexahydroazulen-2(1H)-one (**13**)*

To a solution of diketone **12** (53.5 mg, 0.241 mmol) in ethanol (10 mL) was added KOH (13.5 mg, 0.241 mmol) and the suspension was stirred at 90 °C for 18 h. The mixture was cooled to RT, quenched with saturated aqueous NH_4Cl solution (10 mL), and extracted with ether (3 × 10 mL). The combined organic extracts were dried over Na_2SO_4 and concentrated to give a complex mixture of isomers of the title compound (including diastereomers and regioisomers) (37 mg, 75%) which was used directly for next step without purification. [A larger scale reaction gave **13** (1.20 g, 75%) from **12** (1.74 g, 7.85 mmol)]. R_f 0.20 (pentane/ethyl acetate, 4:1); IR ν_{max}/cm^{-1} 2971m, 1701s, 1645w, 1604m, 890m; ^{1}H NMR (400 MHz, $CDCl_3$) δ_H (integrals approximate) 6.01 – 5.87 (1H, m), 4.76 – 4.56 (2H, m), 3.24 – 3.01 (0.5H, m), 2.92 – 2.28 (2.5H, m), 2.27 – 1.76 (4H, m), 1.75 – 1.65 (3.5H, m), 1.64 – 1.24 (3H, m), 1.23 – 0.95 (3.5H, m); HRMS (ESI+) *m/z* $[M+Na]^+$ calcd for $C_{14}H_{20}NaO$, 227.1406; found, 227.1416.

*(1R,3aS,4R,7R,8aS,9S)-4,9-Dimethylhexahydro-1H-3a,7,1-(epiethane[1,1,2]triyl)azulen-2(3H)-one (**15**)*

A stirred suspension of enone mixture **13** (0.20 g, 0.98 mmol), $ZnCl_2$ (1.36 g, 10.0 mmol), triethylamine (1.4 mL, 10 mmol) and chlorotrimethylsilane (1.3 mL, 10 mmol) in toluene (10 mL) in a pressure tube was heated at 160 °C for 24 h. The suspension was cooled to RT and washed with hydrochloric acid (20 mL, 2.75 M) then brine (20 mL). The organic phase was dried over Na_2SO_4 and concentrated to give cycloadduct **15** (0.17 g, 85%) as an orange oil which, from separate experiments, was found to decompose during purification attempts by chromatography. $[\alpha]_D^{25}$ +37 (*c* 0.13, CH_2Cl_2); IR ν_{max}/cm^{-1} 2932m, 2874m, 1749s, 1464w, 652w; ^{1}H NMR (600 MHz, $CDCl_3$) δ_H 2.28 (1H, d, *J* = 5.5 Hz), 2.24 (1H, d, *J* = 6.5 Hz), 2.14 (1H, dd, *J* = 19.0, 1.5 Hz), 1.90 (1H, dd, *J* = 19.0, 1.5 Hz), 1.80 – 1.69 (3H, m), 1.64 – 1.57 (1H, m), 1.57

– 1.43 (3H, m), 1.34 – 1.24 (2H, m), 1.12 (1H, dt, J = 13.0, 1.0 Hz), 0.99 (3H, s), 0.87 (3H, d, J = 6.5 Hz); ^{13}C NMR (151 MHz, $CDCl_3$) δ_C 217.0, 55.3, 54.9, 45.7, 45.1, 44.7, 38.0, 37.2, 28.7, 27.9, 26.9, 26.2, 19.0, 13.9; HRMS (ESI+) *m/z* $[M+Na]^+$ calcd for $C_{14}H_{20}NaO$, 227.1406; found, 227.1409.

*(1R,2S,3aS,4R,8aS,9S)-4,9-Dimethyloctahydro-1H-3a,7,1-(epiethane[1,1,2]triyl)azulen-2-ol (**16**)*

To a stirred solution of ketone **15** (0.17 g, 0.83 mmol) in methanol (10 mL) at 0 °C was added $NaBH_4$ (47.5 mg, 1.26 mmol) at 0 °C. The suspension was stirred for 2.5 h and quenched with saturated aqueous NH_4Cl solution (10 mL) then extracted with ether (3 × 10 mL). The combined organic extracts were washed with brine (10 mL), dried over Na_2SO_4, and concentrated. The residue was purified by chromatography (pentane/ethyl acetate, 6:1) to give alcohol **16** (56 mg, 33%) as a colourless solid which was recrystallised (from pentane/ethyl acetate) for X-ray analysis (see page S56). R_f 0.24 (pentane/ethyl acetate, 6:1); M.p. 94–96 °C; $[\alpha]_D^{25}$ +94 (*c* 0.07, CH_2Cl_2); IR ν_{max}/cm^{-1} 3344br, 2937s, 1468m, 1204m, 738m; ^{1}H NMR (500 MHz, $CDCl_3$) δ_H 4.20 – 4.15 (1H, m), 2.00 – 1.94 (2H, m), 1.81 (1H, dt, J = 6.0, 2.5 Hz), 1.71 – 1.67 (1H, m), 1.60 – 1.56 (2H, m), 1.49 – 1.36 (4H, m), 1.23 – 1.15 (2H, m), 1.06 (1H, ddd, J = 12.5, 5.5, 1.5 Hz), 0.96 (3H, s), 0.92 – 0.88 (1H, m), 0.84 (3H, d, J = 6.5 Hz); ^{13}C NMR (126 MHz, $CDCl_3$) δ_C 73.8, 56.8, 48.4, 46.8, 44.8, 44.6, 33.1, 32.8, 28.6, 28.4, 27.4, 26.4, 19.3, 13.0; HRMS (ESI+) *m/z* $[M+H]^+$ calcd for $C_{14}H_{23}O$, 207.1743; found, 207.1741.

*(4R)-7-Methyl-2-(3-oxobutan-2-yl)-4-(prop-1-en-2-yl)cycloheptan-1-one (**18**)*

To a solution of ketone **17**[1] (0.12 g, 0.72 mmol) in THF (10 mL) at –78 °C was added LHMDS (0.90 mL, 1.0 M in THF, 0.90 mmol). The mixture was stirred at this temperature for 1 h then 2-nitro-2-butene[2] (0.11 g, 1.1 mmol) was added and stirring continued at –78 °C for 1 h. The mixture was warmed to 0 °C, hydrochloric acid (20 mL, 3.0 M) was added, and the mixture was stirred at RT for 2 h then extracted with ether (3 × 20 mL). The combined organic extracts were washed with brine (10 mL), dried over $MgSO_4$, and concentrated. The residue was purified by chromatography (pentane/ethyl acetate, 20:1) to give diketone **18** (66 mg, 39%), a colourless oil, as a mixture of diastereomers. R_f 0.35 (pentane/ethyl acetate, 5:1); IR ν_{max}/cm^{-1} 2930m, 1703s, 1645w, 1458m, 891m; ^{1}H NMR (400 MHz, $CDCl_3$) δ_H (integrals approximate)

(1) Zong, J.; Robertson, J. An Enantiospecific Synthesis of 5-*epi*-α-Bulnesene. *Molecules* **2023**, *28*, 3900.
(2) Leroux, M.-L.; Le Gall, T.; Mioskowski, C. Enantioselective synthesis of α,α-disubstituted amines from nitroalkenes. *Tetrahedron: Asymmetry* **2001**, *12*, 1817–1823.

4.76 – 4.60 (2H, m), 3.25 – 2.33 (3H, m), 2.22 – 2.15 (3H, m), 2.14 – 2.02 (1H, m), 1.99 – 1.31 (8H, m), 1.30 – 0.94 (7H, m); HRMS (ESI+) *m/z* $[M+K]^+$ calcd for $C_{15}H_{24}KO_2$, 275.1408; found, 275.1412.

*(7R)-1,4-Dimethyl-7-(prop-1-en-2-yl)-4,5,6,7,8,8a-hexahydroazulen-2(1H)-one (**19**)*

To a solution of diketone **18** (0.12 g, 0.51 mmol) in benzene (5 mL) was added NaH (23 mg, 60% in mineral oil, 0.58 mmol) and *tert*-butanol (15 μL, 0.16 mmol) and the suspension was stirred at 90 °C for 15 min. The mixture was cooled to RT, quenched with cold water (10 mL), and extracted with ether (3 × 10 mL). The combined organic extracts were dried over Na_2SO_4 and concentrated to give a crude mixture of enone isomers of **19** (including diastereomers and regioisomers) (92.5 mg, 83%) which was used directly for the next step without purification. R_f 0.33 (pentane/ethyl acetate, 5:1); IR ν_{max}/cm^{-1} 2923m, 1703s, 1645m, 1604m, 1456m; ^1H NMR (400 MHz, $CDCl_3$) δ_H (integrals approximate) 5.97 – 5.84 (1H, m), 4.80 – 4.59 (2H, m), 2.93 – 2.31 (2H, m), 2.29 – 2.08 (1H, m), 2.06 – 1.62 (7H, m), 1.60 – 1.28 (2.5H, m), 1.26 – 0.98 (6.5H, m); HRMS (ESI+) *m/z* $[M+Na]^+$ calcd for $C_{15}H_{22}NaO$, 241.1563: found, 241.1558.

(8S,8aR*)-5-Isopropyl-3,8-dimethyl-6,7,8,8a-tetrahydroazulen-2(1H)-one (**20**)[3] and (8S*,8aS*)-5-Isopropyl-3,8-dimethyl-6,7,8,8a-tetrahydroazulen-2(1H)-one (**21**)[3]*

A solution of enone mixture **19** (12 mg, 0.055 mmol) in toluene (2 mL) containing *N*-(trimethylsilyl)bis(trifluoromethanesulfonyl)imide (102 mg, 0.290 mmol) was stirred at RT for 15 min. Triethylamine (35 μL, 0.25 mmol) was added and the mixture was heated to 110 °C for 30 min. The mixture was then cooled to RT and diluted with water (10 mL). The aqueous layer was extracted with ether (3 × 10 mL) and the combined organic extracts were dried over Na_2SO_4 and concentrated. The residue was purified by chromatography (pentane/ethyl acetate, 6:1) to give diastereomer **20** (2.5 mg, 21%) as a colourless oil. R_f 0.40 (pentane/ethyl acetate, 6:1); ^1H NMR (600 MHz, $CDCl_3$) δ_H 6.30 (1H, s), 2.63 (1H, br t, *J* = 8.0 Hz), 2.57 (1H, dd, *J* = 17.5, 6.5 Hz), 2.49 – 2.40 (2H, m), 2.15 – 2.10 (1H, m) overlaying 2.13 (1H, dd, *J* = 17.5, 2.0 Hz), 1.83 – 1.77 (1H, m), 1.74 (3H, d, *J* = 1.5 Hz), 1.62 – 1.54 (2H, m), 1.09 (3H, d, *J* = 7.0 Hz), 1.08 (3H, d, *J* = 7.0 Hz), 1.06 (3H, d, *J* = 6.5 Hz); ^{13}C NMR (151 MHz, $CDCl_3$) δ_C 209.0, 169.0, 162.8, 135.0, 119.1, 46.7, 42.3, 38.9, 38.8, 35.4, 27.0, 22.1, 21.4, 21.1, 8.3. Further elution gave diastereomer **21** (1.5 mg, 13%) as a colourless oil. R_f 0.33 (pentane/ethyl acetate, 6:1); ^1H NMR (600 MHz, $CDCl_3$) δ_H 6.33 (1H, s), 3.27 (1H, br s), 2.54 – 2.47 (1H, m) overlaying

(3) Blay, G.; Garcia, B.; Molina, E.; Pedro, J. R. Total Syntheses of Four Stereoisomers of 4α-Hydroxy-1β,7β-peroxy-10βH-guaia-5-ene. *Org. Lett.* **2005**, *7*, 3291–3294.

2.49 (1H, dd, J = 18.0, 7.0 Hz), 2.45 (1H, sept, J = 7.0 Hz), 2.27 – 2.18 (3H, m), 2.10 – 2.05 (1H, m), 1.75 (3H, d, J = 2.0 Hz), 1.56 – 1.49 (1H, m), 1.09 (3H, d, J = 7.0 Hz), 1.08 (3H, d, J = 7.0 Hz), 0.71 (3H, d, J = 7.0 Hz); ^{13}C NMR (151 MHz, $CDCl_3$) δ_C 209.3, 167.8, 161.7, 136.0, 119.3, 44.3, 39.8, 39.3, 34.9, 33.0, 28.1, 21.4, 21.3, 14.1, 8.2.

2. For Scheme 3

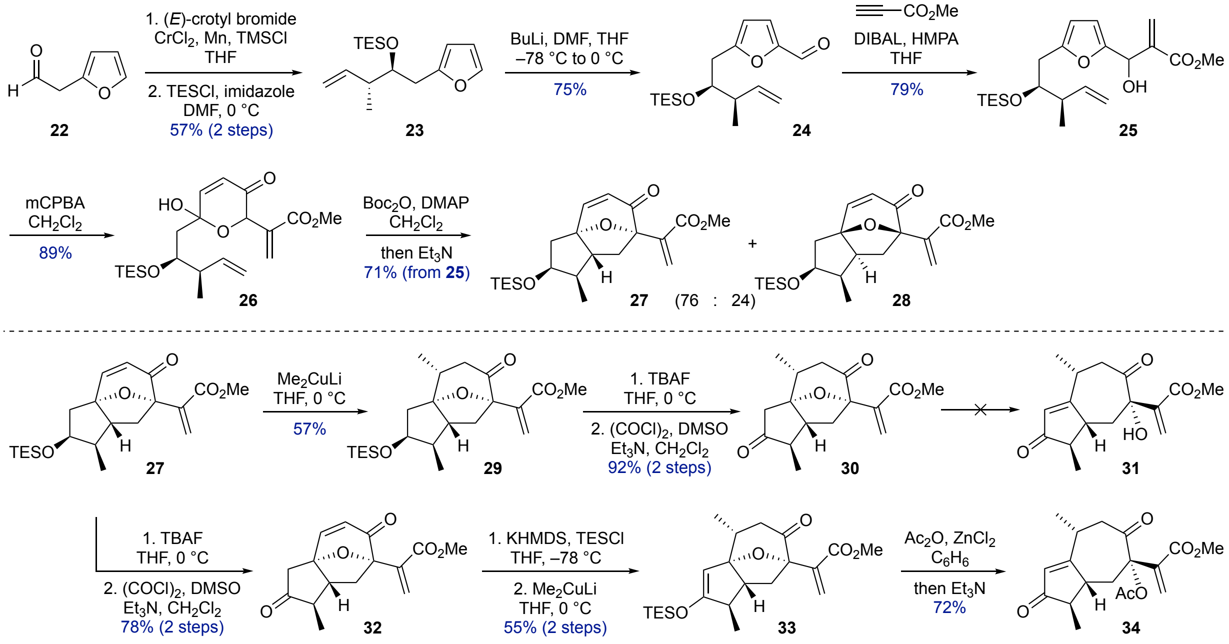

(2S,3R*)-1-(Furan-2-yl)-3-methylpent-4-en-2-ol (**S2**)*

A suspension of $CrCl_2$ (0.25 g, 2.0 mmol) and Mn powder (2.66 g, 48.4 mmol) in dry THF (125 mL) was stirred at RT for 30 min. To this mixture was added aldehyde **22** (crude, 2.5 g, ~23 mmol), crotyl bromide (5.94 mL, 57.7 mmol) and chlorotrimethylsilane (8.8 mL, 69 mmol) and stirring continued at RT for 3.5 h. Water (60 mL) was added and the mixture was stirred for a further 1 h. The organic phase was extracted with ether (3× 30 mL), then the combined extracts were dried over Na_2SO_4 and concentrated. The residue was purified by chromatography (pentane/ethyl acetate, 5:1) to give the alcohol **S2** as a colourless oil (2.44 g, 64%) as the *anti*-diastereomer (*anti/syn*, ~90:10). R_f 0.60 (pentane/ethyl acetate, 3:1); IR ν_{max}/cm^{-1} 3469w, 2924w, 1011s, 919s, 734s; ^{1}H NMR (400 MHz, $CDCl_3$) δ_H 7.34 (1H, dd, J = 2.0, 1.0 Hz), 6.31 (1H, dd, J = 3.0, 2.0 Hz), 6.12 (1H, dq, J = 3.0, 1.0 Hz), 5.83 (1H, ddd, J = 17.0, 10.5, 8.0 Hz), 5.19 – 5.04 (2H, m), 3.76 (1H, ddd, J = 8.5, 5.5, 4.0 Hz), 2.86 (1H, ddd, J = 15.0, 4.0, 1.0 Hz), 2.73 (1H, dd, J = 15.0, 8.5 Hz), 2.36 – 2.21 (1H, m), 1.10 (3H, d, J = 7.0 Hz); ^{13}C NMR (101 MHz, $CDCl_3$) δ_C 153.1, 141.7, 139.8, 116.5, 110.5, 107.1, 73.5, 43.4, 33.4, 16.4; HRMS (ESI+) m/z [M+H]$^+$ calcd for $C_{10}H_{15}O_2$, 167.1067; found, 167.1065.

Triethyl[[(2S,3R*)-1-(furan-2-yl)-3-methylpent-4-en-2-yl]oxy]silane (**23**)*

A solution of alcohol **S1** (2.44 g, 14.7 mmol) in dry DMF (100 mL) was cooled to 0 °C and chlorotriethylsilane (4.9 mL, 29 mmol) and imidazole (2.50 g, 36.7 mmol) were added. The mixture was stirred at 0 °C for 1 h then saturated aqueous NH_4Cl solution (60 mL) was added. The mixture was extracted with ether (3× 30 mL) and the combined organic extracts were washed with water (50 mL) then brine (50 mL), dried over Na_2SO_4, and concentrated. The residue was purified by chromatography (pentane/ethyl acetate, 10:1) to give the title compound as a colourless oil (3.67 g, 89%). R_f 0.80 (pentane/ethyl acetate, 10:1); IR ν_{max}/cm^{-1} 2958w, 2878w, 1010m, 915m, 727s; ^{1}H NMR (400 MHz, $CDCl_3$) δ_H 7.30 (1H, dd, *J* = 2.0, 1.0 Hz), 6.27 (1H, dd, *J* = 3.0, 2.0 Hz), 6.01 (1H, dd, *J* = 3.0, 1.0 Hz), 5.83 (1H, ddd, *J* = 17.0, 10.5, 8.0 Hz), 5.10 – 4.96 (2H, m), 3.95 (1H, ddd, *J* = 7.0, 6.0, 3.0 Hz), 2.74 (1H, dd, *J* = 14.5, 6.5 Hz), 2.67 (1H, dd, *J* = 14.5, 6.5 Hz), 2.32 – 2.21 (1H, m), 1.04 (3H, d, *J* = 7.0 Hz), 0.91 (9H, ~t, *J* = 8.0 Hz), 0.56 – 0.49 (6H, m); ^{13}C NMR (101 MHz, $CDCl_3$) δ_C 153.6, 141.0, 140.2, 115.3, 110.4, 106.9, 74.7, 43.3, 33.6, 15.9, 7.0, 5.1; HRMS (ESI+) *m/z* $[M+Na]^+$ calcd for $C_{16}H_{28}NaO_2Si$, 303.1751; found, 303.1749.

5-[(2S,3R*)-3-Methyl-2-[(triethylsilyl)oxy]pent-4-en-1-yl]furan-2-carbaldehyde (**24**)*

A solution of furan derivative **23** (2.51 g, 8.95 mmol) in dry THF (100 mL) was cooled to –78 °C and butyllithium (11.9 mL, 1.6 M in hexanes, 19.0 mmol) added dropwise. The mixture was warmed to –5 °C and stirred for 45 min then re-cooled to –78 °C and dimethylformamide (2.0 mL, 26 mmol) added. The solution was stirred for 30 min, warmed to RT and stirred for a further 2 h. Saturated aqueous NH_4Cl solution (60 mL) was added and the mixture was extracted with ether (3× 30 mL). The organic extracts were washed with water (30 mL) then brine (30 mL), and were then dried over Na_2SO_4 and concentrated. The residue was purified by chromatography (pentane/ethyl acetate, 10:1) to give the title compound (2.07 g, 75%) as a colourless oil. R_f 0.41 (pentane/ethyl acetate, 10:1); IR ν_{max}/cm^{-1} 2959w, 1683s, 1519s, 1020m, 741s; ^{1}H NMR (600 MHz, $CDCl_3$) δ_H 9.54 (1H, s), 7.17 (1H, d, *J* = 3.5 Hz), 6.28 (1H, dd, *J* = 3.5, 1.0 Hz), 5.81 (1H, ddd, *J* = 17.5, 10.5, 7.5 Hz), 5.10 – 4.97 (2H, m), 4.06 (1H, ddd, *J* = 7.5, 5.5, 3.5 Hz), 2.81 (1H, dd, *J* = 15.0, 5.5 Hz), 2.77 (1H, dd, *J* = 15.0, 7.5 Hz), 2.35 – 2.25 (1H, m), 1.05 (3H, d, *J* = 7.0 Hz), 0.88 (9H, t, *J* = 8.0 Hz), 0.54 – 0.44 (6H, m); ^{13}C NMR (151 MHz, $CDCl_3$) δ_C 177.1, 161.5, 152.1, 139.8, 123.4, 115.6, 110.9, 74.1, 43.7, 33.6, 14.9, 7.0, 5.0; HRMS (ESI+) *m/z* $[M+Na]^+$ calcd for $C_{17}H_{28}NaO_3Si$, 331.1700; found, 331.1700.

Methyl 2-[hydroxy(5-{(2S,3R*)-3-methyl-2-[(triethylsilyl)oxy]pent-4-en-1-yl}furan-2-yl)methyl]acrylate (**25**)*

A solution of DIBAL (17.5 mL, 1.0 M in hexane, 17.5 mmol) and HMPA (9.1 mL, 50 mmol) in THF (100 mL) was cooled to 0 °C and stirred for 30 min. Methyl propiolate (1.7 mL, 19 mmol) was added and the mixture was stirred at 0 °C for 2.5 h. A solution of aldehyde **24** (2.16 g, 7.00 mmol) in dry THF (10 mL) was added and the mixture was warmed to RT then stirred for 18 h. The mixture was cooled to 0 °C and hydrochloric acid (30 mL, 1.0 M) was added then the mixture was extracted with ether (3× 30 mL). The combined organic extracts were washed with hydrochloric acid (30 mL, 1.0 M) then saturated aqueous $NaHCO_3$ solution (30 mL), dried over Na_2SO_4, and concentrated. The residue was purified by chromatography (pentane/ethyl acetate, 6:1) to give the title compound (2.19 g, 79%) as a colourless oil, apparently (NMR) as a single diastereomer (not assigned). R_f 0.35 (pentane/ethyl acetate, 6:1); IR ν_{max}/cm^{-1} 3488w, 2959m, 1727s, 1016s, 742s; ^{1}H NMR (700 MHz, $CDCl_3$) δ_H 6.38 (1H, dt, *J* = 1.5, 1.0 Hz), 6.13 (1H, d, *J* = 3.0 Hz), 5.97 – 5.94 (2H, m), 5.81 (1H, ddd, *J* = 17.5, 10.5, 7.5 Hz), 5.54 (1H, s), 5.10 – 4.96 (2H, m), 3.92 (1H, tdd, *J* = 7.0, 5.5, 3.5 Hz), 3.76 (3H, s), 2.92 (1H, s), 2.71 (1H, dd, *J* = 15.0, 5.5 Hz), 2.64 (1H, ddd, *J* = 15.0, 7.0, 2.0 Hz), 2.28 – 2.22 (1H, m), 1.03 (3H, d, *J* = 7.0 Hz), 0.91 (9H, t, *J* = 8.0 Hz), 0.55 – 0.45 (6H, m); ^{13}C NMR (176 MHz, $CDCl_3$) δ_C 166.6, 153.7, 152.7, 140.2, 139.7, 126.8, 115.3, 108.1, 107.9, 74.6, 67.6, 52.1, 43.4, 33.7, 15.8, 7.0, 5.1; HRMS (ESI+) *m/z* $[M+K]^+$ calcd for $C_{21}H_{34}KO_5Si$, 433.1807; found, 433.1804.

Methyl 2-(6-hydroxy-6-{(2S,3R*)-3-methyl-2-[(triethylsilyl)oxy]pent-4-en-1-yl}-3-oxo-3,6-dihydro-2H-pyran-2-yl)acrylate (**26**)*

A solution of furfurylic alcohol **25** (0.542 g, 1.37 mmol) in dichloromethane (20 mL) was cooled to 0 °C and mCPBA (0.347 g, 75% w/w, 1.51 mmol) was added. The mixture was stirred at 0 °C for 1 h and at RT for 2 h, then saturated aqueous $NaHCO_3$ solution (20 mL) was added and the mixture was extracted with dichloromethane (3 × 20 mL). The combined organic extracts were washed with water (20 mL) and brine (20 mL), then dried over Na_2SO_4, and concentrated. The residue was purified by chromatography (pentane/ethyl acetate, 5:1) to give a diastereomer of the title compound (0.245 g, 44%) as a colourless oil. R_f 0.35 (pentane/ethyl acetate, 5:1); IR ν_{max}/cm^{-1} 3465w, 2970m, 1730s, 1071s, 743s; ^{1}H NMR (400 MHz, $CDCl_3$) δ_H 6.70 (1H, d, *J* = 10.0 Hz), 6.42 (1H, d, *J* = 1.0 Hz), 6.02 (1H, d, *J* = 10.0 Hz), 5.87 (1H, t, *J* = 1.0 Hz), 5.81 (1H, s), 5.66 (1H, ddd, *J* = 17.5, 10.0, 6.0 Hz), 5.46 (1H, d, *J* = 1.0 Hz), 5.09 – 5.43 (2H, m), 4.53 (1H, ddd, *J* = 11.0, 4.0, 2.5 Hz), 3.76 (3H, s), 2.53 – 2.43 (1H, m), 1.91 (1H, dd, *J* = 14.0, 11.0 Hz), 1.72 (1H, dd, *J* = 14.0, 2.5 Hz), 1.05 (3H, d, *J* = 7.0 Hz), 0.96 (9H, t, *J* = 8.0 Hz), 0.66 (6H, q, *J* = 8.0 Hz); ^{13}C NMR (101 MHz, $CDCl_3$)

δ_C 194.5, 165.8, 147.9, 139.6, 137.0, 128.6, 125.7, 115.5, 94.5, 74.2, 73.0, 52.2, 43.1, 37.8, 11.3, 6.8, 5.2; HRMS (ESI+) *m/z* $[M+Na]^+$ calcd for $C_{21}H_{34}NaO_6Si$, 433.2017; found, 433.2006. Further elution gave a second diastereomer (along with a third, minor diastereomer) of the title compound (255 mg, 45%) as a colourless oil. R_f 0.25 (pentane/ethyl acetate, 5:1); IR ν_{max}/cm^{-1} 3480w, 2955m, 1730s, 1691m, 743s; ^{1}H NMR (400 MHz, $CDCl_3$) δ_H 6.81 (1H, d, *J* = 10.0 Hz), 6.42 (1H, d, *J* = 1.0 Hz), 6.04 (1H, d, *J* = 10.0 Hz), 5.92 (1H, t, *J* = 1.0 Hz), 5.70 (1H, ddd, *J* = 17.5, 10.0, 7.0 Hz), 5.47 (1H, d, *J* = 1.0 Hz), 5.08 – 5.00 (2H, m), 3.98 (1H, ddd, *J* = 7.0, 5.5, 3.0 Hz), 3.75 (3H, s), 2.69 – 2.61 (1H, m), 2.05 (1H, dd, *J* = 14.5, 7.0 Hz), 1.94 (1H, dd, *J* = 14.5, 3.0 Hz), 1.00 (3H, d, *J* = 7.0 Hz) overlaying 0.98 (9H, t, *J* = 8.0 Hz), 0.65 (6H, q, *J* = 8.0 Hz); ^{13}C NMR (101 MHz, $CDCl_3$) δ_C 194.2, 165.7, 147.5, 140.7, 136.8, 129.0, 125.8, 115.7, 95.1, 74.4 (two carbons), 52.2, 43.3, 40.8, 13.5, 7.0, 5.2; HRMS (ESI+) *m/z* $[M+Na]^+$ calcd for $C_{21}H_{34}NaO_6Si$, 433.2017; found, 433.2006.

Methyl 2-{(1R,2S*,3aR*,7S*,8aR*)-1-methyl-6-oxo-2-[(triethylsilyl)oxy]-2,3,8,8a-tetrahydro-1H-3a,7-epoxyazulen-7(6H)-yl}acrylate (**27**) and methyl 2-{(1R*,2S*,3aS*,7R*,8aS*)-1-methyl-6-oxo-2-[(triethylsilyl)oxy]-2,3,8,8a-tetrahydro-1H-3a,7-epoxyazulen-7(6H)-yl}acrylate (**28**)*

On large scale, the previous reaction was performed with alcohol **25** (2.10 g, 5.32 mmol) to give the crude mixture of hydroxypyranones **26** (2.22 g) as a yellow oil. This was immediately dissolved in dry dichloromethane (80 mL), cooled to 0 °C, then DMAP (0.13 g, 1.1 mmol) and di-*tert*-butyldicarbonate (1.74 g, 7.97 mmol) were added. After 1 h triethylamine (2.8 mL, 20 mmol) was added, then the solution was warmed to RT and stirred for 2.5 h. Saturated aqueous NH_4Cl solution (40 mL) was added and the mixture was extracted with ether (3 × 30 mL). The organic extracts were washed with brine (30 mL), dried over Na_2SO_4, and concentrated. The residue was purified by chromatography (pentane/ethyl acetate, 3:1) to give the first cycloadduct diastereomer **27** (1.12 g, 54% from **25**) as a pale yellow oil. R_f 0.60 (pentane/ethyl acetate, 3:1); IR ν_{max}/cm^{-1} 2954w, 1733s, 1698s, 1046m, 742s; ^{1}H NMR (400 MHz, $CDCl_3$) δ_H 7.26 (1H, d, *J* = 9.5 Hz), 6.31 (1H, d, *J* = 1.0 Hz), 5.99 (1H, d, *J* = 9.5 Hz), 5.94 (1H, d, *J* = 1.0 Hz), 4.28 (1H, t, *J* = 4.5 Hz), 3.77 (3H, s), 2.48 (1H, ddd, *J* = 11.0, 8.5, 3.0 Hz), 2.36 (1H, dd, *J* 13.5, 8.5 Hz) overlaying 2.34 (1H, dd, *J* = 15.0, 5.0 Hz), 2.07 (1H, dd, *J* = 13.5, 3.0 Hz) overlaying 2.04 (1H, d, *J* = 15.0 Hz), 1.76 (1H, dqd, *J* = 10.5, 7.0, 4.0 Hz), 1.00 (3H, d, *J* = 6.5 Hz), 0.97 (9H, t, *J* = 8.0 Hz), 0.64 (6H, q, *J* = 8.0 Hz); ^{13}C NMR (101 MHz, $CDCl_3$) δ_C 195.6, 166.2, 157.1, 139.7, 125.9, 125.4, 91.6, 91.0, 79.0, 54.6, 52.1, 47.3, 45.7, 36.0, 12.7, 7.0, 5.0; HRMS (ESI+) *m/z* $[M+Na]^+$ calcd for $C_{21}H_{32}NaO_5Si$, 415.1911; found, 415.1899. Further elution gave a mixture of diastereomer **28** and an inseparable minor

diastereomer (unassigned) (0.35 g, 17%) as a pale yellow oil. R_f 0.45 (pentane/ethyl acetate, 3:1); IR ν_{max}/cm^{-1} 2942w, 1733s, 1698s, 1010w, 744s; ^{1}H NMR (400 MHz, $CDCl_3$) δ_H 7.20 (1H, d, J = 9.5 Hz), 6.29 (1H, d, J = 1.5 Hz), 5.95 (1H, d, J = 9.5 Hz) overlaying 5.94 (1H, d, J = 1.5 Hz), 4.34 (1H, ddd, J = 9.0, 7.5, 6.0 Hz), 3.78 (3H, s), 2.55 (1H, ddd, J = 9.0, 7.5, 4.0 Hz), 2.37 (1H, dd, J = 14.0, 3.5 Hz), 2.29 (1H, dd, J = 14.0, 9.0 Hz), 2.22 (1H, dd, J = 14.0, 7.5 Hz) overlaying 2.20 – 2.17 (1H, m), 2.12 (1H, dd, J = 14.0, 8.5 Hz), 0.96 (9H, t, J = 8.0 Hz), 0.88 (3H, d, J = 7.5 Hz), 0.59 (6H, q, J = 8.0 Hz); ^{13}C NMR (101 MHz, $CDCl_3$) δ_C 195.7, 166.4, 156.9, 138.7, 125.9, 125.2, 90.7, 88.6, 76.8, 52.1, 50.0, 41.5, 40.1, 32.5, 9.7, 6.9, 4.9; HRMS (ESI+) *m/z* $[M+Na]^+$ calcd for $C_{21}H_{32}NaO_5Si$, 415.1911; found, 415.1901.

Methyl 2-{(1R,2S*,3aR*,4R*,7S*,8aR*)-1,4-dimethyl-6-oxo-2-[(triethylsilyl)oxy]hexahydro-1H-3a,7-epoxyazulen-7(4H)-yl}acrylate (**29**)*

To a suspension of CuI (0.19 g, 1.0 mmol) in dry THF (25 mL) at 0 °C was added methyllithium (1.3 mL, 1.6 M in ether, 2.1 mmol). The solution was stirred for 30 min then a solution of enone **27** (0.39 g, 0.99 mmol) in dry THF (2 mL) was added dropwise. The mixture was stirred at 0 °C for 1 h then saturated aqueous NH_4Cl solution (10 mL) was added; the mixture was extracted with ether (3 × 10 mL), the organic extracts were washed with brine (10 mL) then dried over Na_2SO_4 and concentrated. The residue was purified by chromatography (pentane/ethyl acetate, 10:1) to give the title compound (0.23 g, 57%) as a colourless oil. R_f 0.35 (pentane/ethyl acetate, 10:1); IR ν_{max}/cm^{-1} 3027w, 1731s, 1438m, 1035m, 743m; ^{1}H NMR (400 MHz, $CDCl_3$) δ_H 6.27 (1H, d, J = 1.5 Hz), 5.96 (1H, d, J = 1.5 Hz), 4.25 (1H, td, J = 4.5, 2.5 Hz), 3.74 (3H, s), 2.90 (1H, dd, J = 15.5, 7.5 Hz), 2.73 (1H, dd, J = 14.0, 9.0 Hz), 2.48 (1H, td, J = 9.0, 4.5 Hz), 2.27 (1H, quind, J = 7.0, 1.5 Hz), 2.17 – 2.05 (2H, m), 1.86 (1H, dd, J = 14.5, 2.5 Hz), 1.82 – 1.73 (1H, m) overlaying 1.75 (1H, dd, J = 14.5, 4.5 Hz), 1.10 (3H, d, J = 7.0 Hz), 0.98 (3H, d, J = 7.0 Hz) overlaying 0.96 (9H, t, J = 8.0 Hz), 0.58 (6H, q, J = 8.0 Hz); ^{13}C NMR (101 MHz, $CDCl_3$) δ_C 205.3, 166.2, 139.6, 124.9, 94.5, 91.4, 77.7, 53.3, 52.0, 48.1, 43.5, 42.5, 42.0, 41.1, 16.6, 13.1, 7.0, 5.0; HRMS (ESI+) *m/z* $[M+Na]^+$ calcd for $C_{22}H_{36}NaO_5Si$, 431.2224; found, 431.2238.

Methyl 2-[(1R,2S*,3aR*,4R*,7S*,8aR*)-2-hydroxy-1,4-dimethyl-6-oxohexahydro-1H-3a,7-epoxyazulen-7(4H)-yl]acrylate (**S3**)*

A solution of silyl ether **29** (0.23 g, 0.56 mmol) in dry THF (10 mL) was cooled to 0 °C then TBAF (0.63 mL, 1.0 M in THF, 0.63 mmol) was added. The mixture was warmed to RT, stirred for 1 h, and saturated aqueous NH_4Cl solution (10 mL) was added then the mixture was extracted with ether (3 × 10 mL). The organic extracts were washed with brine (10 mL), dried over Na_2SO_4, and concentrated to give the alcohol, a colourless oil, which was taken directly into the next

step without purification. R_f 0.25 (pentane/ethyl acetate, 1:1); IR ν_{max}/cm^{-1} 3504w, 2921w, 1728s, 1203w, 1035m; ^{1}H NMR (600 MHz, $CDCl_3$) δ_H 6.27 (1H, d, *J* = 1.5 Hz), 5.97 (1H, d, *J* = 1.5 Hz), 4.29 (1H, ddd, *J* = 5.5, 4.0, 2.0 Hz), 3.73 (3H, s), 2.91 (1H, dd, *J* = 15.5, 7.0 Hz), 2.76 (1H, dd, *J* = 14.0, 9.0 Hz), 2.51 (1H, td, *J* = 9.5, 4.0 Hz), 2.33 (1H, quind, *J* = 7.0, 1.5 Hz), 2.21 (1H, dd, *J* = 15.0, 5.0 Hz), 2.13 (1H, dd, *J* = 15.5, 1.5 Hz), 1.94 (1H, dd, *J* = 15.0, 2.0 Hz), 1.89 – 1.83 (1H, m), 1.78 (1H, dd, *J* = 14.0, 4.0 Hz), 1.12 (3H, d, *J* = 7.0 Hz), 1.04 (3H, d, *J* = 7.0 Hz); ^{13}C NMR (151 MHz, $CDCl_3$) δ_C 204.7, 166.2, 139.5, 124.9, 94.8, 91.3, 77.7, 53.2, 51.9, 47.8, 42.9, 42.6, 41.9, 41.0, 16.5, 12.4; HRMS (ESI+) *m/z* $[M+Na]^+$ calcd for $C_{16}H_{22}NaO_5$, 317.1359; found, 317.1358.

Methyl 2-[(1R,3aR*,4R*,7S*,8aR*)-1,4-dimethyl-2,6-dioxohexahydro-1H-3a,7-epoxyazulen-7(4H)-yl]acrylate (**30**)*

To a solution of oxalyl chloride (96 µL, 1.1 mmol) in dry dichloromethane (10 mL) at –78 °C was added dimethyl sulfoxide (0.12 mL, 1.7 mmol). The solution was stirred for 15 min then a solution of the crude alcohol **S3** (from the previous experiment) in dry dichloromethane (2 mL) was added dropwise. The mixture was stirred at –78 °C for 15 min then triethylamine (0.48 mL, 3.4 mmol) was added; stirring was continued at –78 °C for 15 min then the mixture was warmed to RT, stirred for 2 h and saturated aqueous NH_4Cl solution (20 mL) added. The mixture was extracted with dichloromethane (3 × 20 mL), the combined organic extracts were washed with brine (2 × 20 mL), dried over Na_2SO_4, and concentrated. The residue was purified by chromatography (pentane/ethyl acetate, 1:1) to give the diketone **30** (0.15 g, 92% from **29**) as a colourless oil. R_f 0.32 (pentane/ethyl acetate, 1:1); IR ν_{max}/cm^{-1} 2923m, 2850w, 1745s, 1731s, 1459m; ^{1}H NMR (700 MHz, $CDCl_3$) δ_H 6.31 (1H, s), 6.00 (1H, s), 3.76 (3H, s), 3.06 (1H, dd, *J* = 15.0, 8.5 Hz), 3.00 (1H, dd, *J* = 15.5, 7.5 Hz), 2.82 (1H, d, *J* = 19.5 Hz), 2.48 (1H, td, *J* = 9.0, 3.0 Hz), 2.42 (1H, d, *J* = 19.5 Hz), 2.32 (1H, quind, *J* = 7.0, 1.0 Hz), 2.21 (1H, quind, *J* = 7.5, 1.5 Hz), 2.16 (1H, d, *J* = 15.5 Hz), 2.05 (1H, dd, *J* = 15.0, 2.5 Hz), 1.15 (3H, d, *J* = 7.0 Hz), 1.13 (3H, d, *J* = 7.0 Hz); ^{13}C NMR (176 MHz, $CDCl_3$) δ_C 216.5, 202.9, 165.9, 138.8, 125.1, 89.5, 88.9, 53.7, 52.2, 52.1, 46.3, 43.6, 42.5, 41.0, 16.8, 13.2; HRMS (ESI+) *m/z* $[M+H]^+$ calcd for $C_{16}H_{21}O_5$, 293.1384; found, 293.1382.

Methyl 2-[(1R,2S*,3aR*,7S*,8aR*)-2-hydroxy-1-methyl-6-oxo-2,3,8,8a-tetrahydro-1H-3a,7-epoxyazulen-7(6H)-yl]acrylate (**S4**)*

To a solution of silyl ether **27** (0.22 g, 0.56 mmol) in dry THF (10 mL) at 0 °C was added TBAF (0.63 mL, 1.0 M in THF, 0.63 mmol) and the mixture was warmed to RT then stirred for 1 h. Saturated aqueous NH_4Cl solution (10 mL)

was added and the mixture was extracted with ether (3 × 10 mL). The organic extracts were washed with brine (10 mL), dried over Na_2SO_4, and concentrated to give the alcohol, a colourless oil, which was taken directly into the next step without purification. R_f 0.15 (pentane/ethyl acetate, 1:1); IR ν_{max}/cm^{-1} 3504w, 3053w, 1731s, 1694s, 1439m; ^{1}H NMR (600 MHz, $CDCl_3$) δ_H 7.31 (1H, d, J = 9.5 Hz), 6.31 (1H, d, J = 1.0 Hz), 6.00 (1H, d, J = 9.5 Hz), 5.95 (1H, d, J = 1.0 Hz), 4.34 (1H, apparent t, J = 4.5 Hz), 3.78 (3H, s), 2.48 (1H, ddd, J = 11.0, 8.5, 2.5 Hz), 2.44 (1H, dd, J = 15.5, 5.5 Hz), 2.41 (1H, dd, J = 13.5, 8.5 Hz), 2.14 (1H, dd, J = 15.5, 1.0 Hz), 2.11 (1H, dd, J = 13.5, 2.5 Hz), 1.85 (1H, dqd, J = 11.0, 7.0, 4.0 Hz), 1.07 (3H, d, J = 7.0 Hz); ^{13}C NMR (151 MHz, $CDCl_3$) δ_C 195.4, 166.2, 156.5, 139.7, 126.1, 125.4, 91.4, 91.1, 78.7, 54.2, 52.1, 46.8, 44.6, 36.1, 12.1; HRMS (ESI+) *m/z* $[M+Na]^+$ calcd for $C_{15}H_{18}NaO_5$, 301.1046; found, 301.1055.

Methyl 2-[(1R,3aR*,7S*,8aR*)-1-methyl-2,6-dioxo-2,3,8,8a-tetrahydro-1H-3a,7-epoxyazulen-7(6H)-yl]acrylate (**32**)*

A solution of oxalyl chloride (97 μL, 1.11 mmol) in dichloromethane (10 mL) was cooled to –78 °C and dimethyl sulfoxide (0.12 mL, 1.7 mmol) was added. The solution was stirred for 15 min then a solution of the crude alcohol **S4** (from the previous experiment) in dichloromethane (2 mL) was added. The mixture was stirred at –78 °C for 15 min then triethylamine (0.48 mL, 3.4 mmol) was added, stirring continued at –78 °C for 15 min then warmed to RT and stirred for 2 h. Saturated aqueous NH_4Cl solution (20 mL) was added, the mixture extracted with dichloromethane (3 × 20 mL), and the organic extracts were washed with brine (2 × 20 mL), dried over Na_2SO_4, and concentrated. The residue was purified by chromatography (pentane/ethyl acetate, 1:1) to give diketone **32** (0.12 g, 78% from **27**) as a colourless oil. R_f 0.40 (pentane/ethyl acetate, 1:1); IR ν_{max}/cm^{-1} 2954w, 1747s, 1727s, 1697s, 1073s; ^{1}H NMR (400 MHz, $CDCl_3$) δ_H 7.15 (1H, d, J = 10.0 Hz), 6.32 (1H, d, J = 1.0 Hz), 6.09 (1H, d, J = 10.0 Hz), 5.96 (1H, d, J = 1.0 Hz), 3.80 (3H, s), 3.00 (1H, dd, J = 19.5, 1.0 Hz), 2.73 (1H, dd, J = 14.5, 8.0 Hz), 2.63 (1H, d, J = 19.5 Hz), 2.45 – 2.29 (2H, m), 2.16 (1H, dqd, J = 10.5, 7.0, 1.0 Hz), 1.16 (3H, d, J = 7.0 Hz); ^{13}C NMR (101 MHz, $CDCl_3$) δ_C 215.5, 194.1, 166.0, 152.3, 138.9, 127.2, 125.2, 89.6, 85.4, 52.2, 51.6, 51.0, 46.3, 38.7, 13.0; HRMS (ESI+) *m/z* $[M+H]^+$ calcd for $C_{15}H_{17}O_5$, 277.1071; found, 277.1073.

Methyl 2-{(1R,3aR*,7S*,8aR*)-1-methyl-6-oxo-2-[(triethylsilyl)oxy]-8,8a-dihydro-1H-3a,7-epoxyazulen-7(6H)-yl}acrylate (**S5**)*

Chlorotriethylsilane (0.60 mL, 3.6 mmol) was added to a solution of diketone **32** (0.493 g, 1.78 mmol) in THF (18 mL) then the solution was cooled to –78 °C. KHMDS (2.1 mL, 1.0 M in THF, 2.1 mmol) was added, stirring continued at –78 °C for 1 h then saturated aqueous $NaHCO_3$ solution (10 mL) was added dropwise. The mixture was warmed to RT, extracted with ether (3 × 10 mL), the organic extracts washed with brine (10 mL), dried over Na_2SO_4, and concentrated. The residue was purified by chromatography (pentane/ethyl acetate, 10:1) to give silyl enol ether **S5** (0.63 g, 91%) as a colourless oil. R_f 0.35 (pentane/ethyl acetate, 10:1); IR ν_{max}/cm^{-1} 2956m, 1735w, 1700w, 1073s, 740s; ^1H NMR (400 MHz, C_6D_6) δ_H 6.69 (1H, d, *J* = 9.5 Hz), 6.27 (1H, d, *J* = 1.5 Hz), 5.95 (1H, d, *J* = 1.5 Hz), 5.87 (1H, d, *J* = 9.5 Hz), 4.79 (1H, d, *J* = 2.0 Hz), 3.45 (3H, s), 2.53 (1H, dd, *J* = 14.0, 9.0 Hz), 2.40 (1H, quind, *J* = 7.0, 2.0 Hz), 2.13 (1H, dd, *J* = 14.0, 4.0 Hz), 1.96 (1H, ddd, *J* = 9.0, 6.5, 3.5 Hz), 0.97 (3H, d, *J* = 7.0 Hz), 0.93 (9H, t, *J* = 8.0 Hz), 0.60 (6H, q, *J* = 8.0 Hz); ^{13}C NMR (101 MHz, C_6D_6) δ_C 194.9, 166.1, 165.3, 155.9, 141.1, 126.9, 124.0, 100.4, 91.8, 90.6, 55.3, 51.5, 47.2, 37.8, 17.3, 6.7, 5.0; HRMS (ESI+) *m/z* $[M+H]^+$ calcd for $C_{21}H_{31}O_5Si$, 391.1935; found, 391.1939.

Methyl 2-{(1R,3aR*,4R*,7S*,8aR*)-1,4-dimethyl-6-oxo-2-[(triethylsilyl)oxy]-5,6,8,8a-tetrahydro-1H-3a,7-epoxyazulen-7(4H)-yl}acrylate (**33**)*

A suspension of CuI (0.31 g, 1.63 mmol) in THF (20 mL) was cooled to 0 °C and methyllithium (2.0 mL, 1.6 M in ether, 3.2 mmol) added dropwise. The solution was stirred for 30 min then a solution of silyl enol ether **S5** (0.633 g, 1.62 mmol) in THF (5 mL) was added. The mixture was stirred at 0 °C for 1 h then saturated aqueous NH_4Cl solution (20 mL) was added; the mixture was extracted with ether (3 × 20 mL), the organic extracts were washed with brine (20 mL) then dried over Na_2SO_4 and concentrated. The residue was purified by chromatography (pentane/ethyl acetate, 3:1) to give the title compound (0.398 g, 60%) as a yellow oil. R_f 0.71 (pentane/ethyl acetate, 3:1); IR ν_{max}/cm^{-1} 2981s, 2887m, 1732m, 1684w, 1005m; ^1H NMR (400 MHz, C_6D_6) δ_H 6.28 (1H, d, *J* = 2.0 Hz), 6.05 (1H, d, *J* = 2.0 Hz), 4.91 (1H, d, *J* = 2.0 Hz), 3.38 (3H, s), 2.87 (1H, dd, *J* = 13.5, 9.0 Hz), 2.50 (1H, dd, *J* = 15.5, 7.5 Hz), 2.45 – 2.33 (1H, m), 2.15 (1H, dd, *J* = 15.5, 2.0 Hz), 1.94 (1H, dt, *J* = 9.0, 5.5 Hz), 1.82 (1H, dd, *J* = 13.5, 6.0 Hz), 1.75 (1H, quind, *J* = 7.0, 2.0 Hz), 1.11 (3H, d, *J* = 7.0 Hz), 1.08 (3H, d, *J* = 7.0 Hz), 0.95 (9H, t, *J* = 8.0 Hz), 0.63 (6H, q, *J* = 8.0 Hz); ^{13}C NMR (101 MHz, C_6D_6) δ_C 204.3, 166.0, 162.9, 140.8, 124.2, 100.5, 95.1, 90.6, 53.3, 51.4, 49.0, 42.7, 42.3, 42.3, 18.6, 17.2, 6.8, 5.1; HRMS (ESI+) *m/z* $[M+H]^+$ calcd for $C_{22}H_{35}O_5Si$, 407.2248; found, 407.2253.

Methyl 2-[(3R,3aR*,5S*,8R*)-5-acetoxy-3,8-dimethyl-2,6-dioxo-2,3,3a,4,5,6,7,8-octahydroazulen-5-yl]acrylate* ***(34)***

$ZnCl_2$ (0.50 g, 3.7 mmol) and acetic anhydride (50 µL, 0.53 mmol) were added to a solution of silyl enol ether **33** (10 mg, 0.025 mmol) in dry benzene (1 mL). The mixture was stirred at RT for 1 h, triethylamine (50 µL, 0.36 mmol) was added, and stirring continued for another 1 h. Ether (20 mL) was added then the mixture was washed successively with water (10 mL) and brine (10 mL), dried over Na_2SO, and concentrated. The residue was purified by chromatography (pentane/ethyl acetate, 2:1) to give enone **34** (6.0 mg, 72%) as a colourless oil. R_f 0.15 (pentane/ethyl acetate, 2:1); IR ν_{max}/cm^{-1} 2926w, 2850w, 1730s, 1711s, 1244m; ^1H NMR (600 MHz, $CDCl_3$) δ_H 6.49 (1H, s), 5.96 (1H, s), 5.90 (1H, s), 3.80 (3H, s), 3.05 (1H, t, J = 13.0 Hz), 2.93 – 2.86 (1H, m), 2.73 – 2.66 (2H, m), 2.63 – 2.54 (2H, m), 2.11 (1H, dtd, J = 10.0, 7.0, 3.0 Hz), 2.05 (3H, s), 1.33 (3H, d, J = 6.5 Hz), 1.18 (3H, d, J = 7.5 Hz); ^{13}C NMR (151 MHz, $CDCl_3$) δ_C 209.5, 204.6, 183.5, 169.7, 166.1, 139.8, 129.0, 127.4, 86.1, 52.6, 49.7, 48.4, 47.2, 38.8, 33.4, 21.3, 20.2, 14.6; HRMS (ESI+) *m/z* $[M+H]^+$ calcd for $C_{18}H_{23}O_6$, 335.1489; found, 335.1490.

3. For Scheme 4

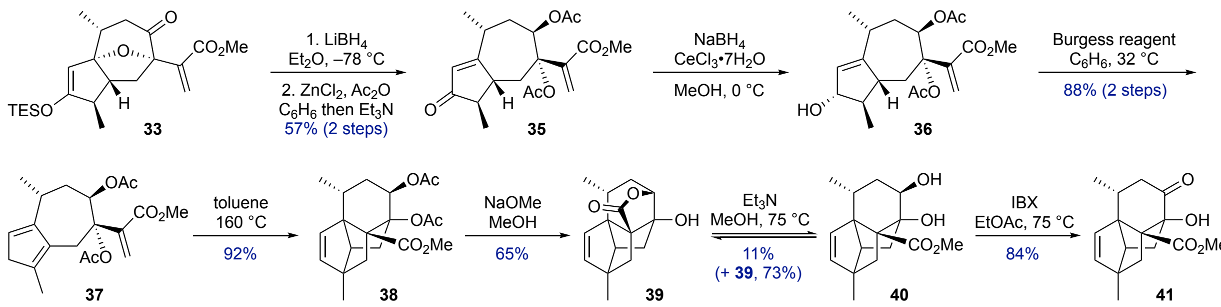

Methyl 2-{(1R,3aR*,4R*,6S*,7S*,8aR*)-6-hydroxy-1,4-dimethyl-2-[(triethylsilyl)oxy]-5,6,8,8a-tetrahydro-1H-3a,7-epoxyazulen-7(4H)-yl}acrylate* ***(S6)***

$LiBH_4$ solution (81 µL, 2.0 M in THF, 0.16 mmol) was added dropwise to a solution of ketone **33** (22 mg, 0.054 mmol) in ether at –78 °C. Stirring was continued at –78 °C for 0.5 h, saturated aqueous NH_4Cl solution (10 mL) was added dropwise then the mixture was warmed to RT and extracted with ether (3 × 10 mL). The organic extracts were washed with brine (20 mL), dried over Na_2SO_4, and concentrated to obtain alcohol **S6** as a yellow oil that was taken directly into the next reaction. R_f 0.11 (pentane/ethyl acetate, 10:1); IR ν_{max}/cm^{-1} 2956m, 1633s, 1009s, 977s; ^1H NMR (400 MHz, C_6D_6) δ_H 6.17 (1H, d, J = 2.0 Hz), 6.07 (1H, d, J = 2.0 Hz), 4.97 (1H, d, J = 2.0 Hz), 4.00 – 3.93 (1H, m), 3.57 (1H, s), 3.26 (3H, s), 2.86 (1H, dd, J = 13.0, 9.5 Hz), 2.42 (1H, quind, J = 7.0, 2.0 Hz), 1.94 (1H, ddd, J = 9.0, 6.0, 4.5 Hz), 1.85

(1H, ddd, J = 13.5, 6.0, 1.5 Hz), 1.81 – 1.72 (1H, m), 1.64 – 1.53 (2H, m), 1.23 (3H, d, J = 7.0 Hz), 1.07 (3H, d, J = 7.0 Hz), 0.95 (9H, t, J = 8.0 Hz), 0.63 (6H, q, J = 8.0 Hz); ^{13}C NMR (101 MHz, C_6D_6) δ_C 169.3, 162.8, 145.2, 125.3, 101.3, 94.4, 88.3, 71.8, 54.3, 51.8, 49.4, 38.5, 36.7, 36.0, 18.2, 16.6, 6.8, 5.1; HRMS (ESI+) *m/z* $[M+H]^+$ calcd for $C_{22}H_{37}O_5Si$, 409.2405; found, 409.2415.

(3R,3aR*,5S*,6R*,8R*)-5-(3-Methoxy-3-oxoprop-1-en-2-yl)-3,8-dimethyl-2-oxo-2,3,3a,4,5,6,7,8-octahydroazulene-5,6-diyl diacetate (**35**)*

$ZnCl_2$ (0.40 g, 2.9 mmol) and acetic anhydride (0.10 mL, 1.1 mmol) were added to a solution of crude alcohol **S6** (9.5 mg, ~0.023 mmol) in dry benzene (1 mL). The mixture was stirred at RT for 1 h, triethylamine (0.10 mL, 0.72 mmol) was added and stirring continued for another 1 h. Ether (20 mL) was added then the mixture was washed with water (10 mL) and brine (10 mL) then dried over Na_2SO_4 and concentrated. The residue was purified by chromatography (pentane/ethyl acetate, 3:1) to give the title compound (5.0 mg, 57%) as a colourless oil. R_f 0.25 (pentane/ethyl acetate, 1:1); IR ν_{max}/cm^{-1} 2981s, 2889m, 1744m, 1381m; ^{1}H NMR (500 MHz, C_6D_6) δ_H 6.18 (1H, s), 5.84 (1H, s), 5.82 (1H, dd, J = 4.5, 2.5 Hz), 5.36 (1H, s), 3.30 (3H, s), 2.47 (1H, dd, J = 14.5, 7.0 Hz), 2.38 – 2.22 (4H, m), 1.81 (1H, ddd, J = 15.0, 5.0, 3.0 Hz), 1.72 – 1.64 (1H, m) overlaying 1.67 (3H, s), 1.61 (3H, s), 1.13 (3H, d, J = 7.0 Hz), 0.84 (3H, d, J = 7.0 Hz); ^{13}C NMR (126 MHz, C_6D_6) δ_C 208.4, 184.5, 169.8, 168.5, 165.1, 142.7, 125.5, 125.0, 82.1, 71.8, 51.6, 48.9, 48.8, 37.4, 31.1, 30.8, 21.3, 20.5, 19.7, 15.0; HRMS (ESI+) *m/z* $[M+H]^+$ calcd for $C_{20}H_{27}O_7$, 379.1751; found, 379.1744.

(2S,3R*,3aR*,5S*,6R*,8R*)-2-Hydroxy-5-(3-methoxy-3-oxoprop-1-en-2-yl)-3,8-dimethyl-2,3,3a,4,5,6,7,8-octahydroazulene-5,6-diyl diacetate (**36**)*

$CeCl_3 \cdot 7H_2O$ (8.5 mg, 0.023 mmol) then $NaBH_4$ (1.0 mg, 0.026 mmol) were added to a solution of enone **35** (8.5 mg, 0.022 mmol) in methanol (1 mL) at 0 °C. The mixture was stirred at 0 °C for 1 h then water (5 mL) was added and the mixture was extracted with ether (3 × 5 mL). The combined organic extracts were washed with brine (10 mL), dried over $MgSO_4$, and concentrated. The crude product **36**, a colourless oil, was used directly in the next step without purification. R_f 0.25 (pentane/ethyl acetate, 1:1); IR ν_{max}/cm^{-1} 3465w, 2981s, 1740s, 1380m, 1237m; ^{1}H NMR (700 MHz, C_6D_6) δ_H 6.23 (1H, s), 5.91 (1H, dd, J = 5.0, 2.5 Hz), 5.49 (1H, s), 5.30 (1H, q, J = 2.0 Hz), 4.06 (1H, dq, J = 6.0, 2.0 Hz), 3.33 (3H, s), 2.61 (1H, dd, J = 15.0, 6.5 Hz), 2.35 – 2.26 (1H, m) overlaying 2.29 (1H, dd, J = 15.0, 4.0 Hz), 2.22 – 2.18 (1H, m), 1.99 – 1.93 (2H, m), 1.92 (3H, s), 1.80 – 1.75 (1H, m), 1.61 (3H, s), 1.07 (3H, d, J = 7.0 Hz), 1.02 (3H, d, J = 7.0

Hz); ^{13}C NMR (176 MHz, C_6D_6) δ_C 169.8, 168.7, 165.5, 153.3, 143.3, 124.9, 124.8, 82.8, 82.3, 72.6, 51.5, 50.6, 50.4, 37.5, 33.5, 28.9, 21.9, 20.5, 20.5, 17.1; HRMS (ESI+) *m/z* $[M+Na]^+$ calcd for $C_{20}H_{28}NaO_7$, 403.1727; found, 403.1727.

(5S,6R*,8R*)-5-(3-Methoxy-3-oxoprop-1-en-2-yl)-3,8-dimethyl-2,4,5,6,7,8-hexahydroazulene-5,6-diyl diacetate (**37**)*

Burgess reagent (10.5 mg, 0.0441 mmol) was added to a solution of the crude alcohol **36** from the previous reaction in benzene (1 mL) at RT. The resulting solution was warmed to 32 °C and stirred for 2 h. After cooling to RT the reaction was quenched with water (10 mL); the aqueous phase was extracted with ether (3 × 10 mL) and the combined organic extracts were washed with brine (10 mL), dried over Na_2SO_4, and concentrated. The residue was purified by chromatography (pentane/ethyl acetate, 10:1) to give the diene **37** (7.0 mg, 88% from **35**) as a colourless oil. R_f 0.80 (pentane/ethyl acetate, 2:1); IR ν_{max}/cm^{-1} 3232w, 3096m, 1746s, 1016m; ^{1}H NMR (500 MHz, C_6D_6) δ_H 6.25 (1H, s), 5.97 – 5.92 (1H, m), 5.71 (1H, apparent d, *J* = 2.0 Hz), 5.48 (1H, s), 3.57 (1H, dd, *J* = 14.0, 1.5 Hz), 3.34 (3H, s), 3.01 – 2.94 (1H, m), 2.85 – 2.78 (1H, m), 2.68 – 2.65 (2H, m), 2.05 (1H, ddd, *J* = 15.0, 11.5, 2.0 Hz), 1.93 (3H, d, *J* = 1.5 Hz), 1.89 (1H, dd, *J* = 15.0, 5.0 Hz), 1.62 (3H, s), 1.61 (3H, s), 1.14 (3H, d, *J* = 7.0 Hz); ^{13}C NMR (126 MHz, C_6D_6) δ_C 168.8, 168.7, 165.1, 153.5, 143.7, 140.3, 135.4, 125.1, 119.8, 81.5, 72.3, 51.5, 44.4, 37.4, 29.8, 27.2, 20.8, 20.6, 20.0, 13.6; HRMS (ESI+) *m/z* $[M+Na]^+$ calcd. for $C_{20}H_{26}NaO_6$, 385.1622; found, 385.1627.

(1S,3aR*,4R*,6R*,7R*,8aS*,9S*)-9-(Methoxycarbonyl)-1,4-dimethyl-5,6,8,8a-tetrahydro-1H-3a,7,1-(epiethane[1,1,2]triyl)azulene-6,7(4H)-diyl diacetate (**38**)*

A solution of cyclopentadiene derivative **37** (6.5 mg, 0.018 mmol) in toluene (1 mL) was heated to 160 °C in a pressure tube for 3 h. The solution was cooled to RT, concentrated, then the residue was purified by chromatography (pentane/ethyl acetate, 6:1) to give the cycloadduct **38** (6.0 mg, 92%) as a colourless oil. R_f 0.11 (pentane/ethyl, 6:1); IR ν_{max}/cm^{-1} 3115w, 2922m, 1745s, 1435m, 1006m; ^{1}H NMR (700 MHz, C_6D_6) δ_H 6.12 – 6.09 (1H, m), 5.91 (1H, d, *J* = 5.5 Hz), 5.81 (1H, d, *J* = 5.5 Hz), 3.36 (3H, s), 3.02 (1H, apparent sept, *J* = 6.5 Hz), 2.19 (1H, dd, *J* = 15.0, 5.5 Hz), 2.15 (1H, d, *J* = 12.0 Hz), 1.92 (3H, s), 1.72 (3H, s), 1.64 – 1.55 (2H, m) overlaying 1.59 (1H, d, *J* = 14.5 Hz), 1.35 (1H, ddd, *J* = 15.5, 12.5, 4.0 Hz), 1.15 (1H, d, *J* = 12.0 Hz), 0.87 (3H, s), 0.80 (3H, d, *J* = 7.0 Hz); ^{13}C NMR (176 MHz, C_6D_6) δ_C 172.7, 170.1, 169.4, 141.1, 133.1, 90.8, 71.6, 66.0, 58.4, 57.6, 52.2, 50.8, 43.6, 34.9, 32.2, 25.6, 21.7, 20.7, 18.8, 15.0; HRMS (ESI+) *m/z* $[M+NH_4]^+$ calcd for $C_{20}H_{30}NO_6$, 380.2068; found, 380.2071.

*(1R,3aS,3bR,6S,6aS,7aS,9R)-7a-Hydroxy-6,9-dimethyl-6,6a,7,7a-tetrahydro-1H,3H-3b,1-ethano-3a,6-methanopentaleno[1,2-c]furan-3-one (**39**)*

Sodium methoxide solution (0.2 mL, 1.0 M in methanol, 0.2 mmol) was added to a solution of cycloadduct **38** (18 mg, 0.050 mmol) in methanol (0.5 mL). The solution was stirred at RT for 4 h then quenched by adding Amberlyst® 15 (18 mg). The resulting mixture was filtered through a pad of Celite®, washing through with methanol (3 × 5 mL). The combined filtrate was concentrated and purified by chromatography (pentane/ethyl acetate, 1:1) to give lactone **39** (8.0 mg, 65%) as a colourless oil. R_f 0.45 (pentane/ethyl acetate, 1:1); IR ν_{max}/cm^{-1} 3444w, 2956m, 1770sh, 1755s, 1647s, 1053m; ^{1}H NMR (700 MHz, C_6D_6) δ_H 5.96 (1H, d, *J* = 5.5 Hz), 5.36 (1H, d, *J* = 5.5 Hz), 4.20 (1H, dd, *J* = 4.0, 1.5 Hz), 2.30 (1H, br s), 2.03 (1H, ddq, *J* = 11.0, 7.5, 7.0 Hz), 1.93 (1H, d, *J* = 12.0 Hz), 1.75 (1H, ddd, *J* = 14.5, 7.5, 4.0 Hz), 1.48 (1H, d, *J* = 12.0 Hz), 1.47 (1H, d, *J* =), 1.36 (1H, ddd, *J* = 14.5, 4.5, 1.0 Hz), 1.10 (1H, d, *J* = 14.5 Hz), 0.95 (1H, ddd, *J* = 14.5, 11.0, 1.5 Hz) overlaying 0.93 (3H, s), 0.64 (3H, d, *J* = 7.0 Hz); ^{13}C NMR (176 MHz, C_6D_6) δ_C 175.4, 145.4, 130.8, 89.0, 85.2, 72.1, 63.2, 60.0, 55.1, 34.8, 33.5, 30.1, 27.5, 18.1, 15.9; HRMS (ESI+) *m/z* $[M+Na]^+$ calcd for $C_{15}H_{18}NaO_3$, 269.1151; found, 269.1148.

Methyl (1S,3aR,4R,6R,7S,8aS,9S)-6,7-dihydroxy-1,4-dimethyl-4,5,6,7,8,8a-hexahydro-1H-3a,7,1-(epiethane[1,1,2]-triyl)azulene-9-carboxylate (**40**)

Triethylamine (30 μL, 0.22 mmol) was added to a solution of lactone **39** (8.0 mg, 0.032 mmol) in methanol (0.5 mL). The resulting solution was stirred at 80 °C (oil bath temperature; sealed sample vial) for 18 h then cooled to RT and concentrated. The residue was purified by chromatography (pentane/ethyl acetate, 4:1 → 2:1) to give the diol **40** as a colourless oil [1.0 mg, 11%; 73% based on recovered **39** (6.8 mg, 85%) which was recycled]. R_f 0.33 (pentane/ethyl acetate, 1:1); IR ν_{max}/cm^{-1} 3489w, 2953s, 2927s, 1737s, 1297s; ^{1}H NMR (600 MHz, C_6D_6) δ_H 5.92 (1H, d, *J* = 5.5 Hz), 5.86 (1H, d, *J* = 5.5 Hz), 4.02 (1H, s), 3.65 (1H, br dd, *J* = 4.0, 2.0 Hz), 3.32 (3H, s), 2.88 (1H, apparent sept, *J* = 6.5 Hz), 2.41 (1H, d, *J* = 11.5 Hz), 1.61 – 1.53 (2H, m), 1.37 – 1.35 (1H, m), 1.28 – 1.21 (2H, m), 1.10 (1H, dd, *J* = 11.5, 1.5 Hz), 0.95 (3H, s), 0.87 (3H, d, *J* = 7.0 Hz); ^{13}C NMR (151 MHz, C_6D_6) δ_C 175.3, 141.3, 133.0, 85.4, 75.9, 66.7, 58.8, 56.4, 52.5, 51.0, 43.5, 37.2, 31.4, 24.9, 19.2, 15.2; HRMS (ESI+) *m/z* $[M+H]^+$ calcd for $C_{16}H_{23}O_4$, 279.1591; found, 279.1592.

*Methyl (1S,3aR,4R,7S,8aS,9S)-7-hydroxy-1,4-dimethyl-6-oxo-4,5,6,7,8,8a-hexahydro-1H-3a,7,1-(epiethane[1,1,2]-triyl)azulene-9-carboxylate (**41**)*

A solution of IBX (10 mg, 0.036 mmol) and diol **40** (2.5 mg, 8.6 μmol) in ethyl acetate (0.2 mL) was stirred at 80 °C (oil bath temperature; sealed sample vial) for 2.5 h. The mixture was filtered through a pad of Celite®, washing through with ethyl acetate. The filtrate was concentrated to give keto-alcohol **41** as a colourless oil (2.0 mg, 84%). R_f 0.42 (pentane/ethyl acetate, 1:1); IR ν_{max}/cm^{-1} 3477w, 2926w, 2851w, 1725s, 1301m; ^1H NMR (500 MHz, C_6D_6) δ_H 5.94 (1H, d, *J* = 5.5 Hz), 5.42 (1H, d, *J* = 5.5 Hz), 4.55 (1H, s), 3.20 (3H, s), 2.62 (1H, dd, *J* = 17.0, 7.5 Hz), 2.41 (1H, d, *J* = 12.0 Hz), 2.31 (1H, dquin, *J* = 10.5, 7.0 Hz), 1.88 (1H, ddd, *J* = 17.0, 11.0, 1.5 Hz), 1.81 (1H, dd, *J* = 12.0, 1.5 Hz), 1.67 (1H, dd, *J* = 7.0, 1.5 Hz), 1.42 (1H, d, *J* = 14.0 Hz), 1.24 (1H, dd, *J* = 14.0, 5.5 Hz), 0.93 (3H, s), 0.71 (3H, d, *J* = 7.0 Hz); ^{13}C NMR (126 MHz, C_6D_6) δ_C 208.6, 172.4, 143.8, 130.8, 88.0, 69.7, 63.2, 61.5, 54.0, 51.4, 41.4, 39.8, 33.0, 30.0, 19.3, 15.1; HRMS (ESI+) *m/z* $[M+H]^+$ calcd for $C_{16}H_{21}O_4$, 277.1434; found, 277.1431.

*Methyl (1S,3aR,7S,8aS,9S)-7-hydroxy-1,2,4-trimethyl-6-oxo-2,3,6,7,8,8a-hexahydro-1H-3a,7,1-(epiethane[1,1,2]-triyl)azulene-9-carboxylate (**42**)*

This compound, whose assignment is tentative, was isolated from an attempt to effect Saegusa oxidation on the trimethylsilyl enol ether derivative of compound **41**. Despite the use of excess base and silylating agent in the first step, the product isolated was the silyl ether of the 3°-alcohol and this was taken directly into the Saegusa-variant oxidation conditions. Thus, a solution of chlorotrimethyl silane (10 μL, 0.079 mmol) and keto-alcohol **41** (1.5 mg, 5.4 μmol) in THF (1 mL) was cooled to –78 °C and LHMDS (16 μL, 1.0 M in THF, 0.016 mmol) was added. The mixture was stirred at –78 °C for 0.5 h then at RT for another 0.5 h. Saturated aqueous $NaHCO_3$ solution (5 mL) was added at 0 °C and the mixture was extracted with ether (3 × 5 mL); the organic extracts were washed with brine (5 mL) then dried over Na_2SO_4 and concentrated. The resulting TMS ether, a colourless oil, was used directly in the next step without purification. $Pd(OAc)_2$ (2.5 mg, 11 μmol) and DMSO (1.5 μL, 21 μmol) were added to a solution of this silyl ether in acetic acid (0.2 mL) under oxygen atmosphere (balloon). The mixture was heated to 80 °C and stirred vigorously for 18 h. The mixture was cooled to RT and concentrated, then the residue was purified by chromatography (pentane/ethyl acetate, 2:1) to give the product, assigned as enone **42**, as a colourless oil (1.0 mg, 64% from **41**). R_f 0.39 (pentane/ethyl acetate, 2:1); IR ν_{max}/cm^{-1} 2956m, 2918m, 1729s, 1682s, 1299m; ^1H NMR (600 MHz, $CDCl_3$) δ_H 6.07 (1H, q, *J* = 1.5 Hz), 3.59 (3H, s), 2.44 (1H, dd, *J* = 14.0, 8.5 Hz), 2.06 (1H, d, *J* = 13.0 Hz), 1.99 (3H, d, *J* = 1.5 Hz), 1.97 (1H, d, *J* = 6.0 Hz), 1.88 – 1.82

(1H, m), 1.81 – 1.76 (1H, m), 1.72 (1H, dd, J = 14.0, 6.0 Hz), 1.57 – 1.55 (2H, m occluded by solvent impurity), 1.10 (3H, s), 0.94 (3H, d, J = 7.0 Hz); ^{13}C NMR (151 MHz, $CDCl_3$) δ_C 199.5, 172.0, 162.6, 124.3, 84.9, 69.6, 64.1, 52.0, 51.8, 51.3, 43.4, 39.9, 33.7, 32.7, 23.2, 19.2, 16.0; HRMS (ESI+) *m/z* $[M+Na]^+$ calcd for $C_{17}H_{22}NaO_4$, 313.1410; found, 313.1403.

^{1}H NMR (500 MHz, $CDCl_3$) (S1)

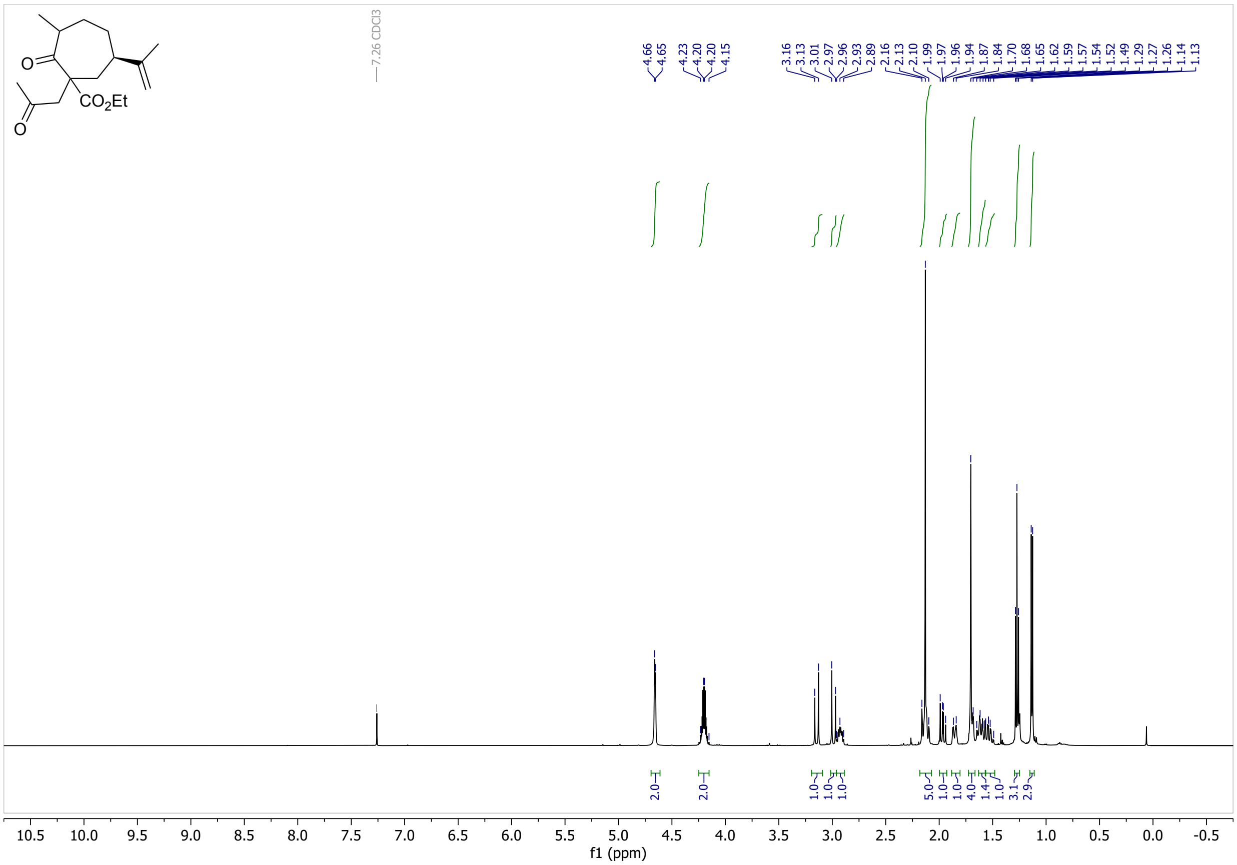

^{13}C NMR (126 MHz, $CDCl_3$) (S1)

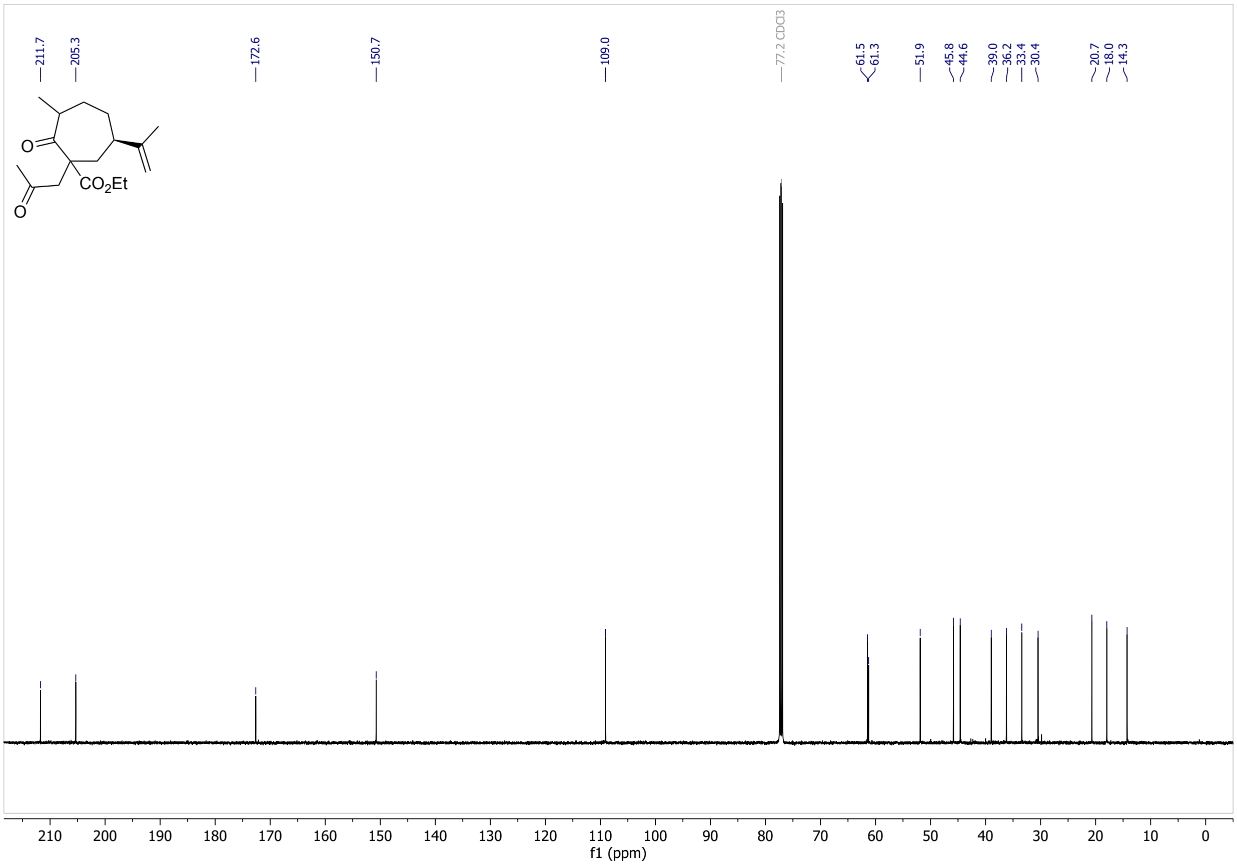

^{1}H NMR (400 MHz, CDCl$_3$) **(12)** – equilibrating isomeric mixture

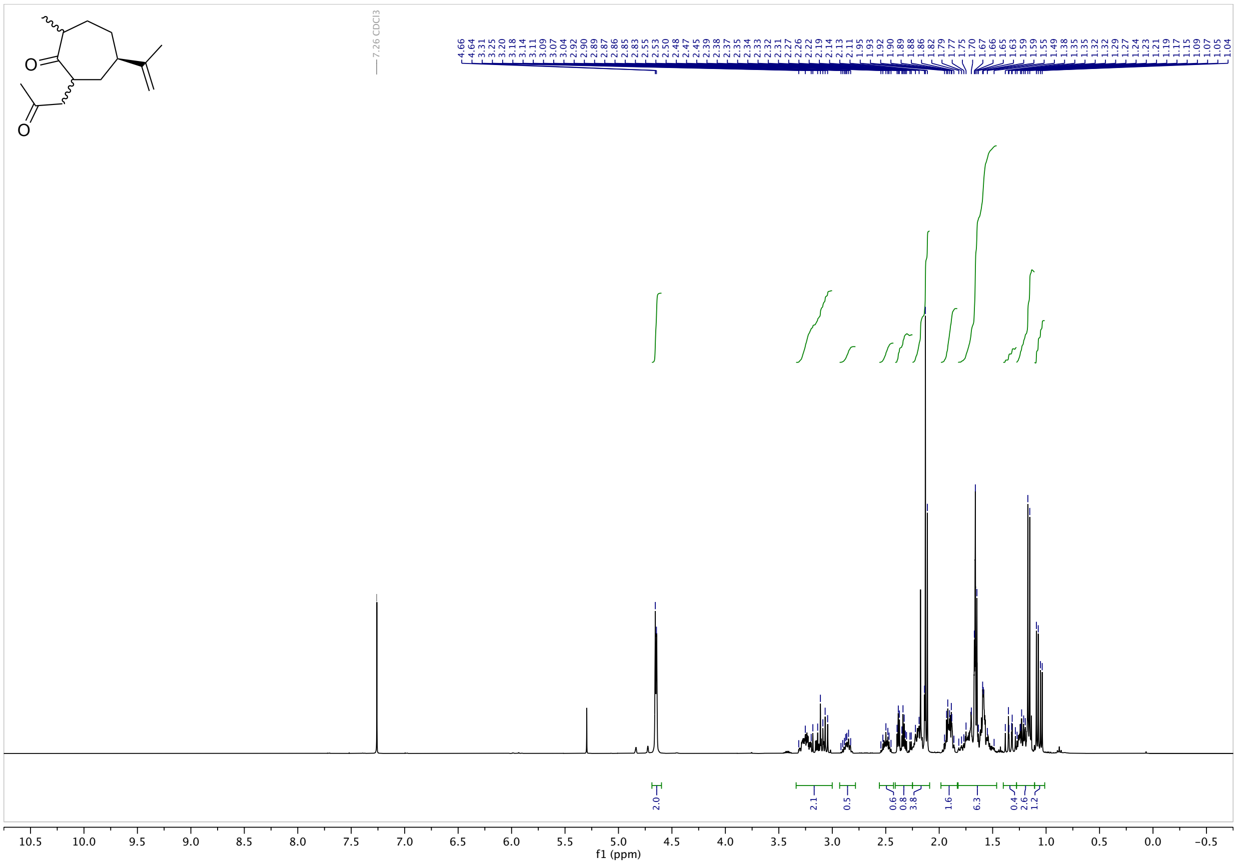

^{1}H NMR (400 MHz, CDCl$_3$) **(13)** – equilibrating isomeric mixture

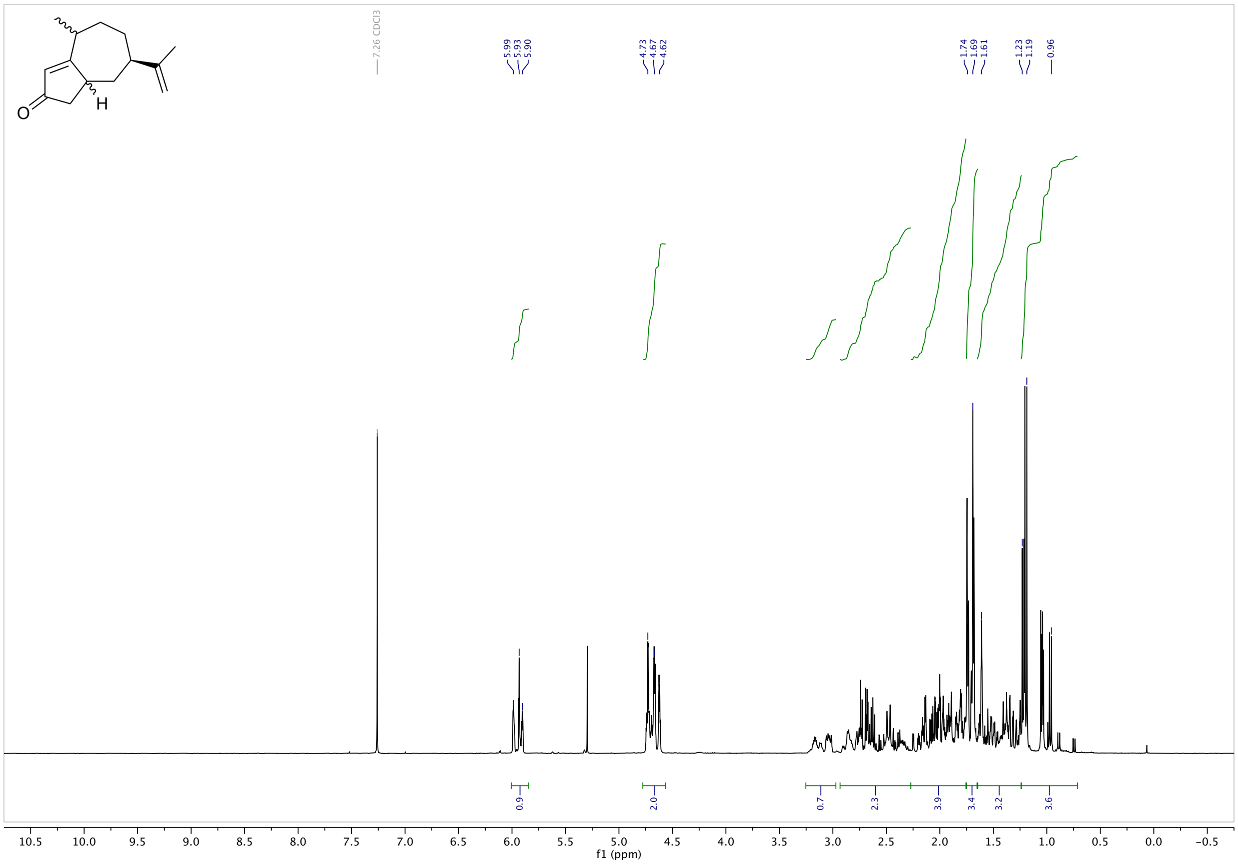

^{1}H NMR (600 MHz, CDCl$_3$) **(15)** – crude

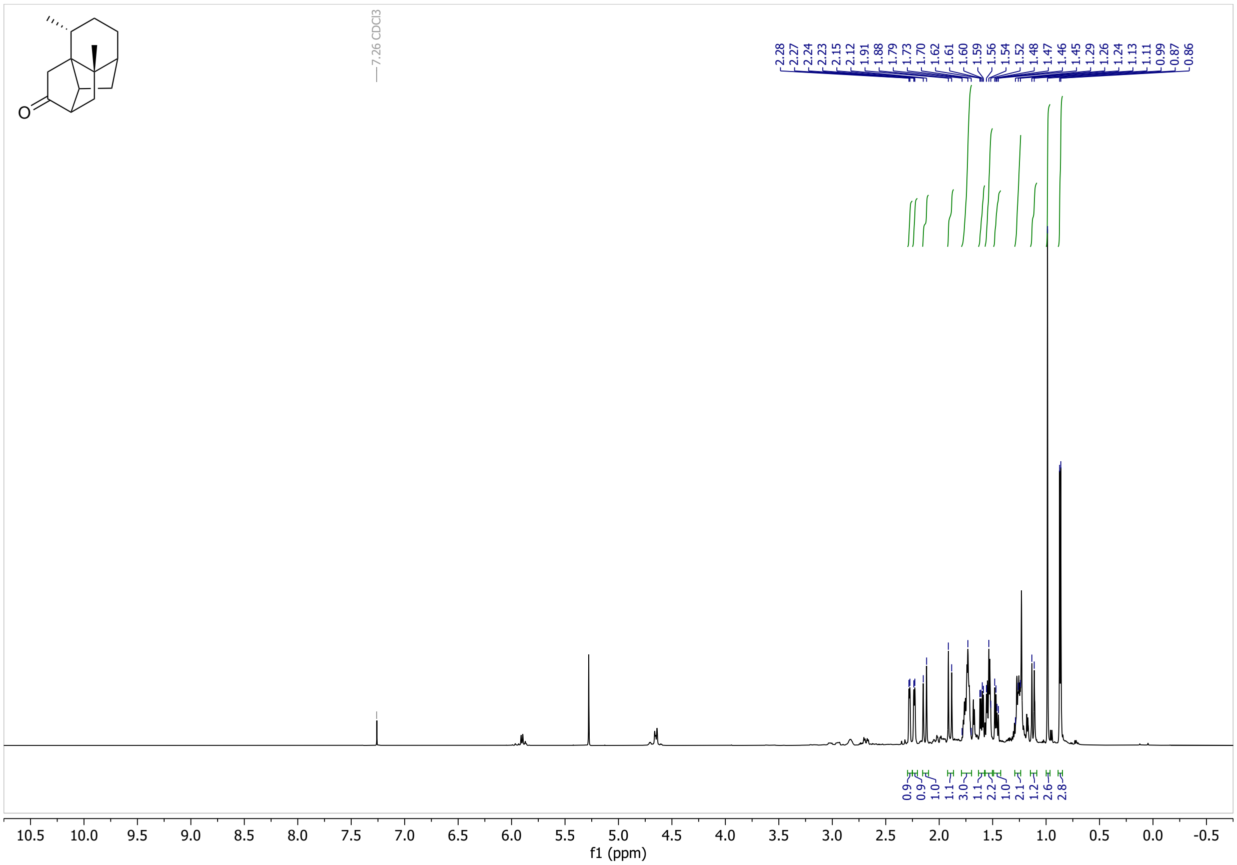

^{13}C NMR (151 MHz, CDCl$_3$) **(15)** – crude

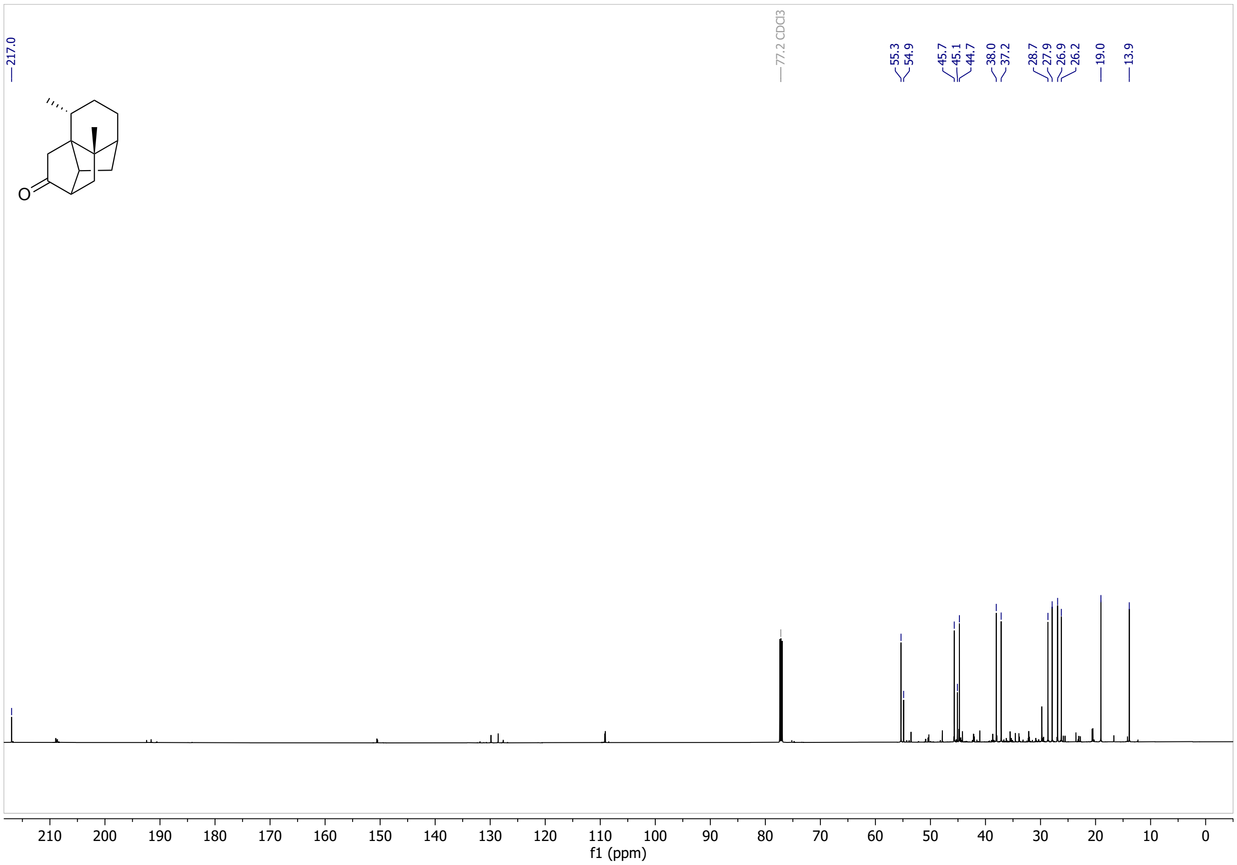

^{1}H NMR (500 MHz, $CDCl_3$) (16)

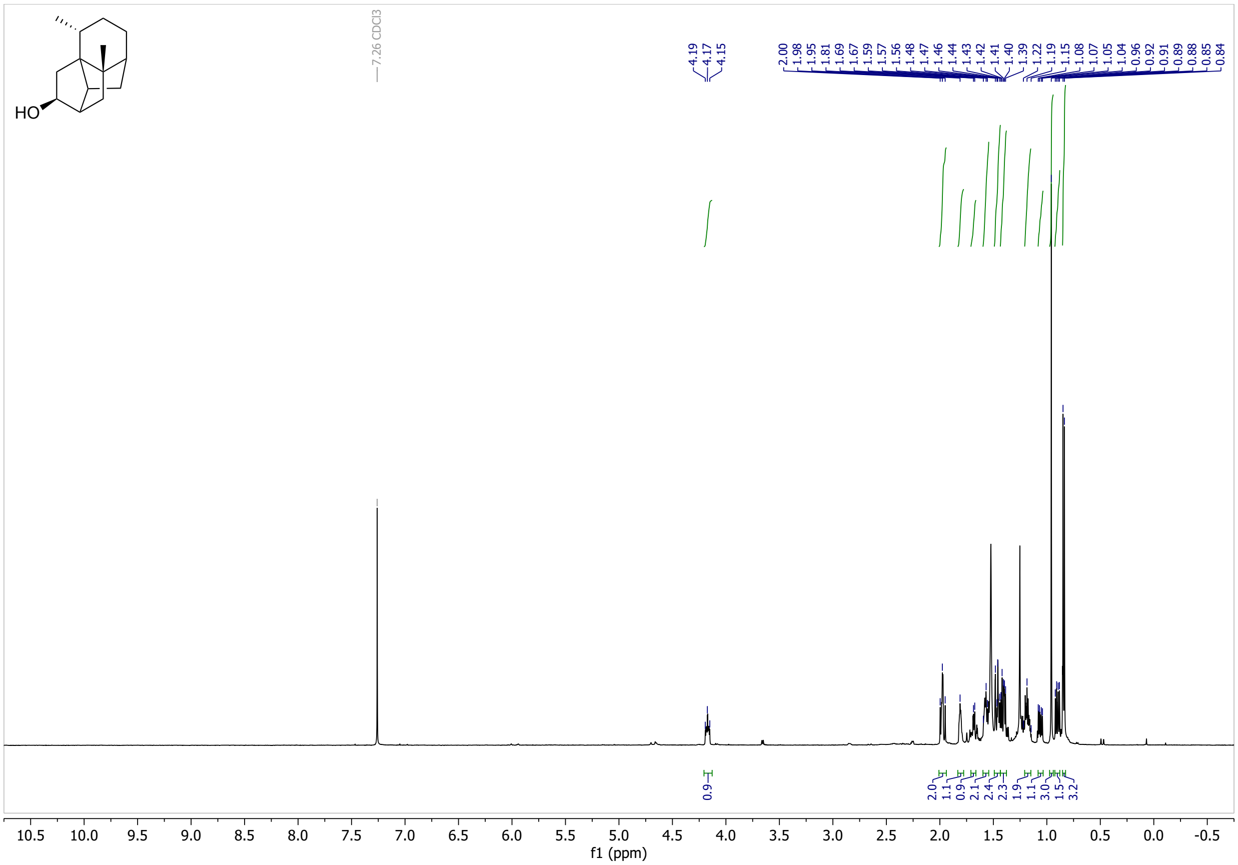

^{13}C NMR (126 MHz, $CDCl_3$) (16)

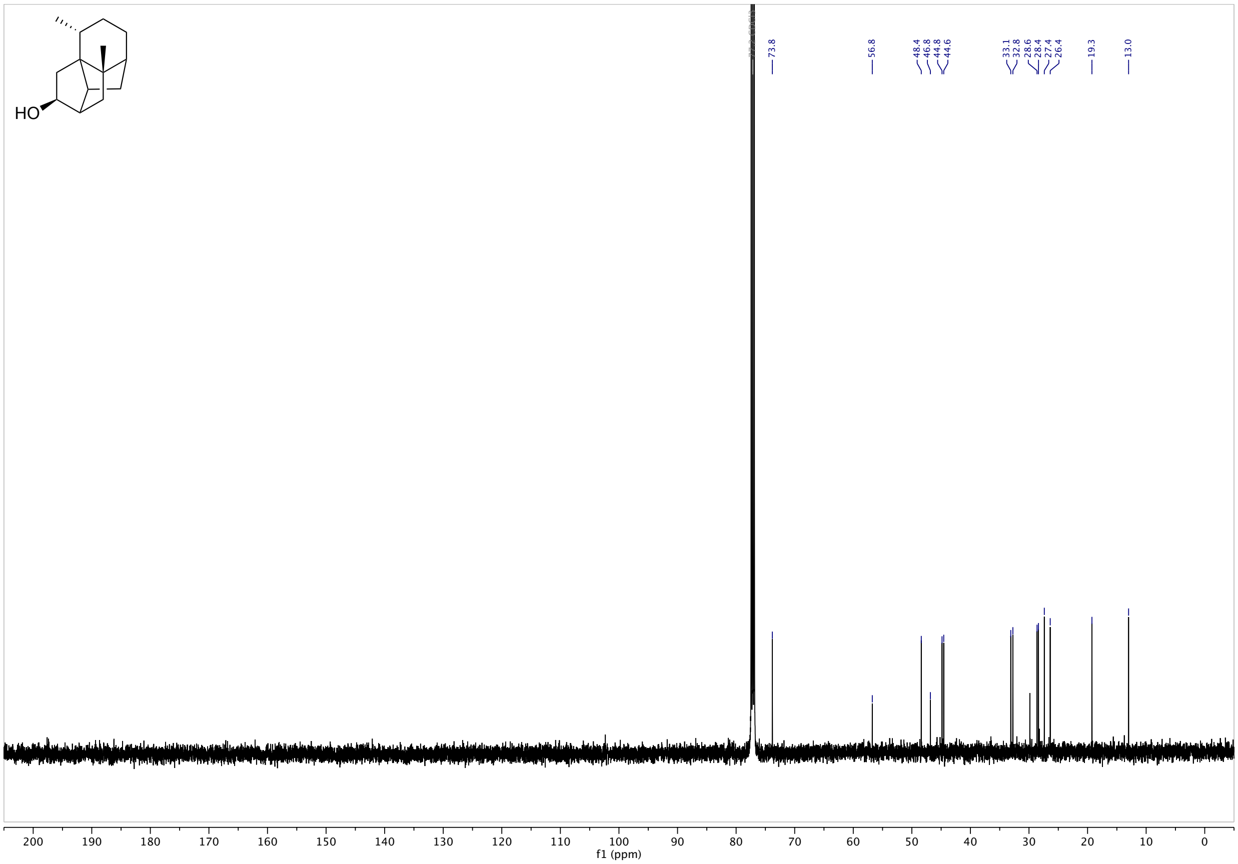

^{1}H NMR (400 MHz, CDCl$_3$) **(18)** – equilibrating isomeric mixture

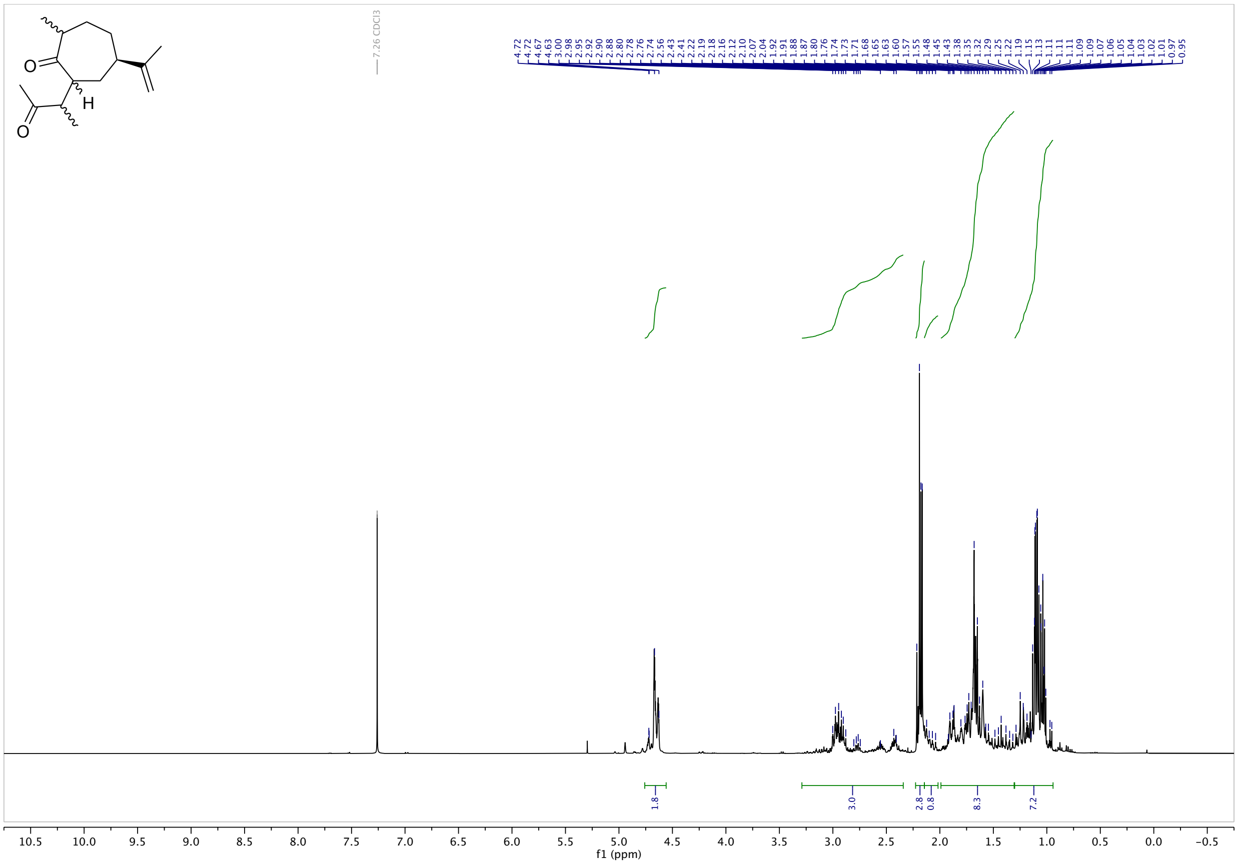

^{1}H NMR (400 MHz, CDCl$_3$) **(19)** – equilibrating isomeric mixture

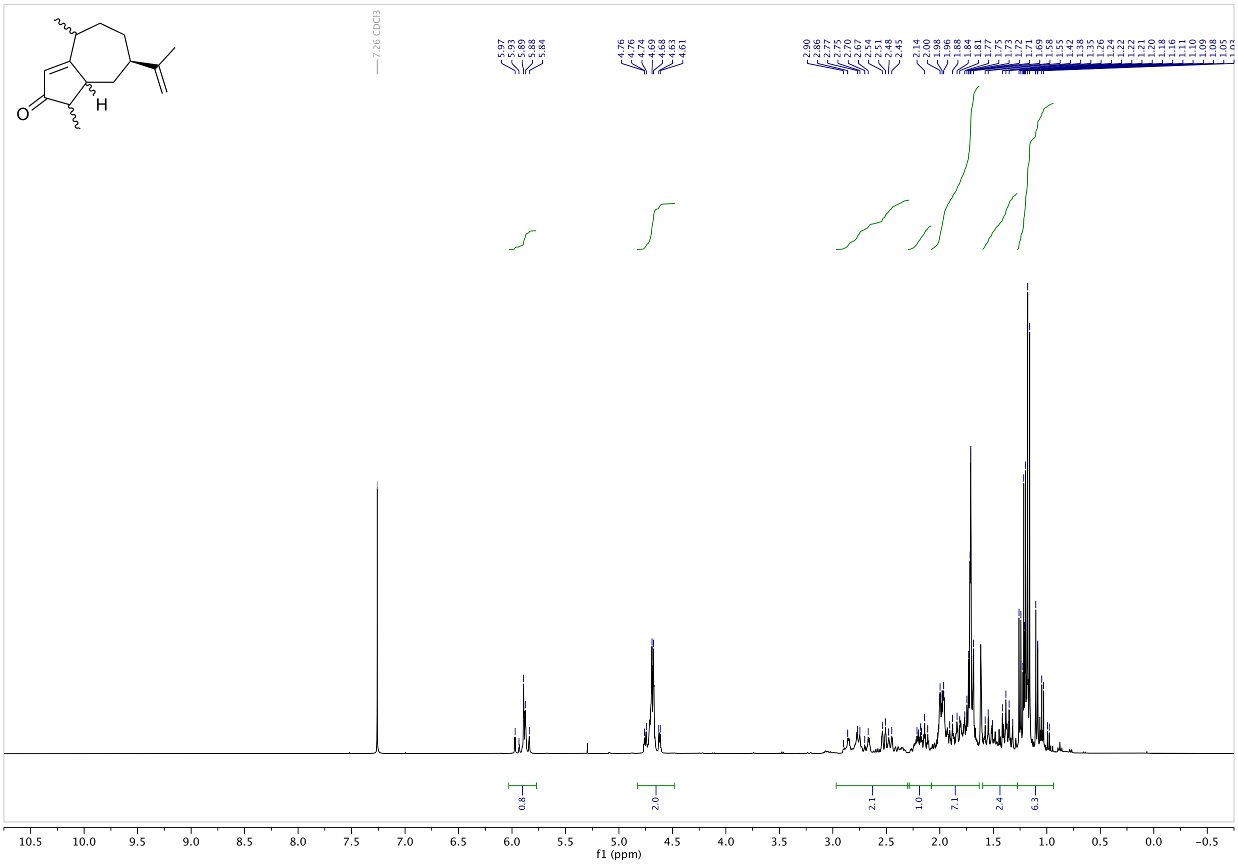

^{1}H NMR (600 MHz, CDCl$_3$) **(20)** (x ether)

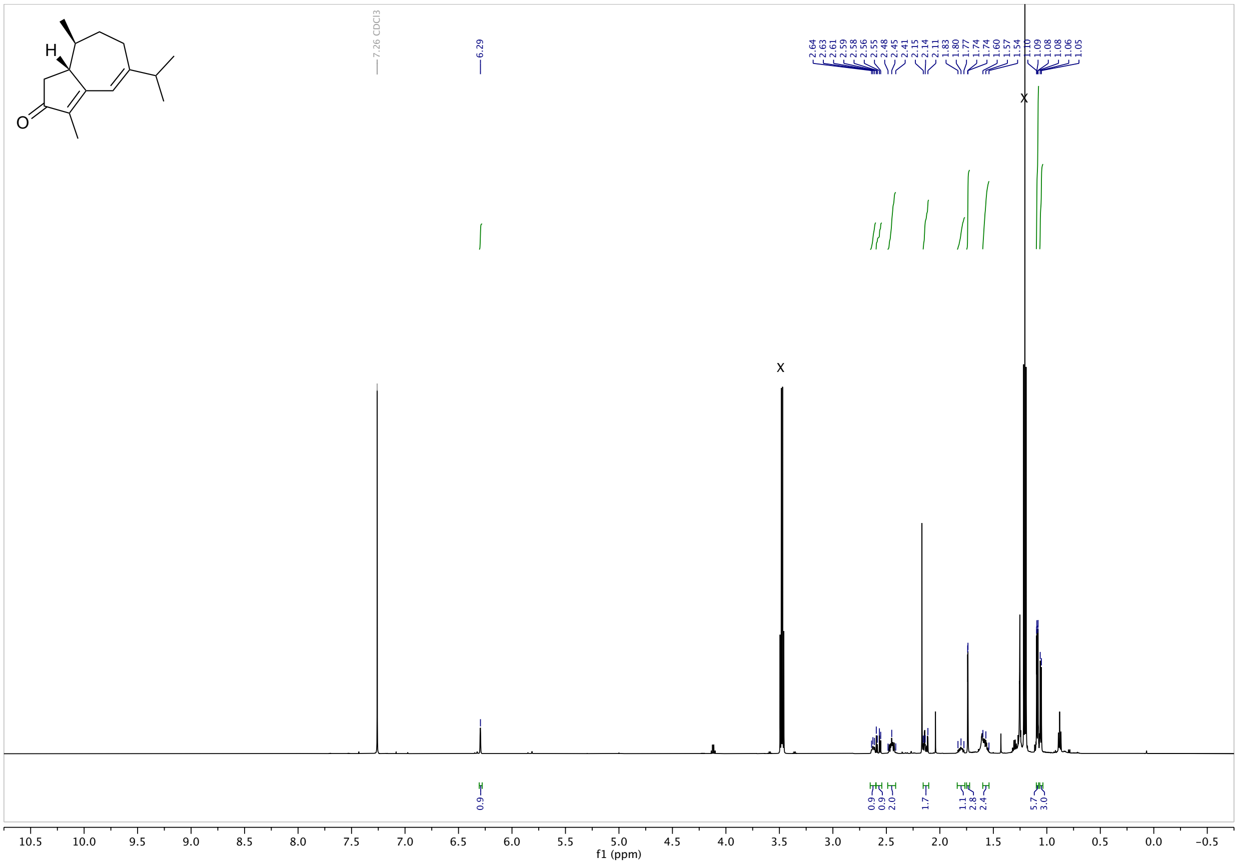

^{13}C NMR (151 MHz, CDCl$_3$) **(20)** (x ether)

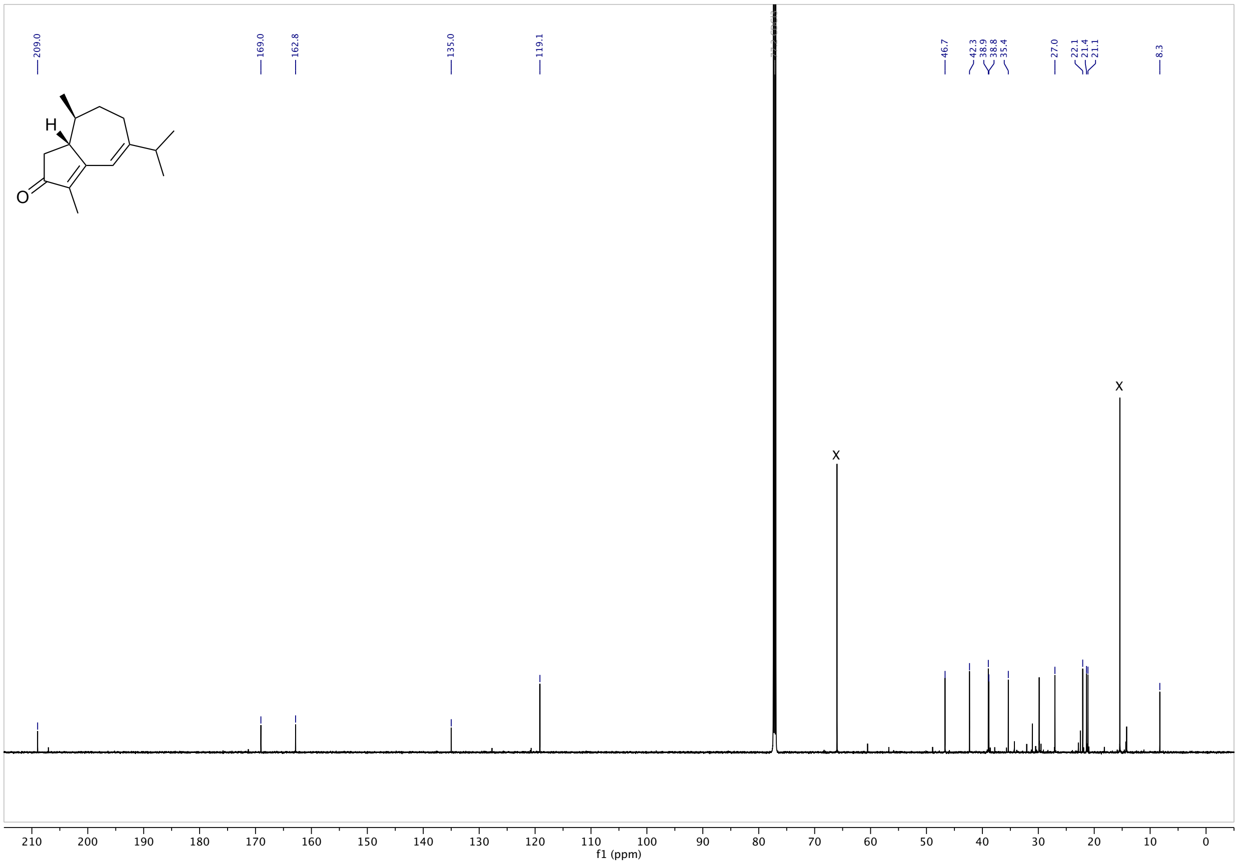

^{1}H NMR (600 MHz, $CDCl_3$) **(21)** (x ether)

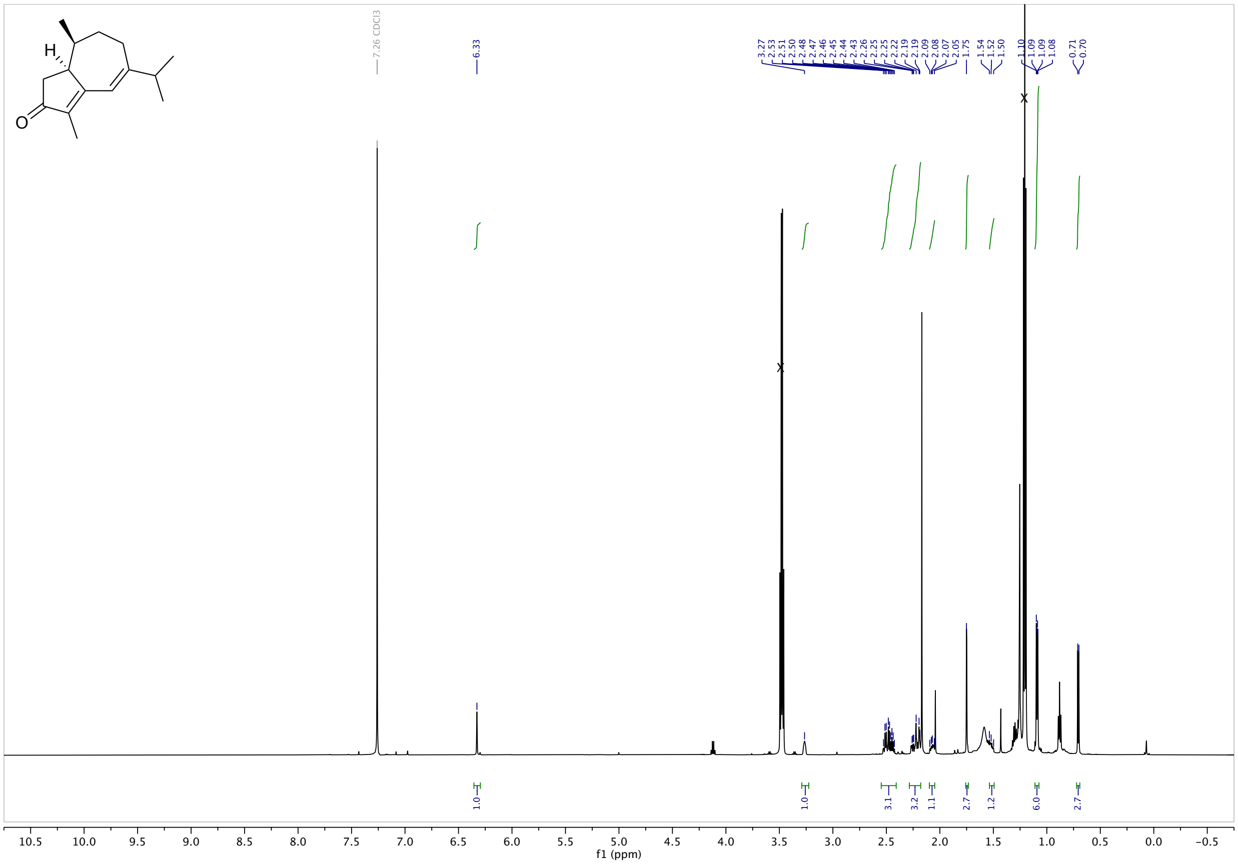

^{13}C NMR (151 MHz, $CDCl_3$) **(21)** (x ether)

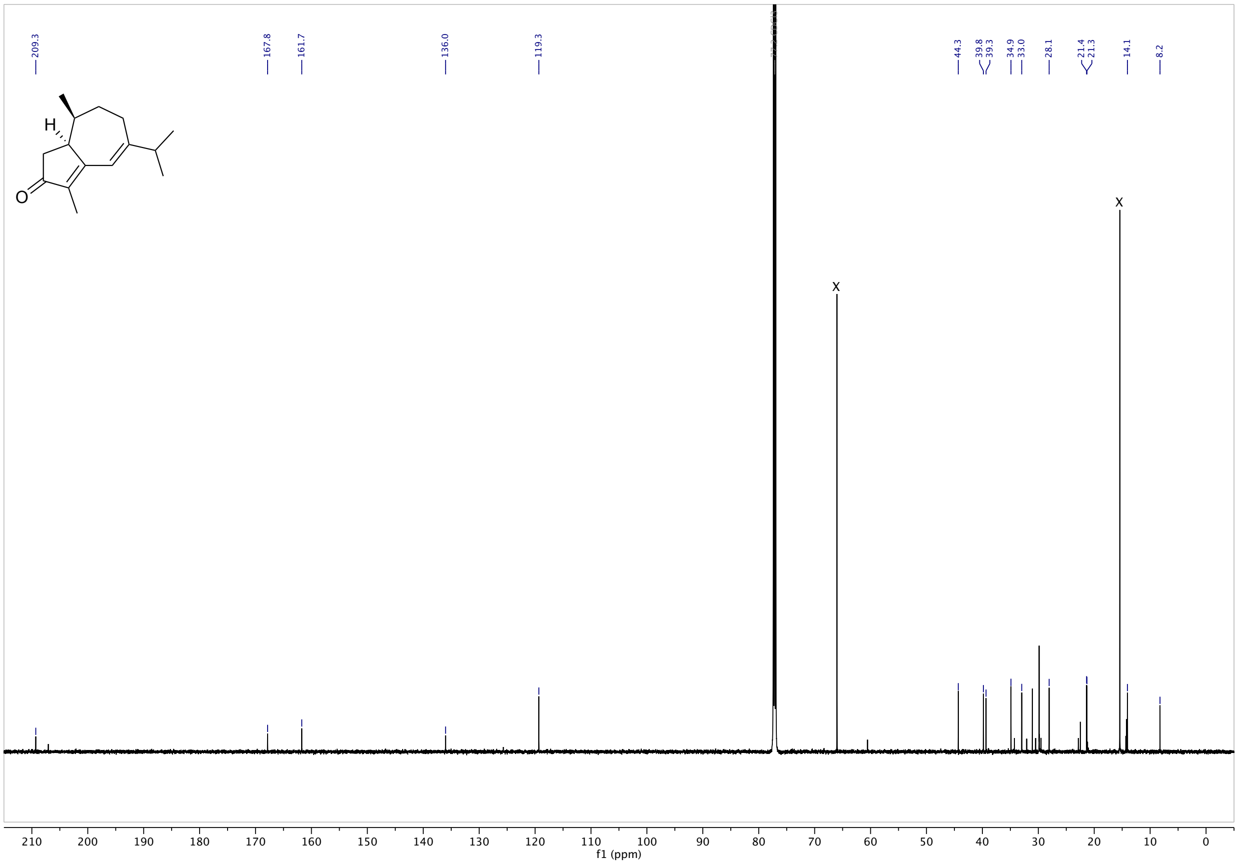

^{1}H NMR (400 MHz, $CDCl_3$) (S2)

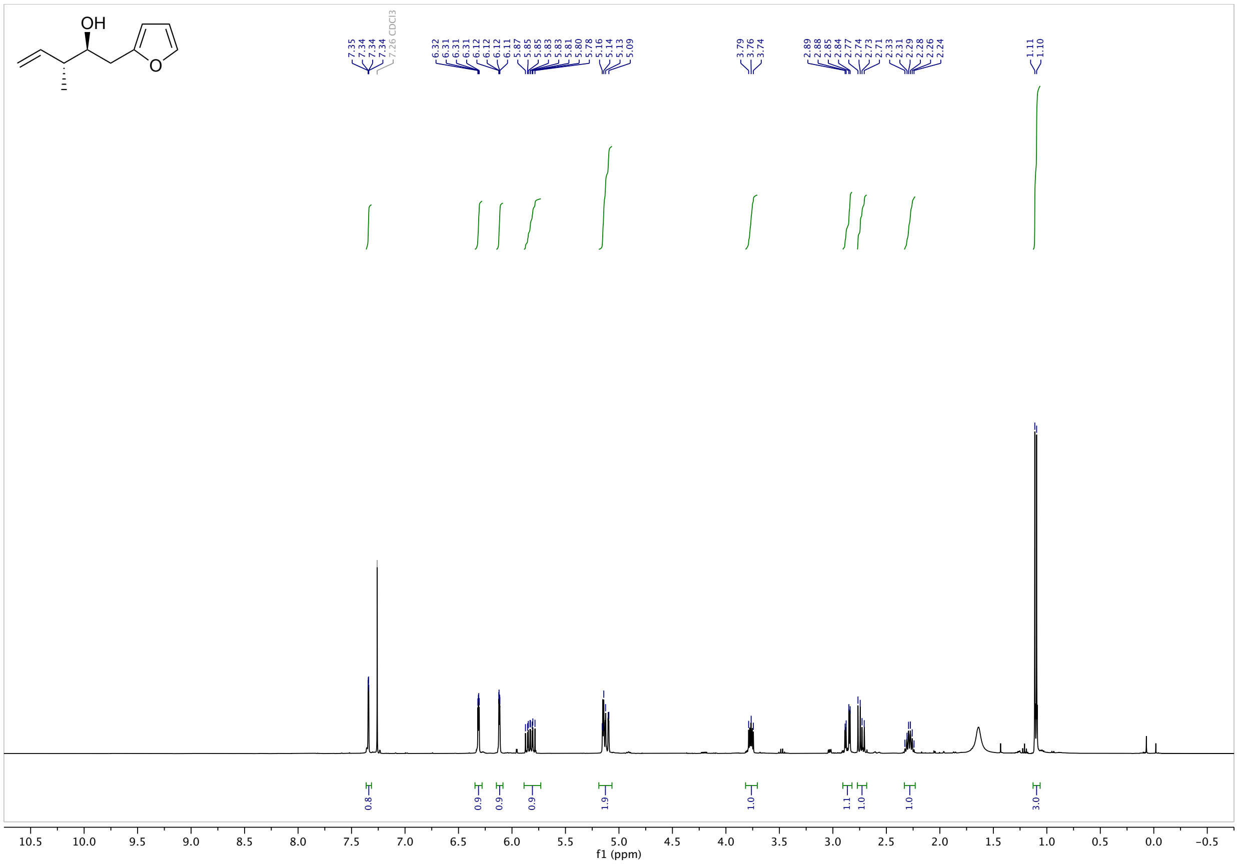

^{13}C NMR (101 MHz, $CDCl_3$) (S2)

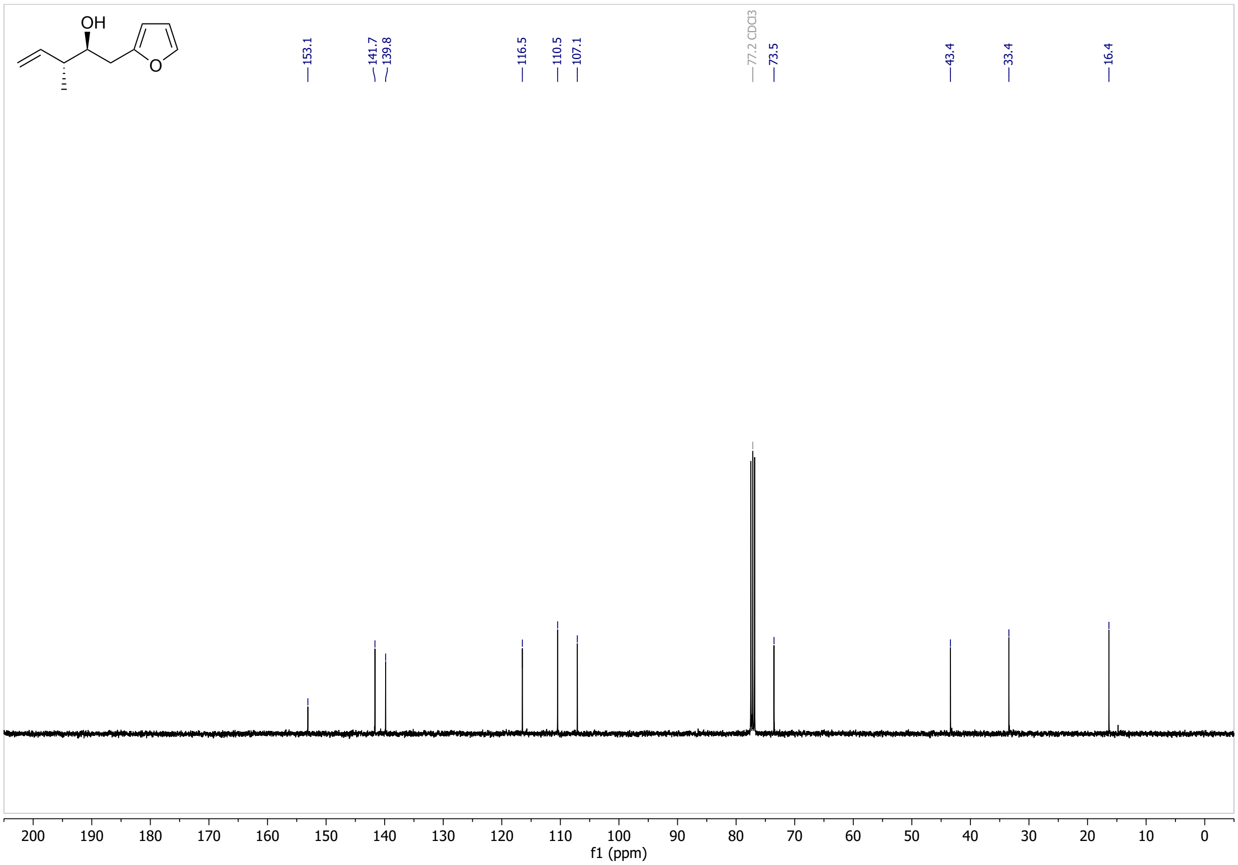

^{1}H NMR (400 MHz, $CDCl_3$) (23)

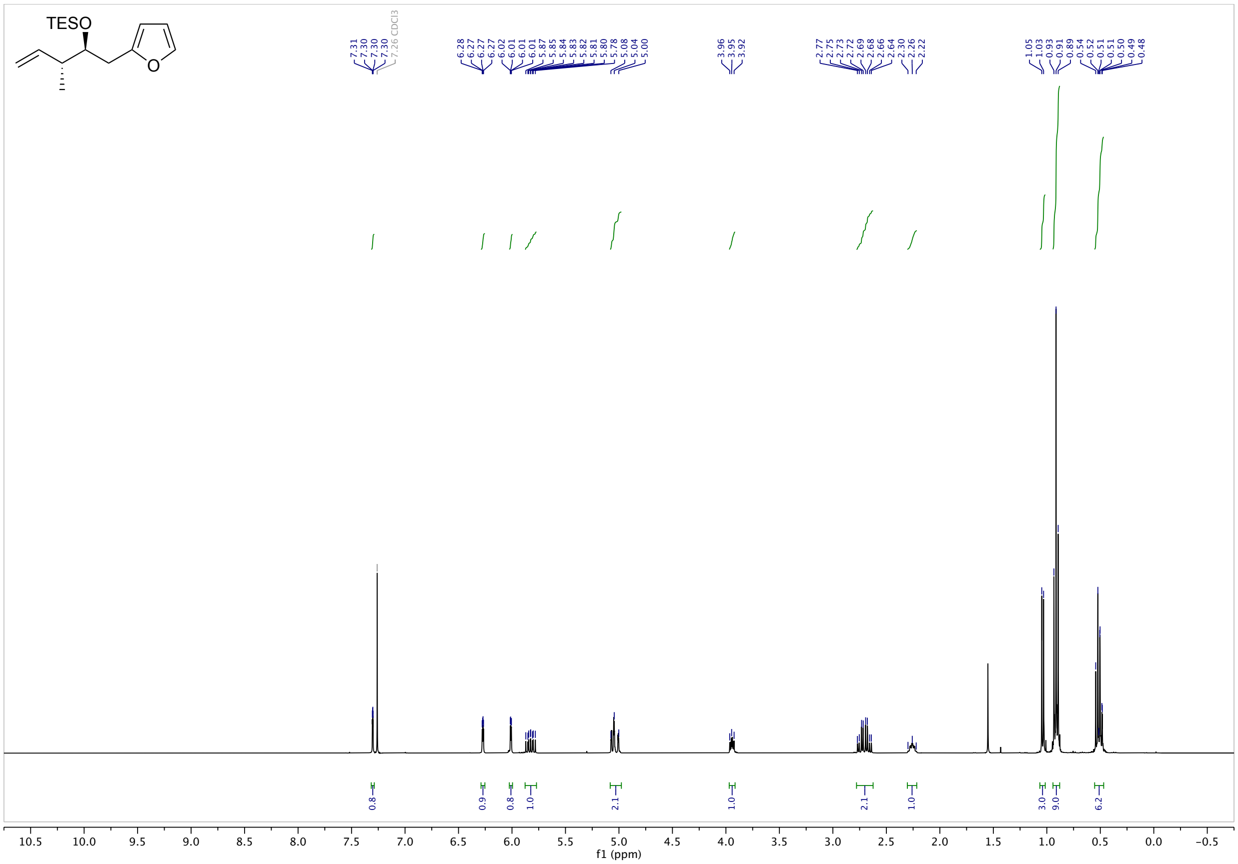

^{13}C NMR (101 MHz, $CDCl_3$) (23)

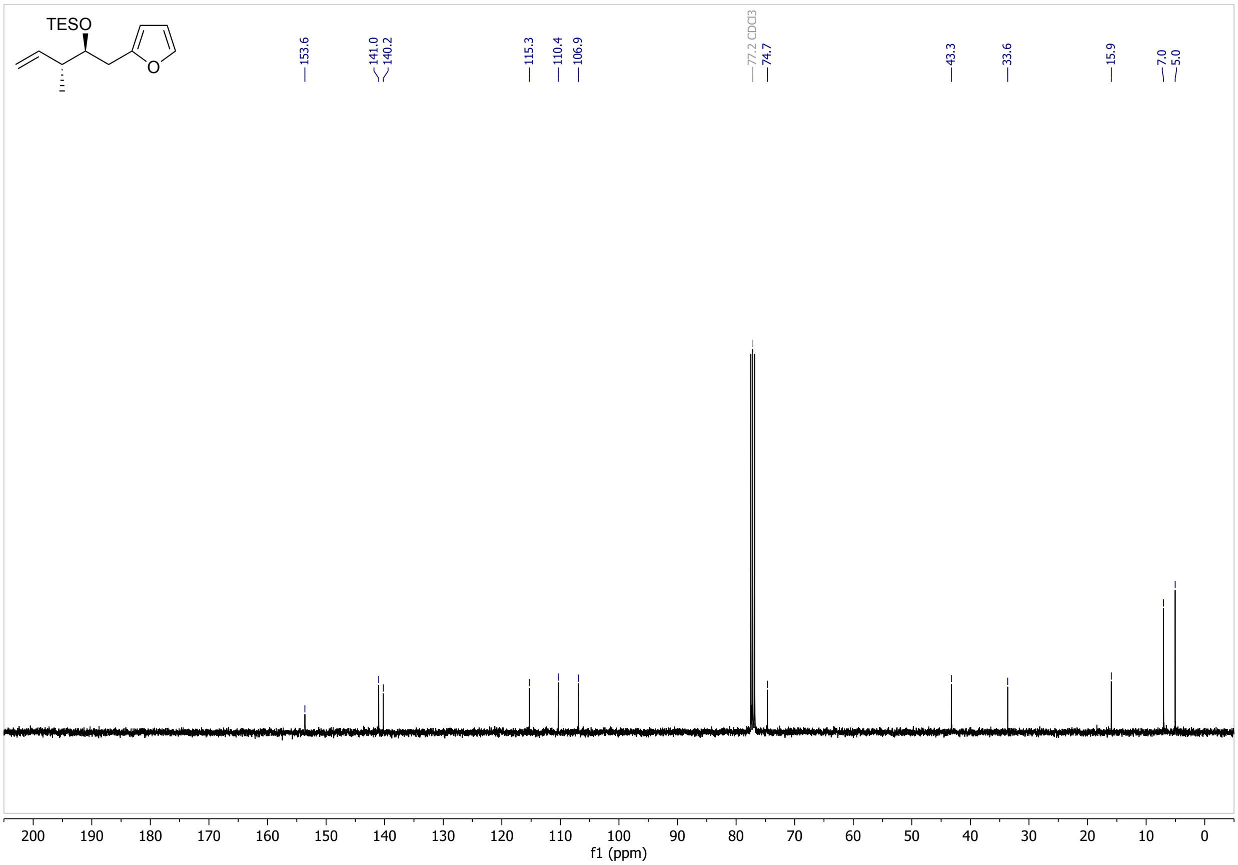

^{1}H NMR (600 MHz, $CDCl_3$) (24)

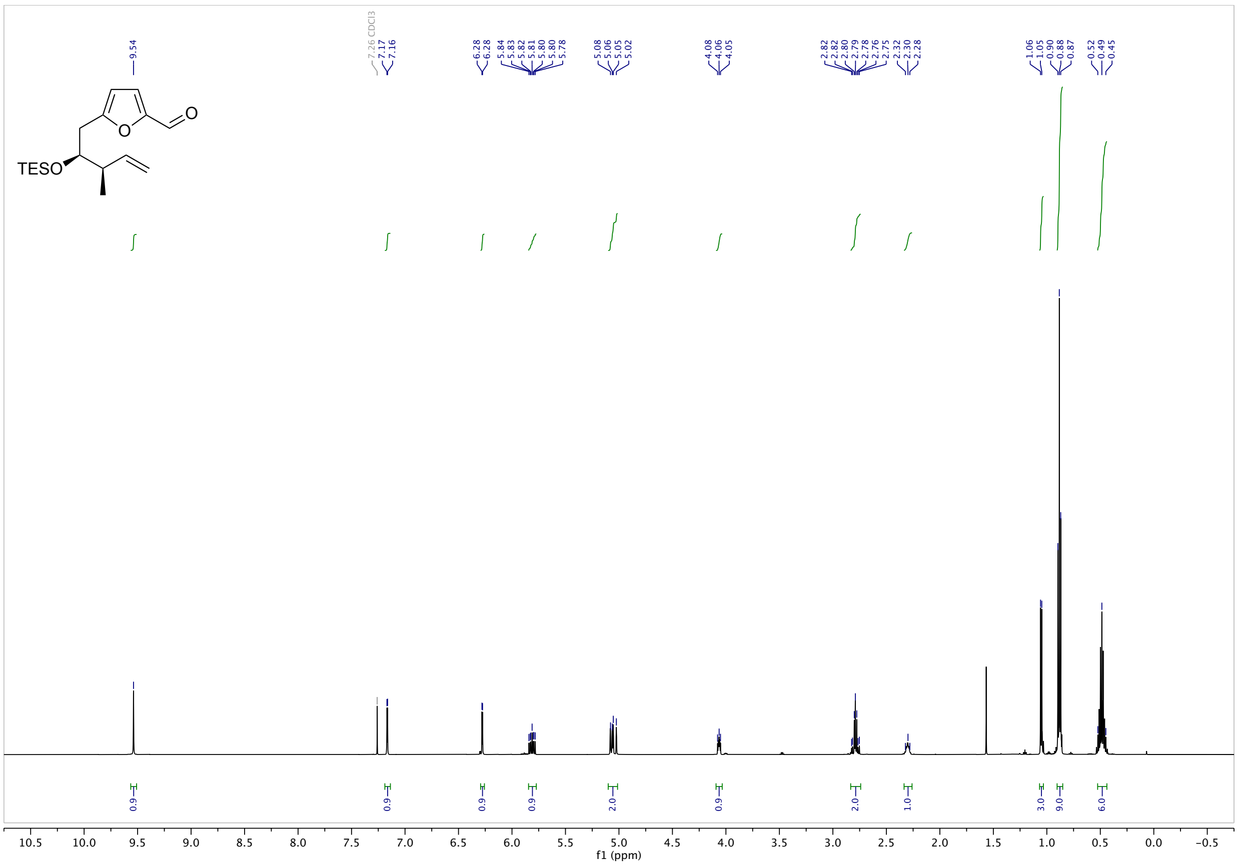

^{13}C NMR (151 MHz, $CDCl_3$) (24)

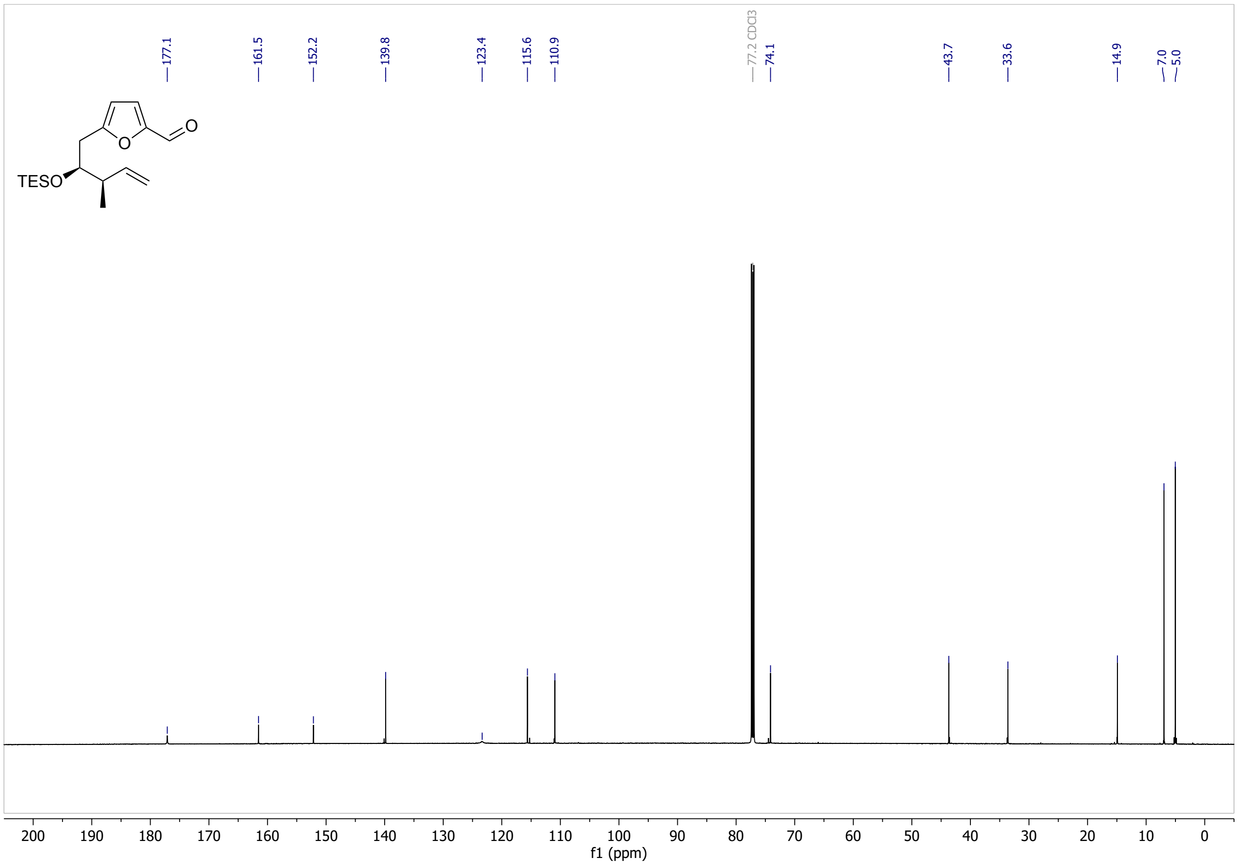

^{1}H NMR (700 MHz, $CDCl_3$) (25)

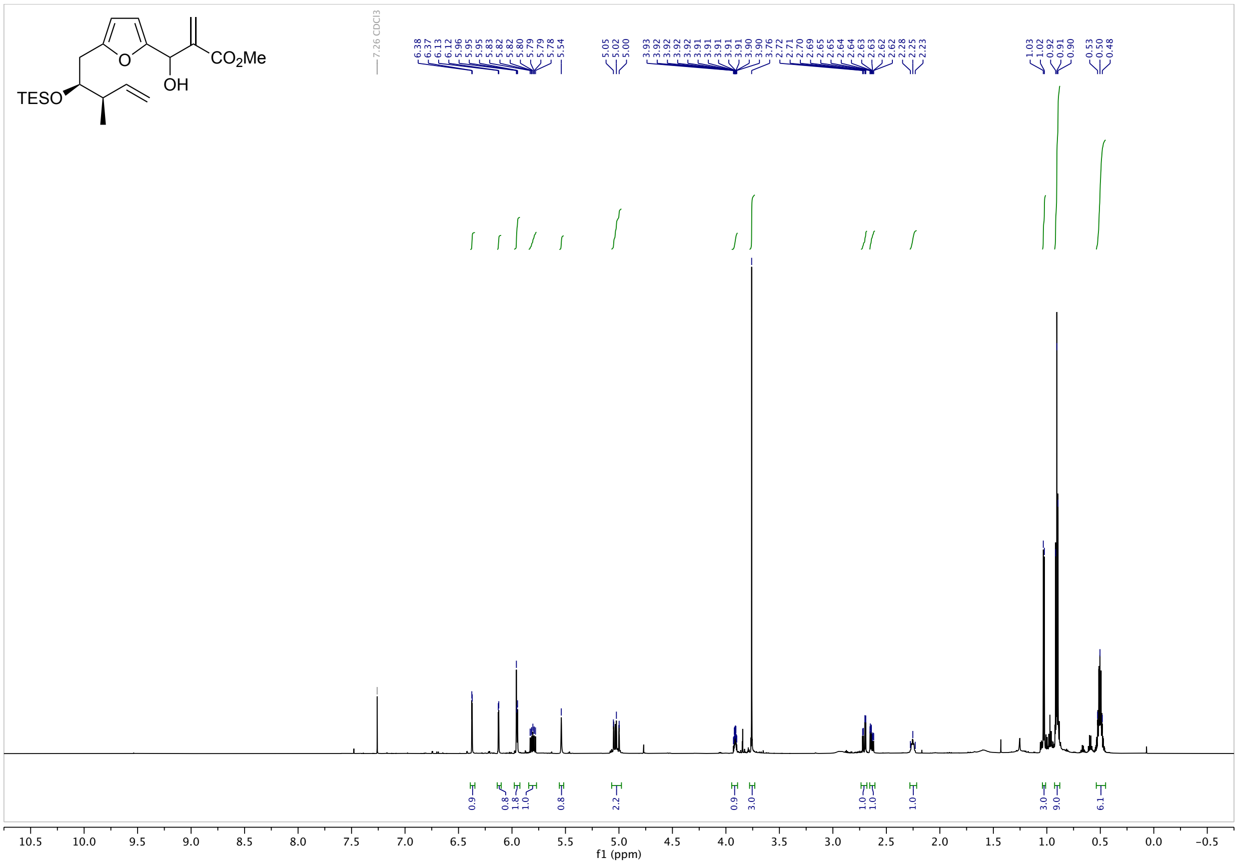

^{13}C NMR (176 MHz, $CDCl_3$) (25)

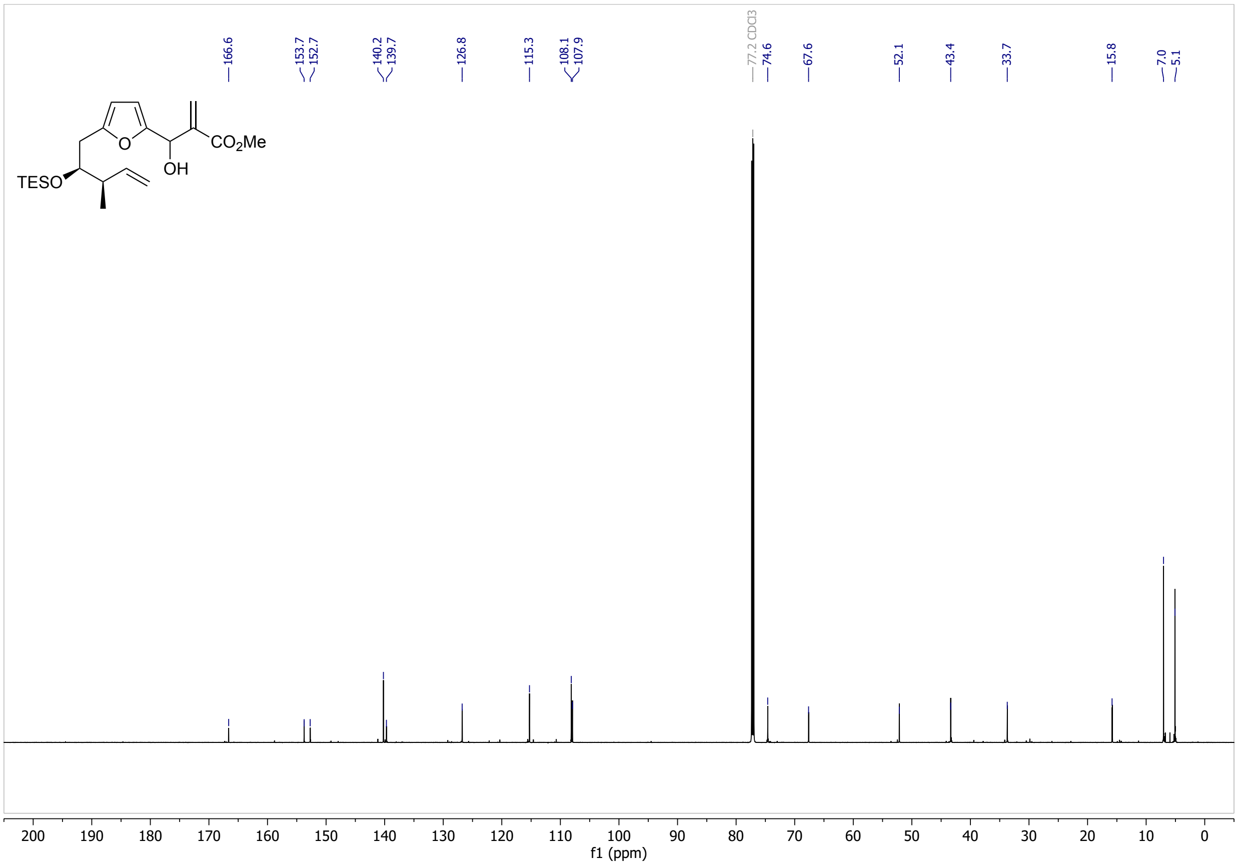

^{1}H NMR (400 MHz, CDCl$_3$) **(26)** – first diastereomer

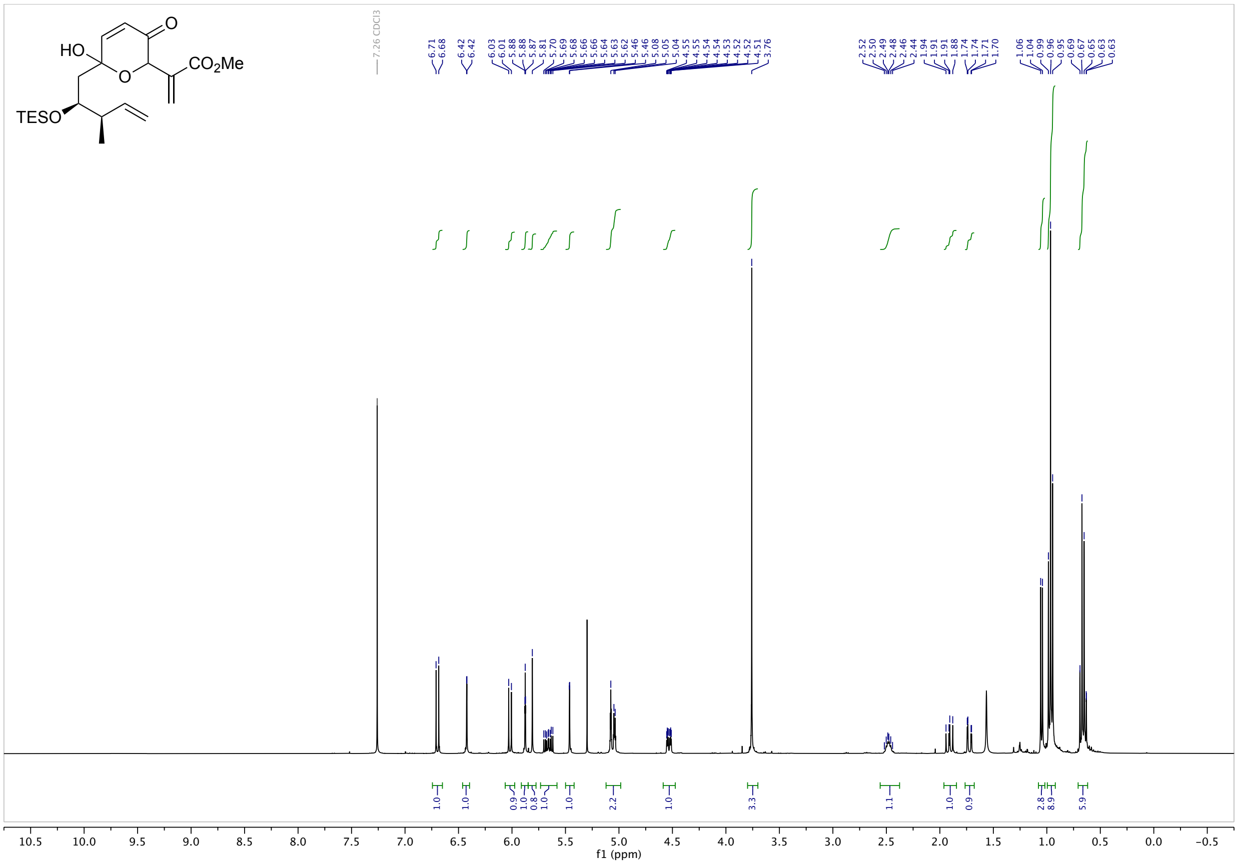

^{13}C NMR (101 MHz, CDCl$_3$) **(26)** – first diastereomer

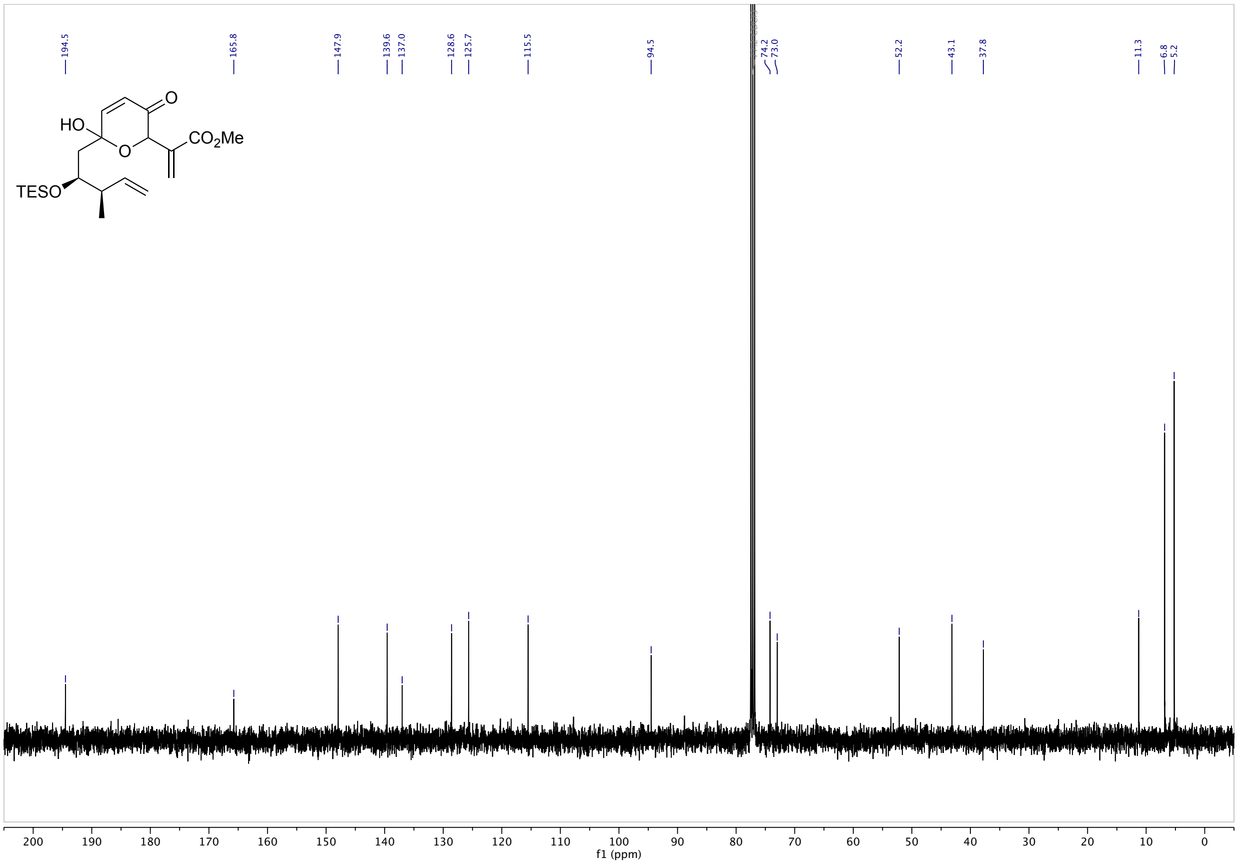

^{1}H NMR (400 MHz, $CDCl_3$) **(26)** – second (+ minor third) diastereomer

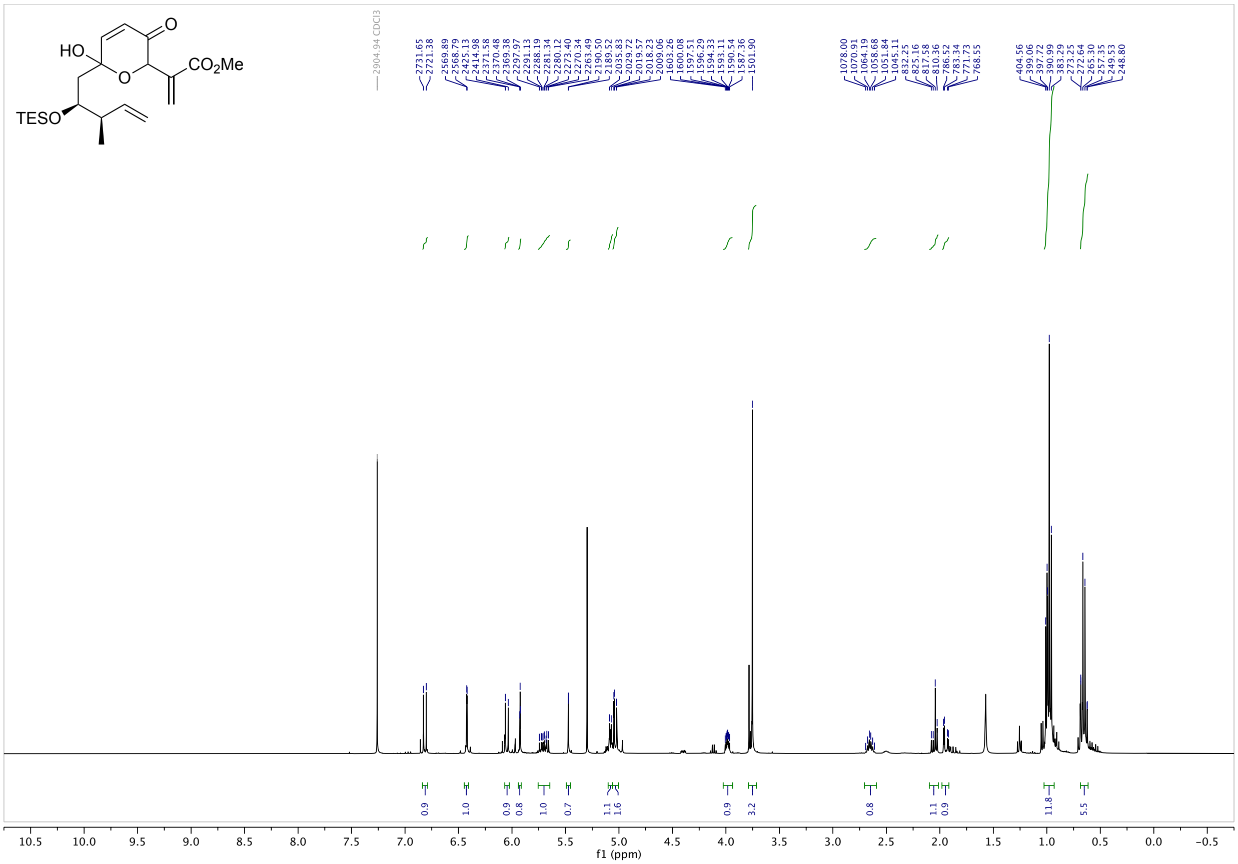

^{13}C NMR (101 MHz, $CDCl_3$) **(26)** – second (+ minor third) diastereomer

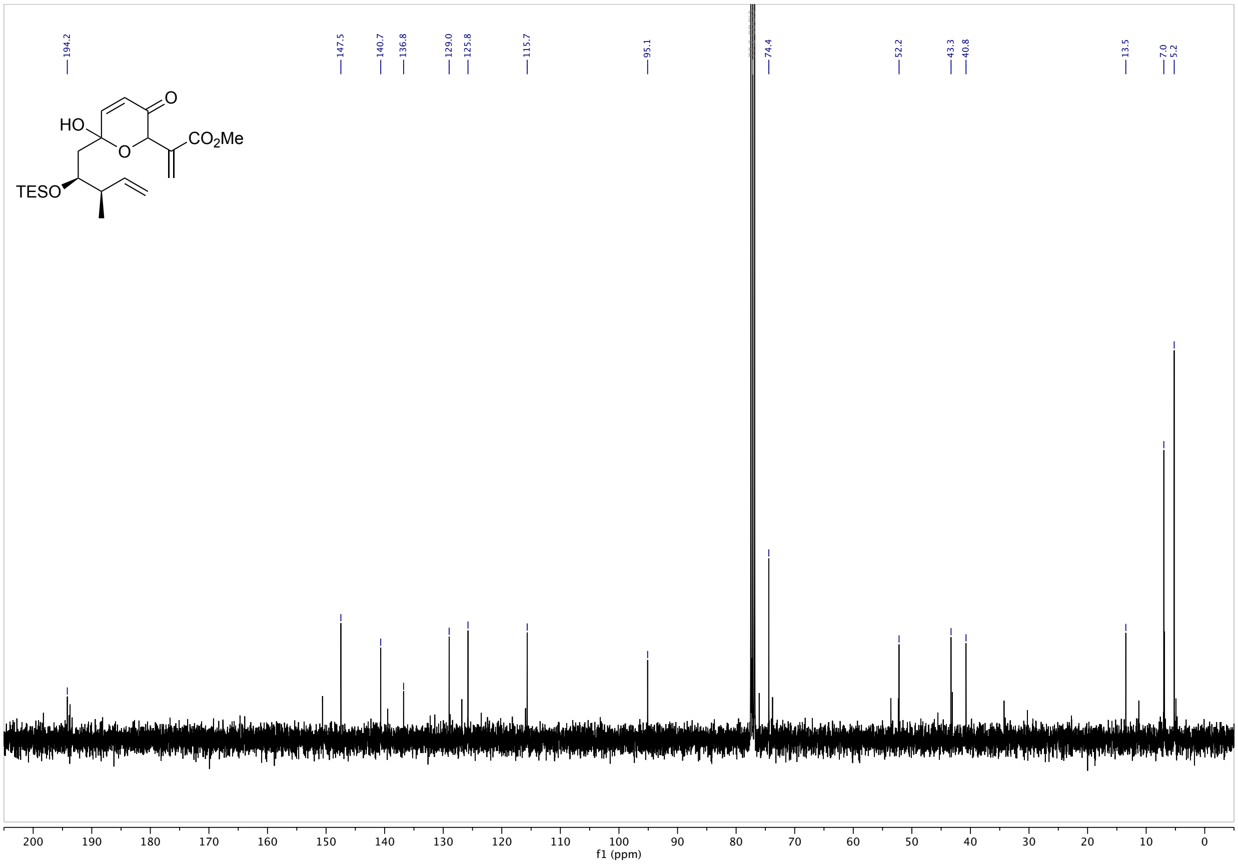

^{1}H NMR (400 MHz, $CDCl_3$) (27)

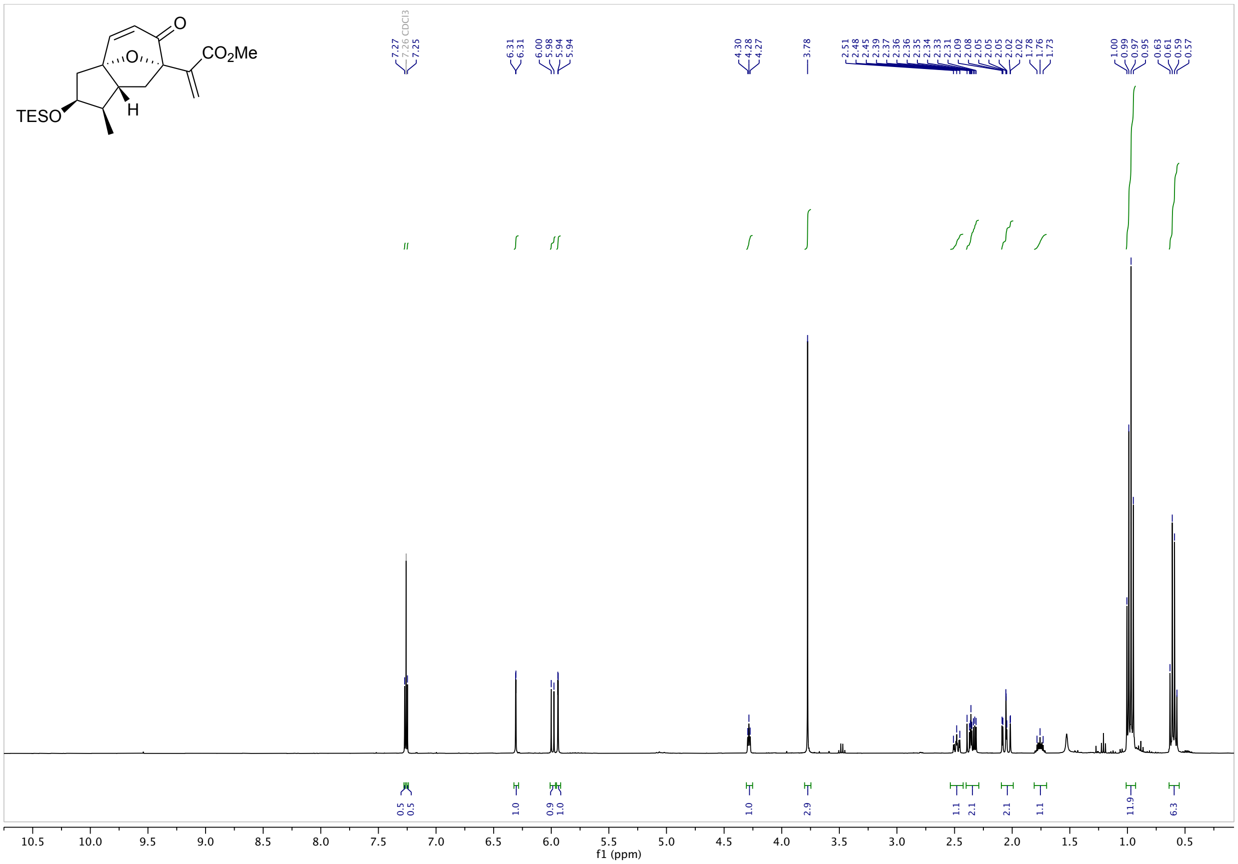

^{13}C NMR (101 MHz, $CDCl_3$) (27)

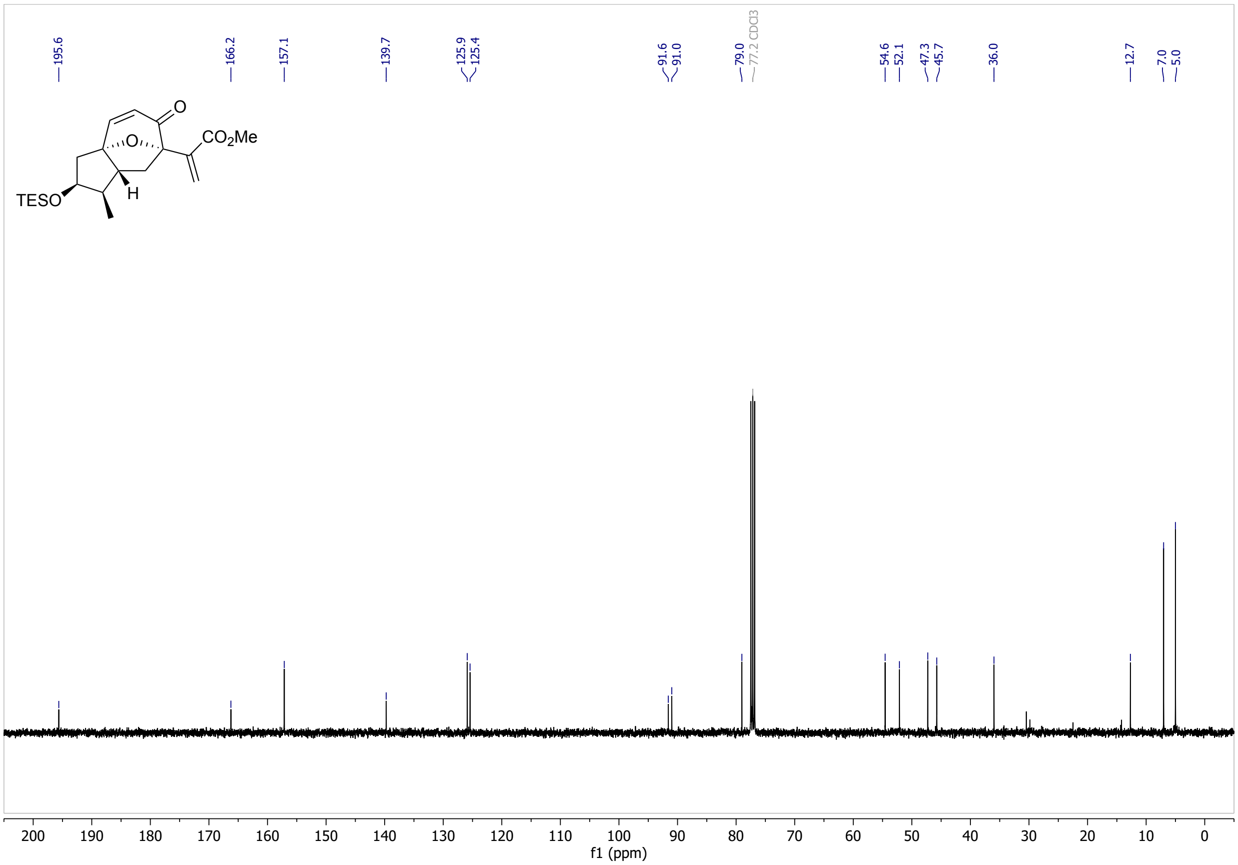

^{1}H NMR (400 MHz, $CDCl_3$) (28)

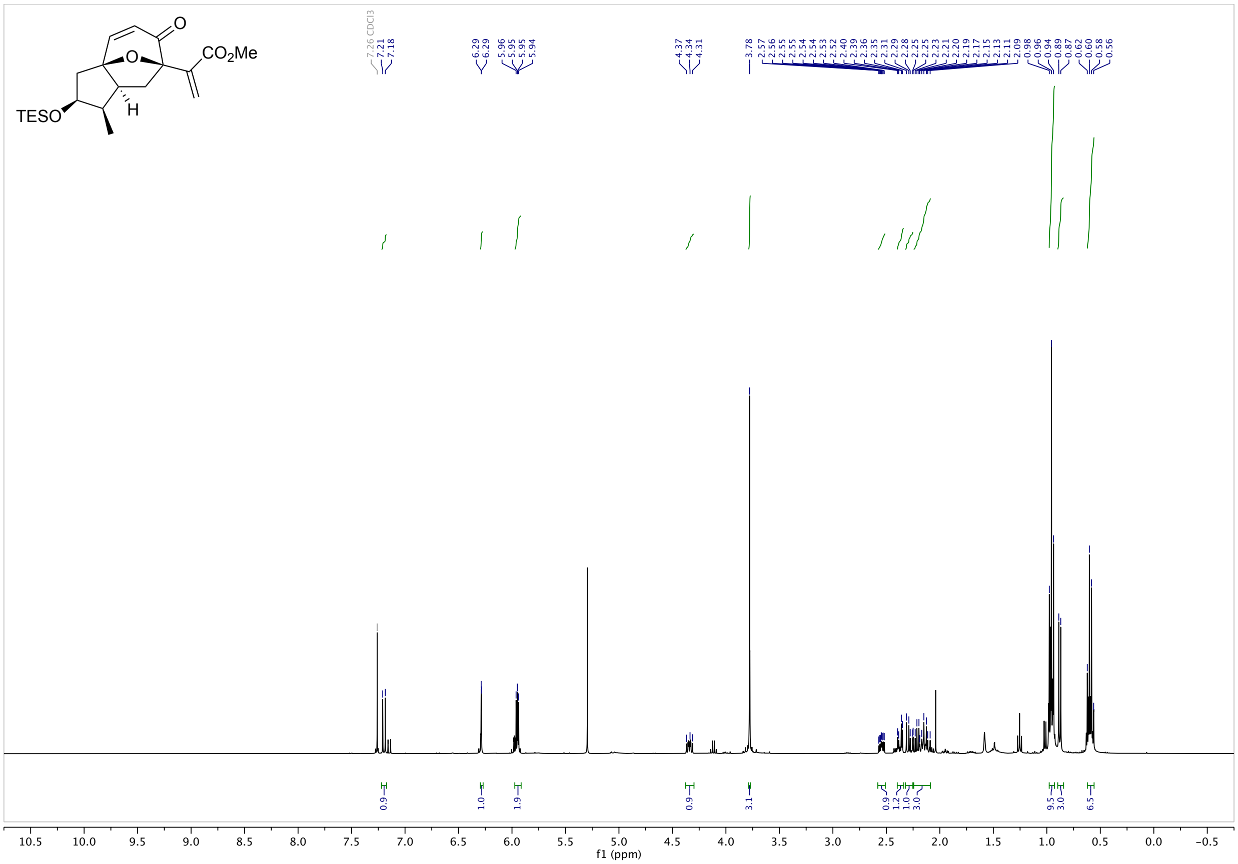

^{13}C NMR (101 MHz, $CDCl_3$) (28)

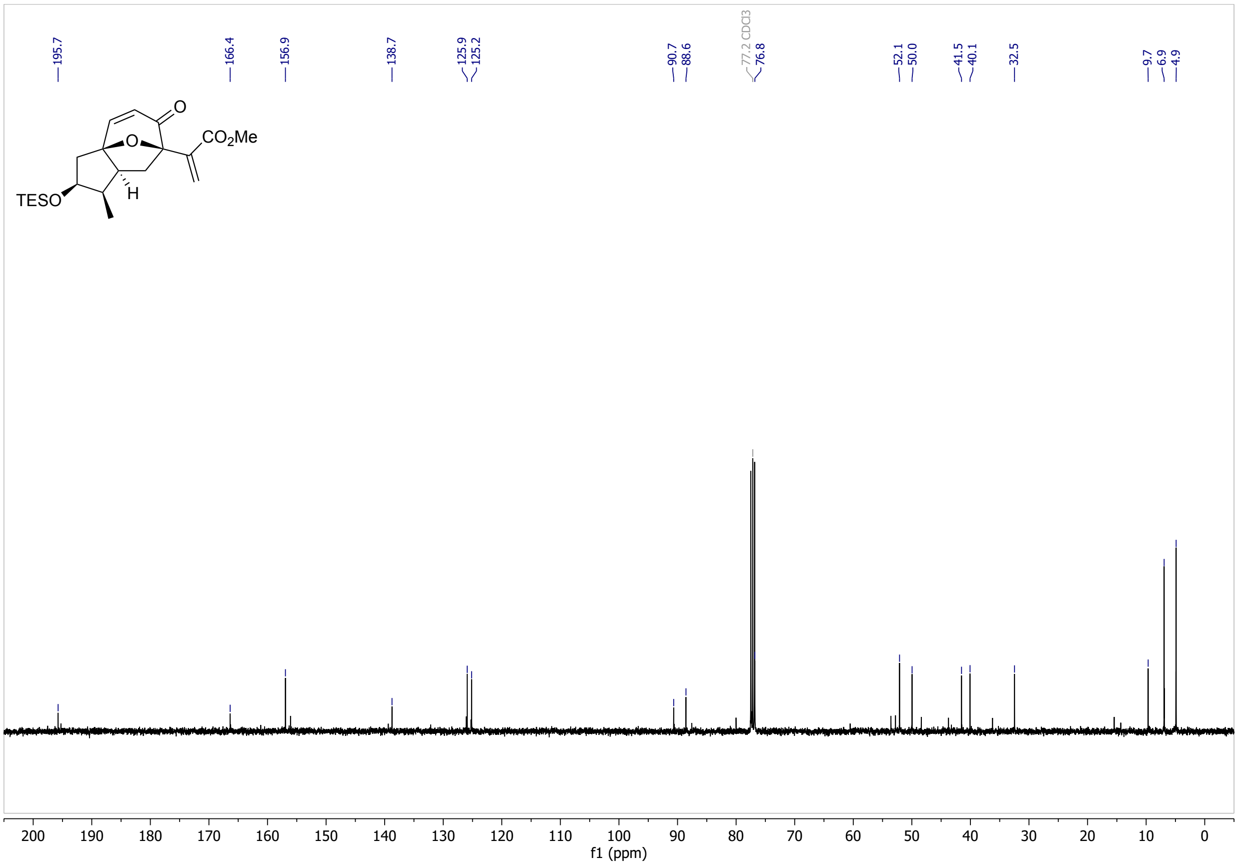

^{1}H NMR (400 MHz, $CDCl_3$) (29)

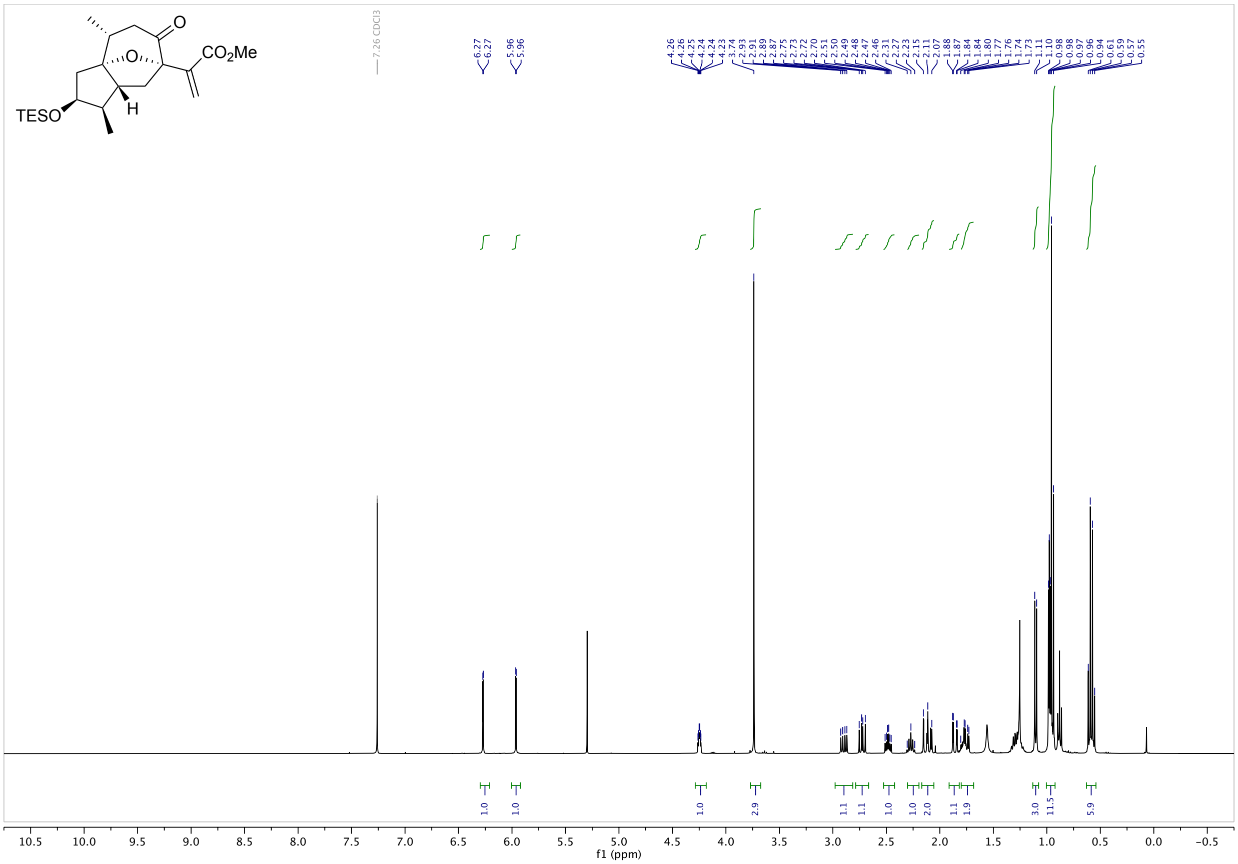

^{13}C NMR (101 MHz, $CDCl_3$) (29)

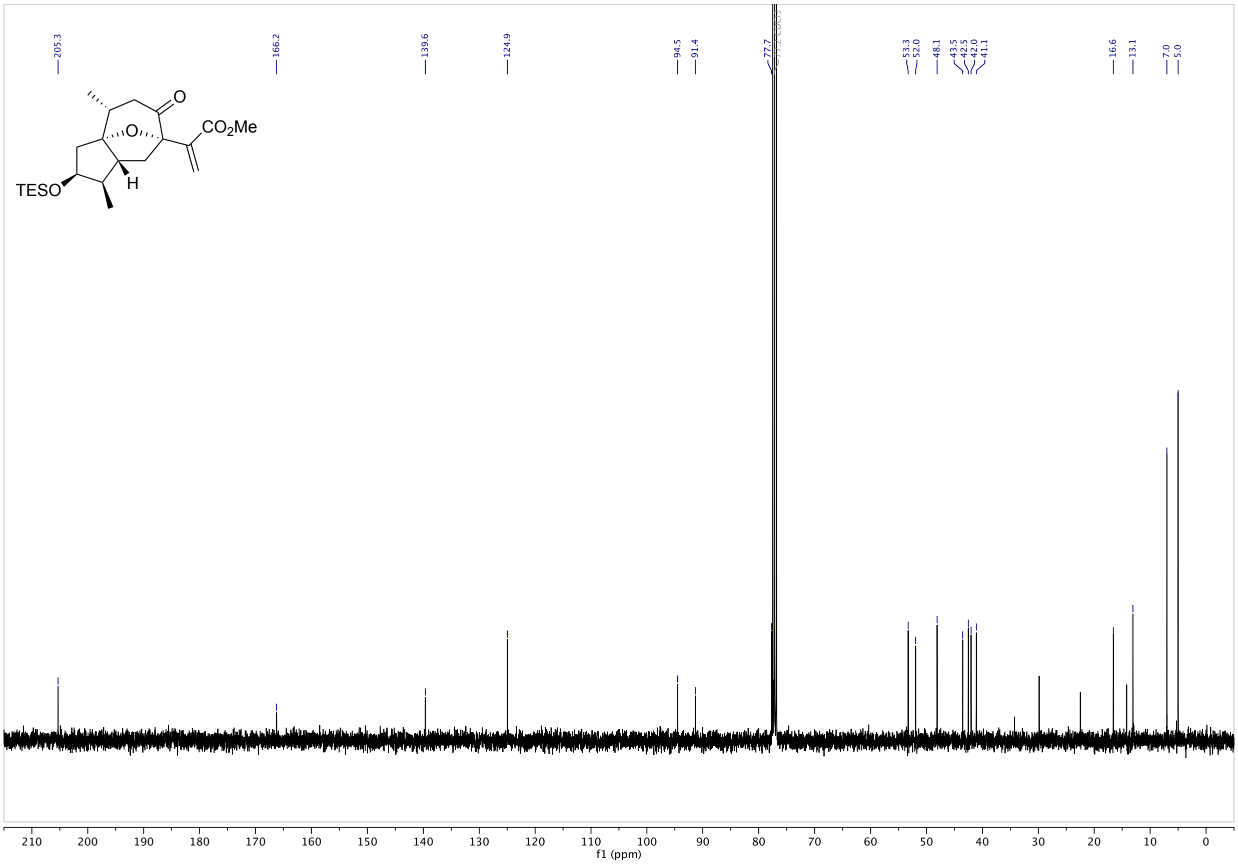

^{1}H NMR (600 MHz, $CDCl_3$) (S3)

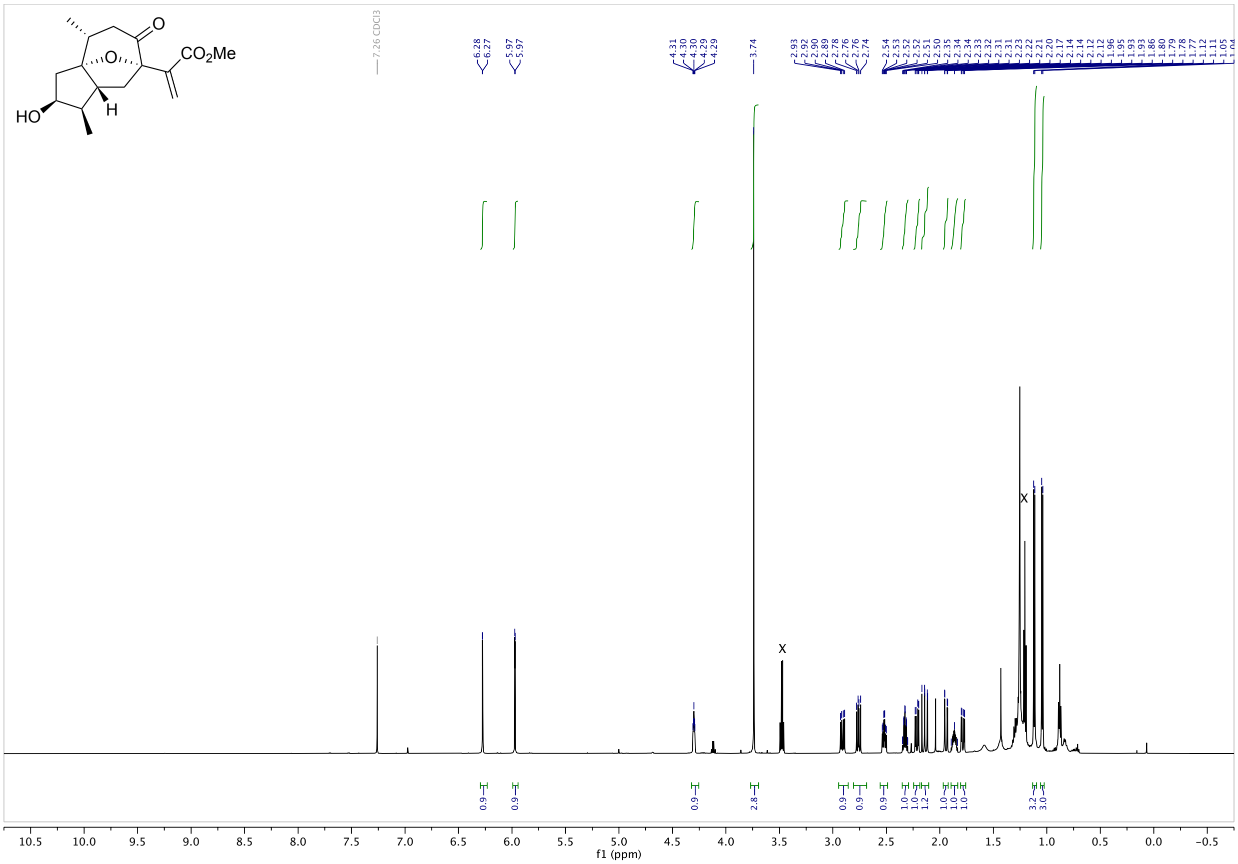

^{13}C NMR (151 MHz, $CDCl_3$) (S3)

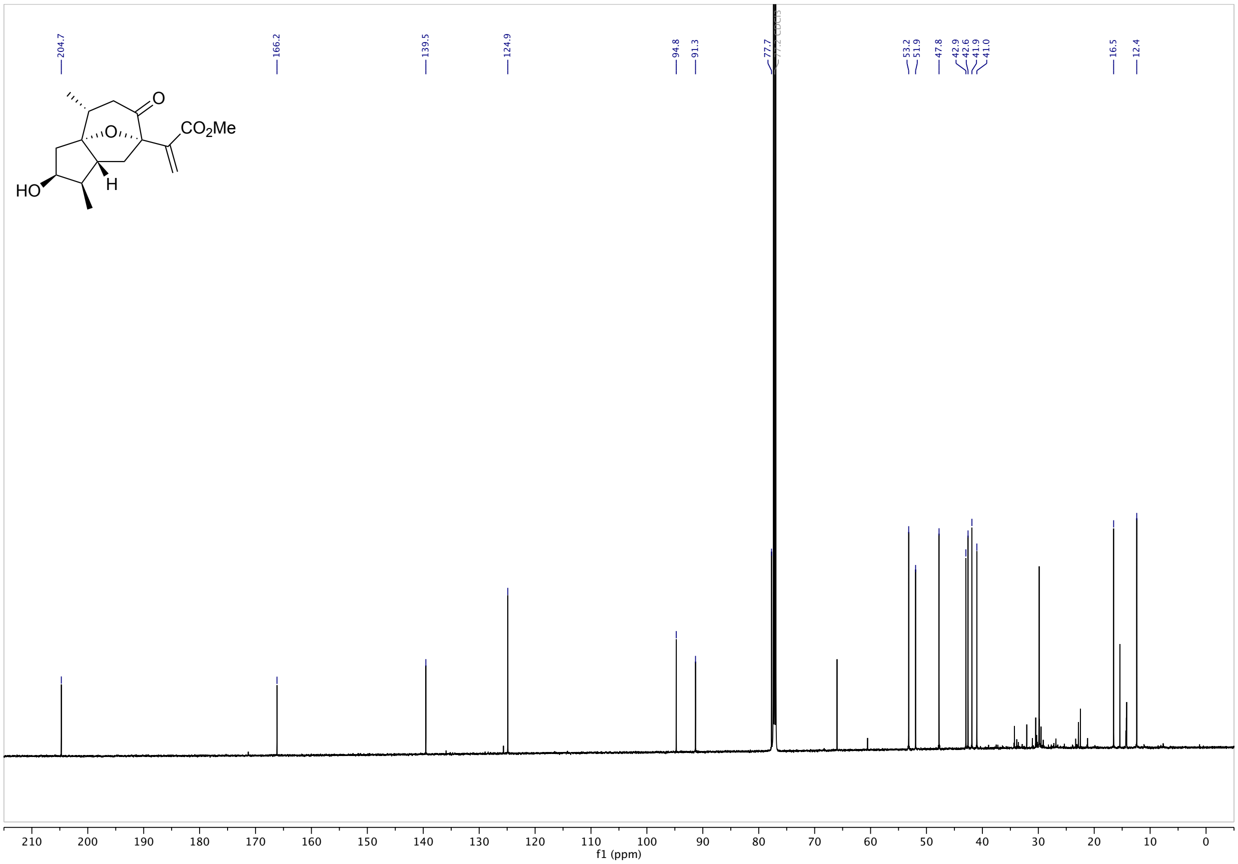

^{1}H NMR (700 MHz, $CDCl_3$) (30)

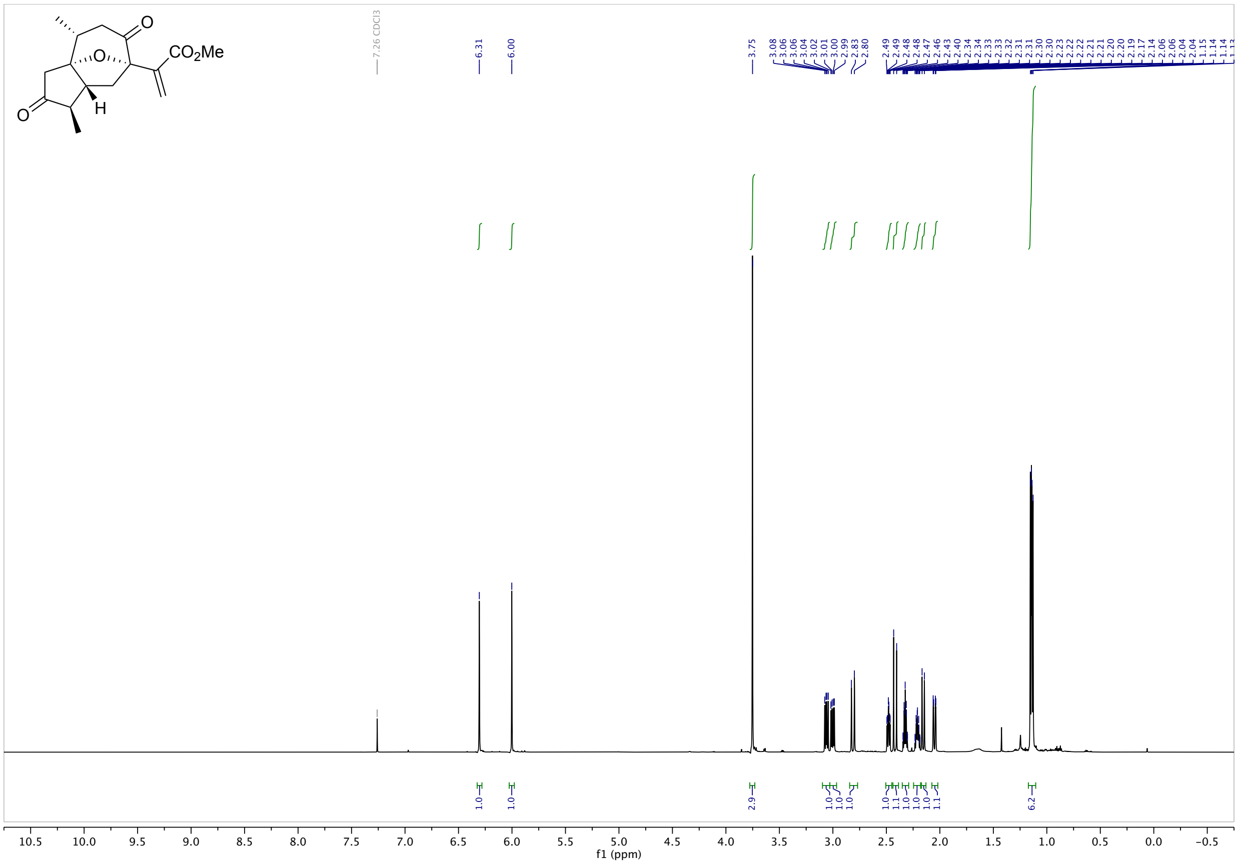

^{13}C NMR (176 MHz, $CDCl_3$) (30)

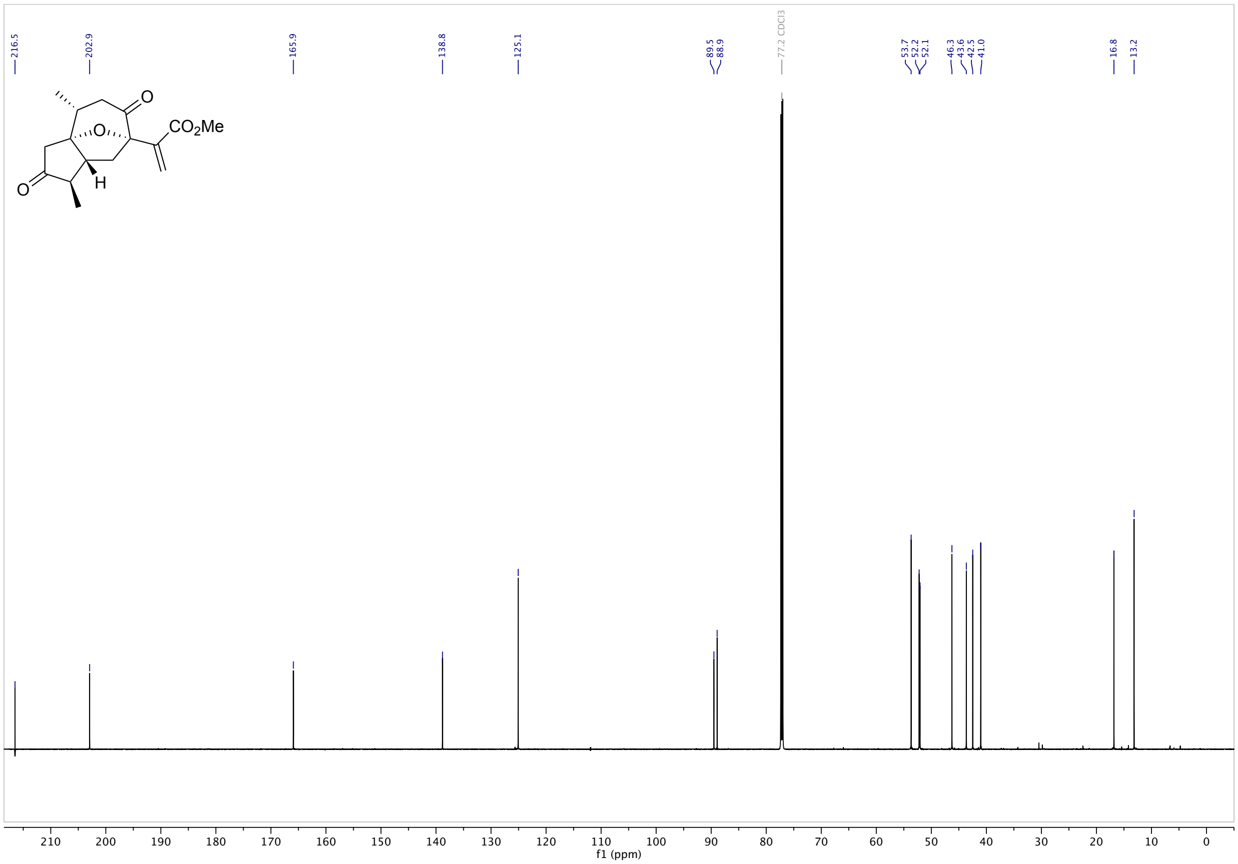

^{1}H NMR (600 MHz, $CDCl_3$) (S4)

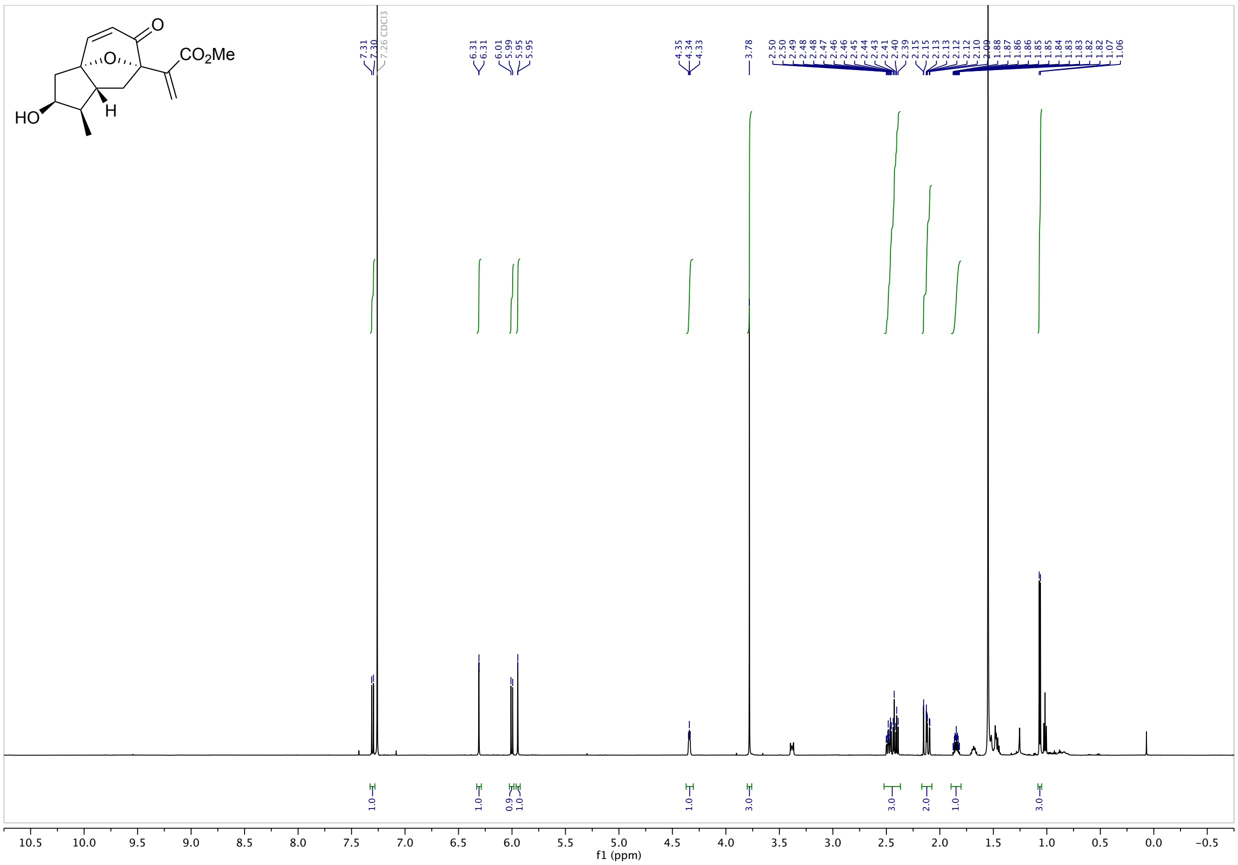

^{13}C NMR (151 MHz, $CDCl_3$) (S4)

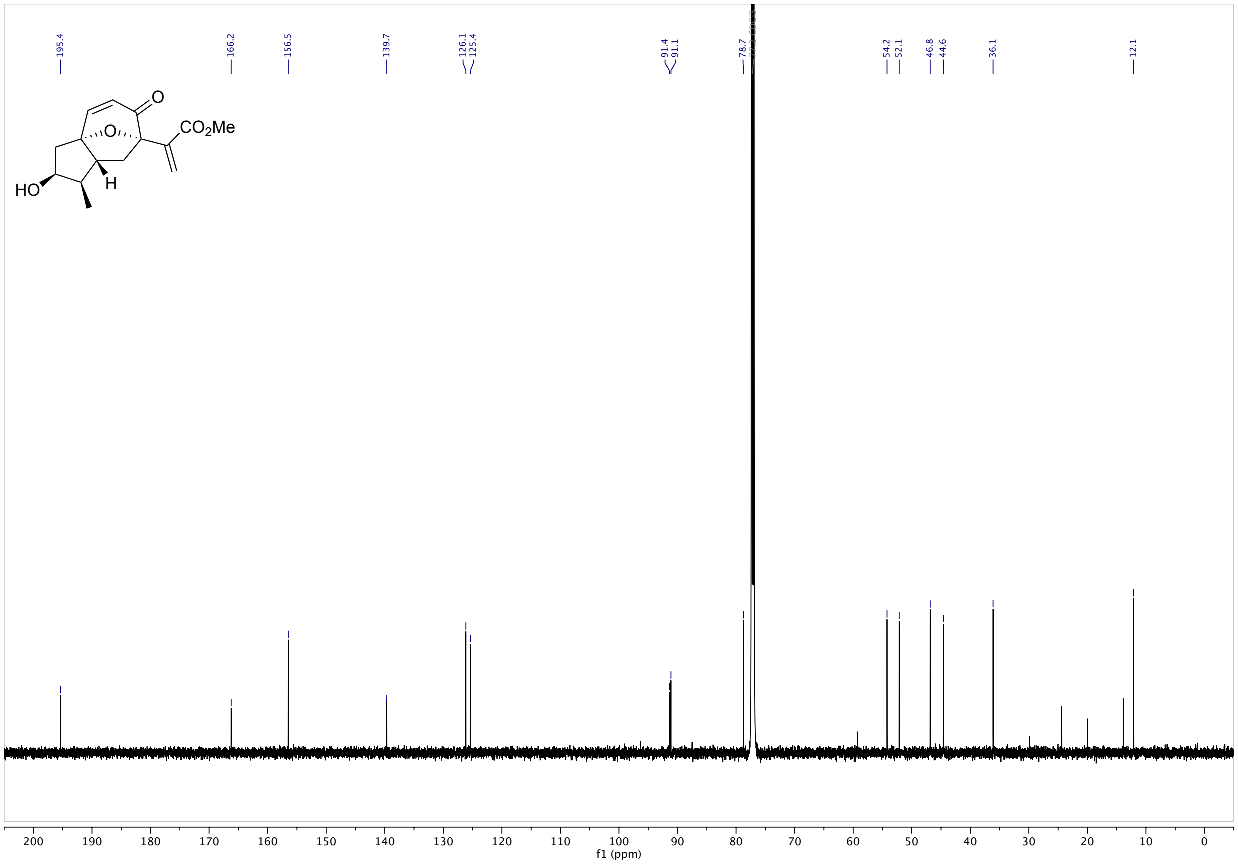

^{1}H NMR (400 MHz, $CDCl_3$) (32)

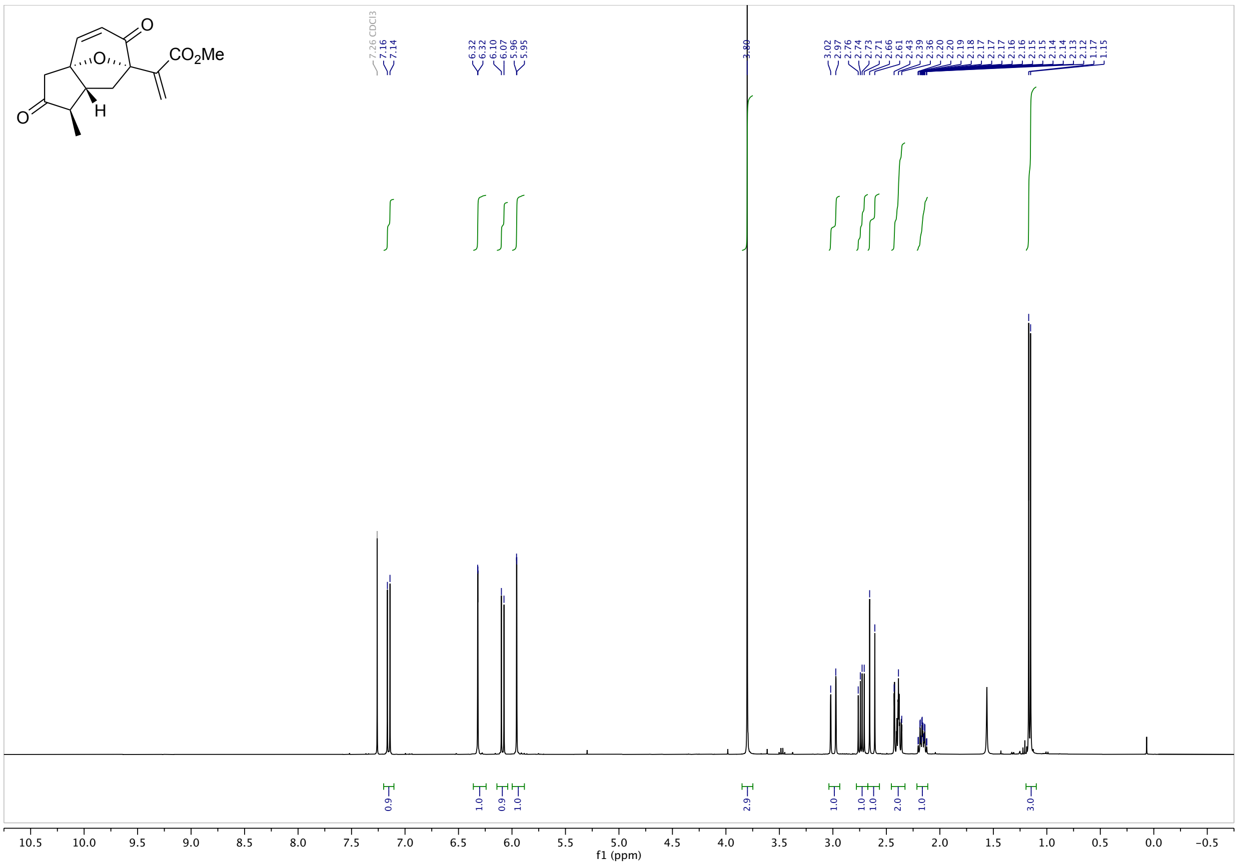

^{13}C NMR (101 MHz, $CDCl_3$) (32)

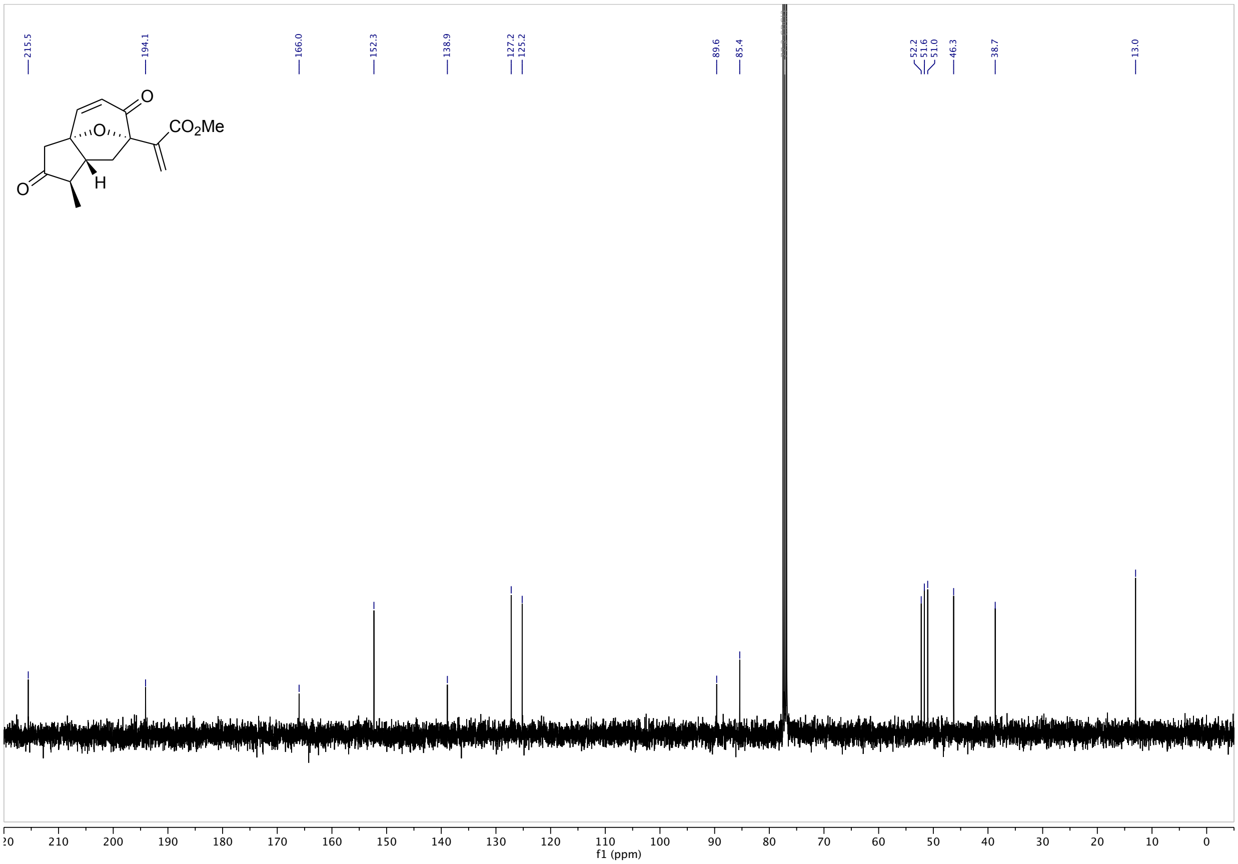

^{1}H NMR (400 MHz, C_6D_6) (S5)

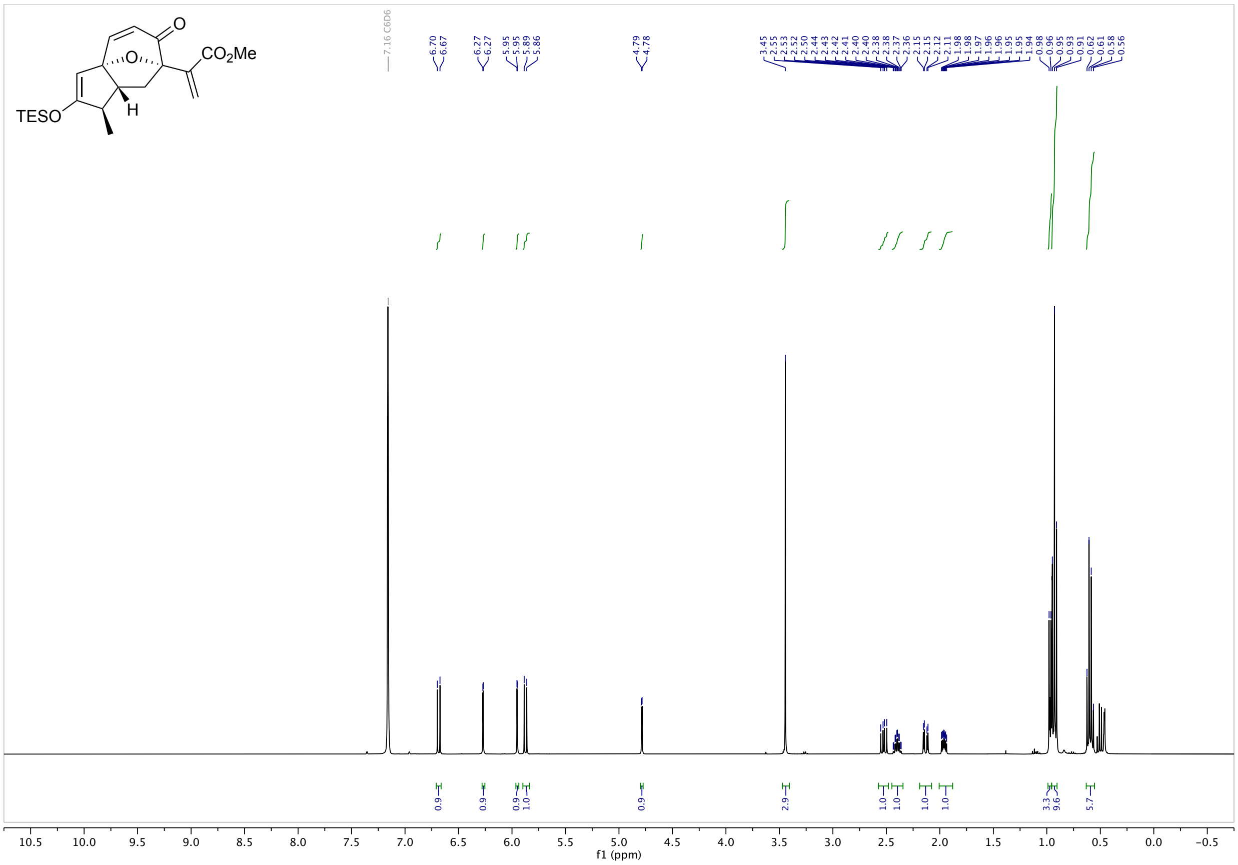

^{13}C NMR (101 MHz, C_6D_6) (S5)

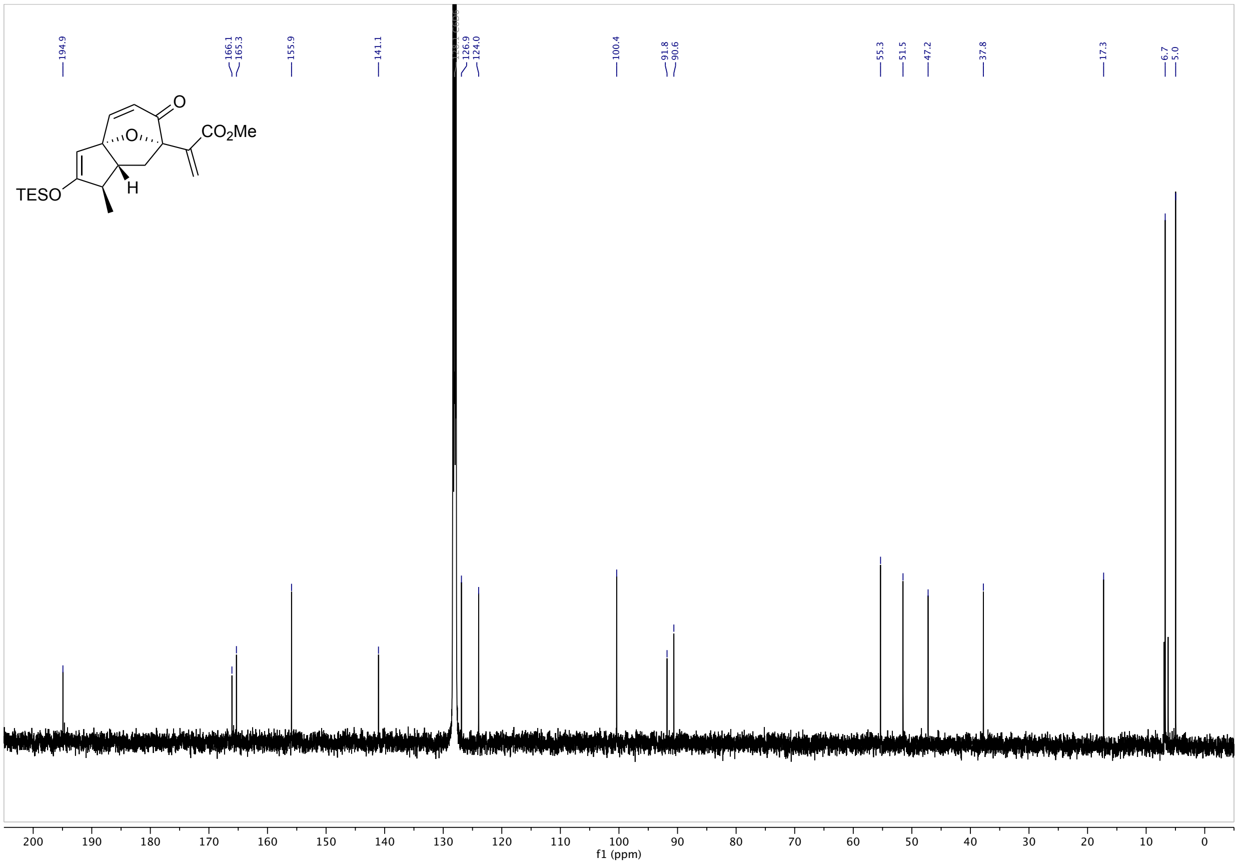

^{1}H NMR (400 MHz, C_6D_6) (33)

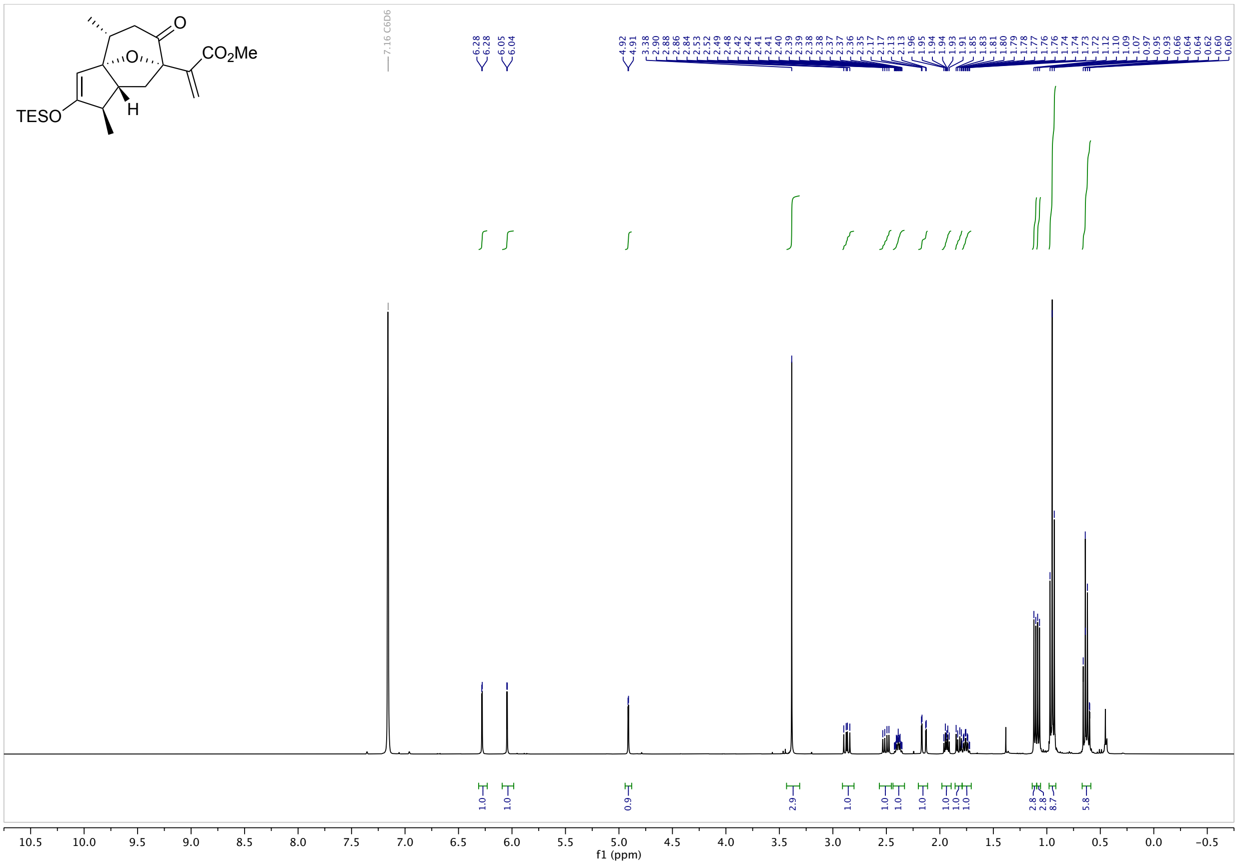

^{13}C NMR (101 MHz, C_6D_6) (33)

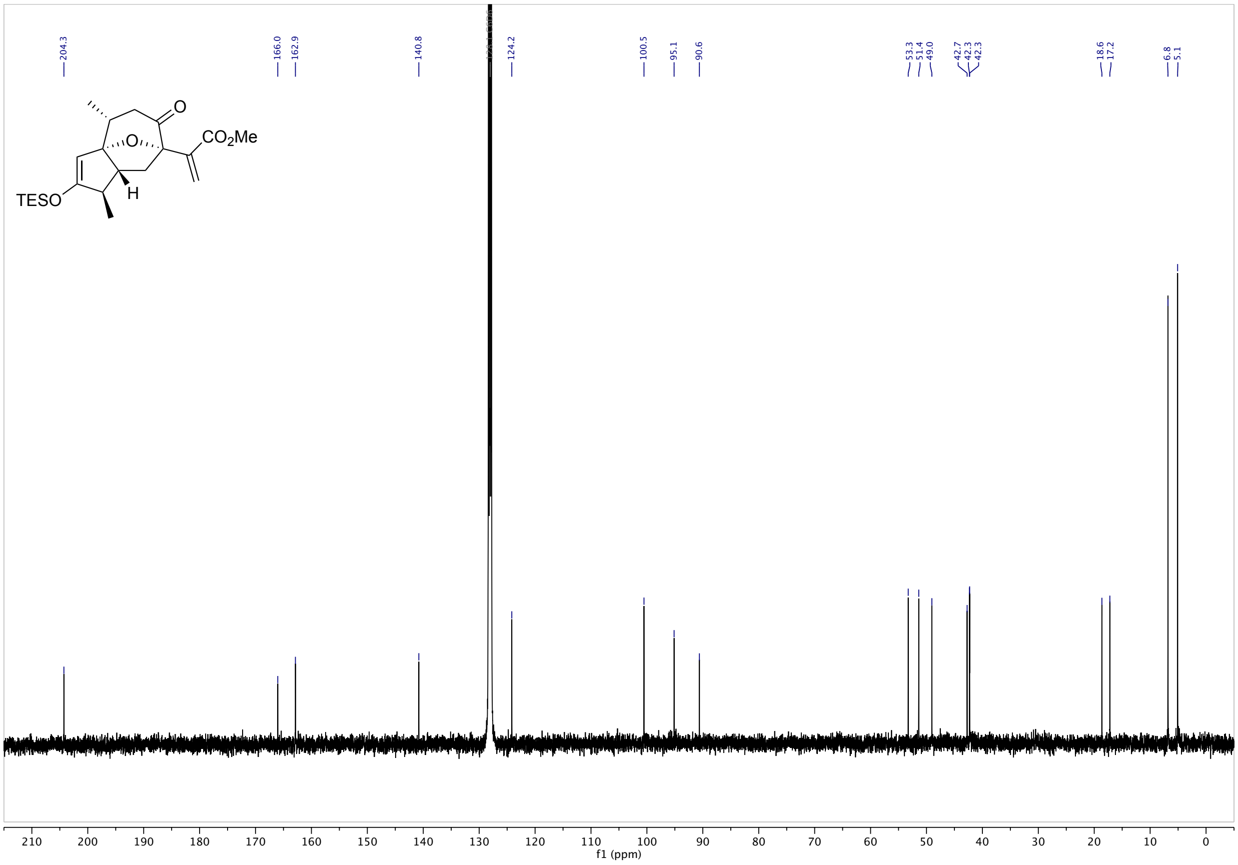

^{1}H NMR (600 MHz, $CDCl_3$) (34)

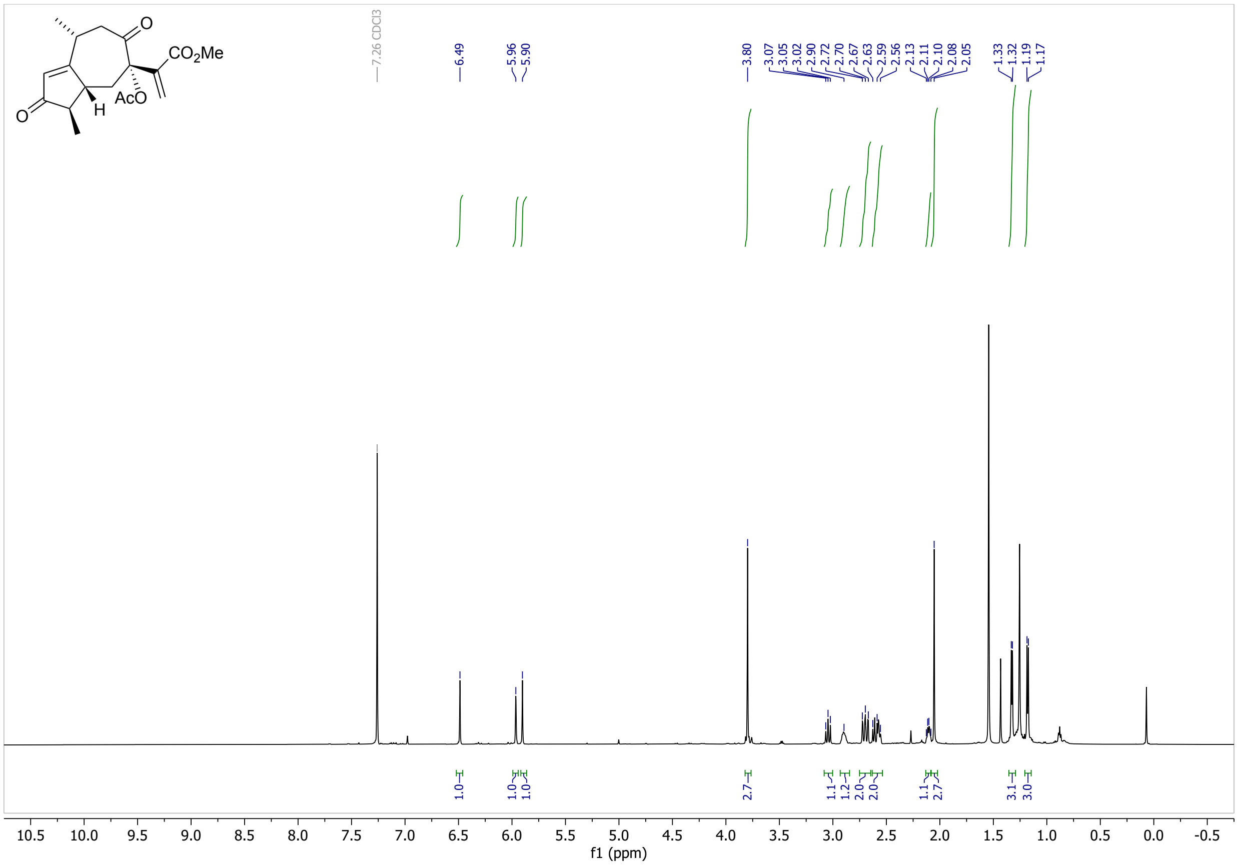

^{13}C NMR (151 MHz, $CDCl_3$) (34)

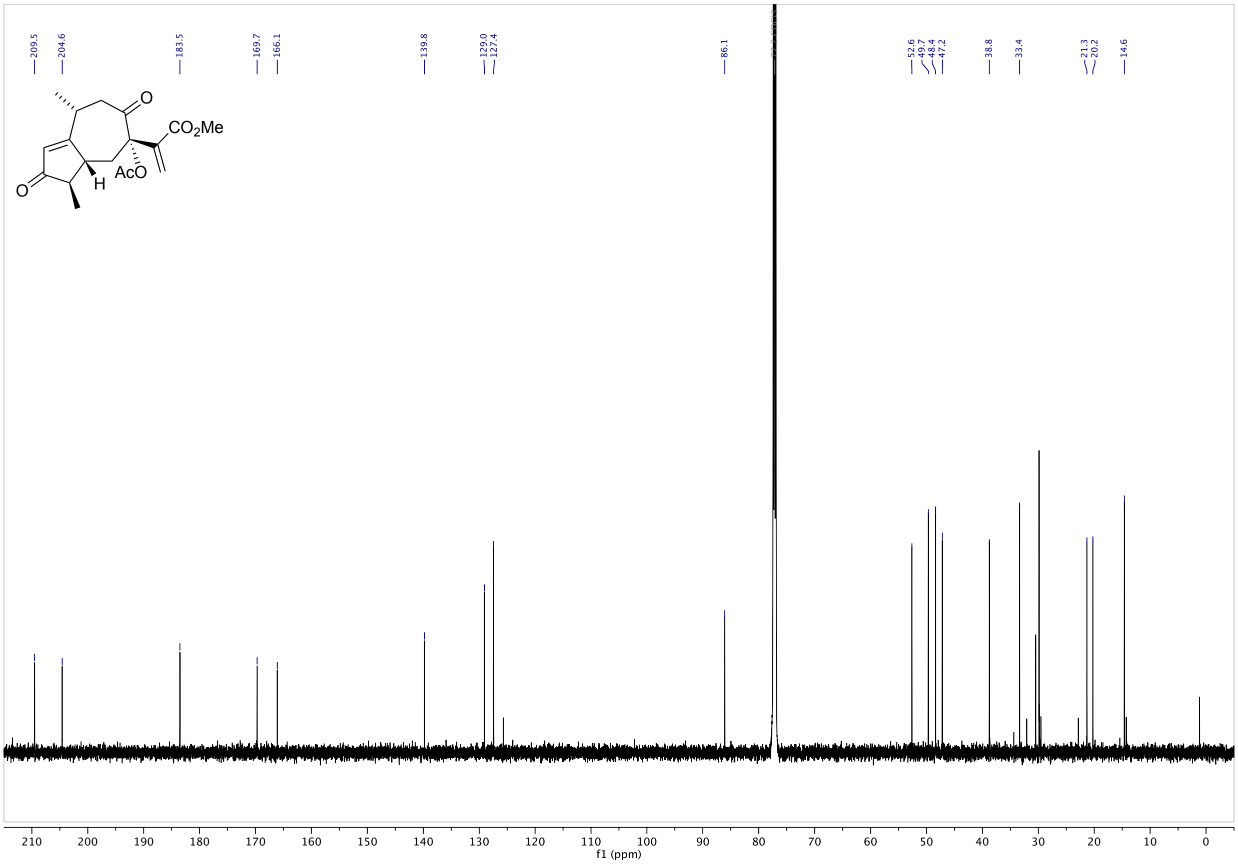

^{1}H NMR (400 MHz, C_6D_6) (S6)

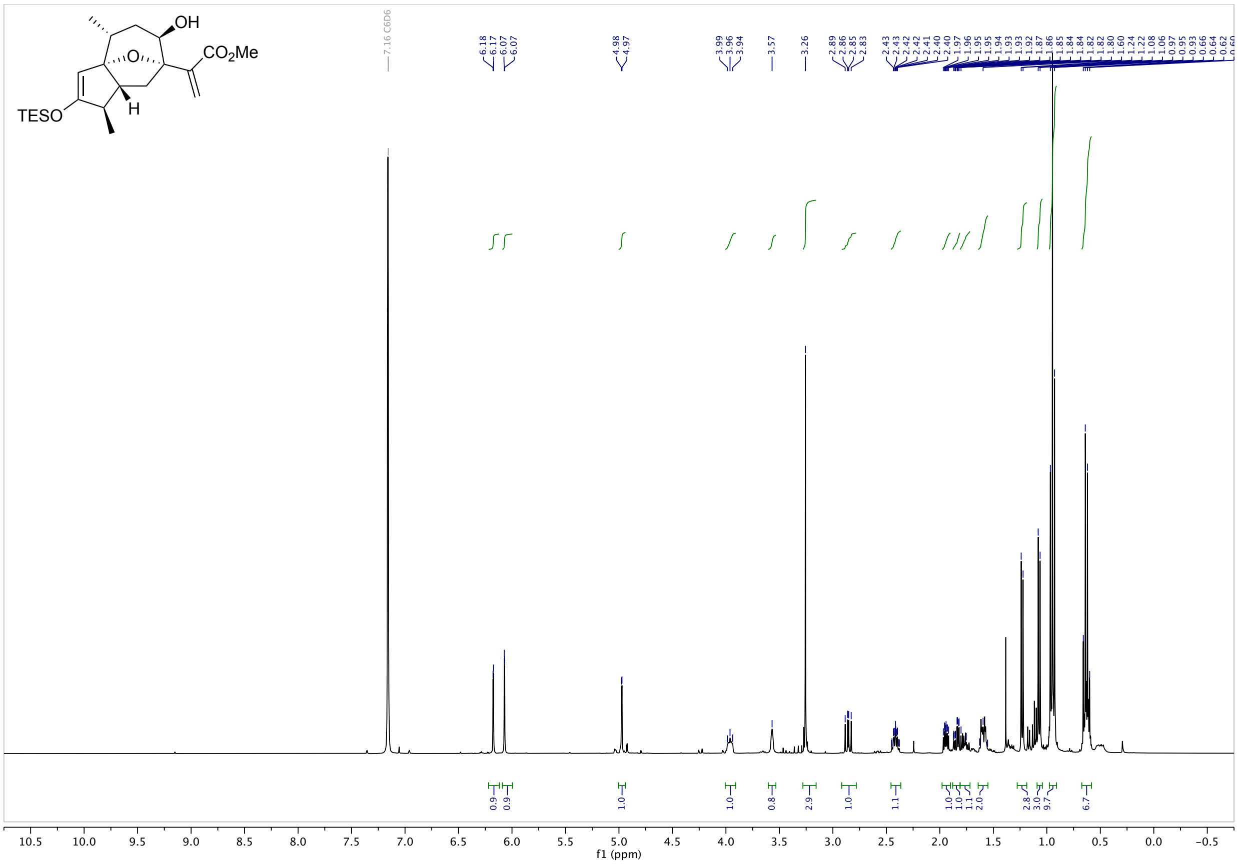

^{13}C NMR (101 MHz, C_6D_6) (S6)

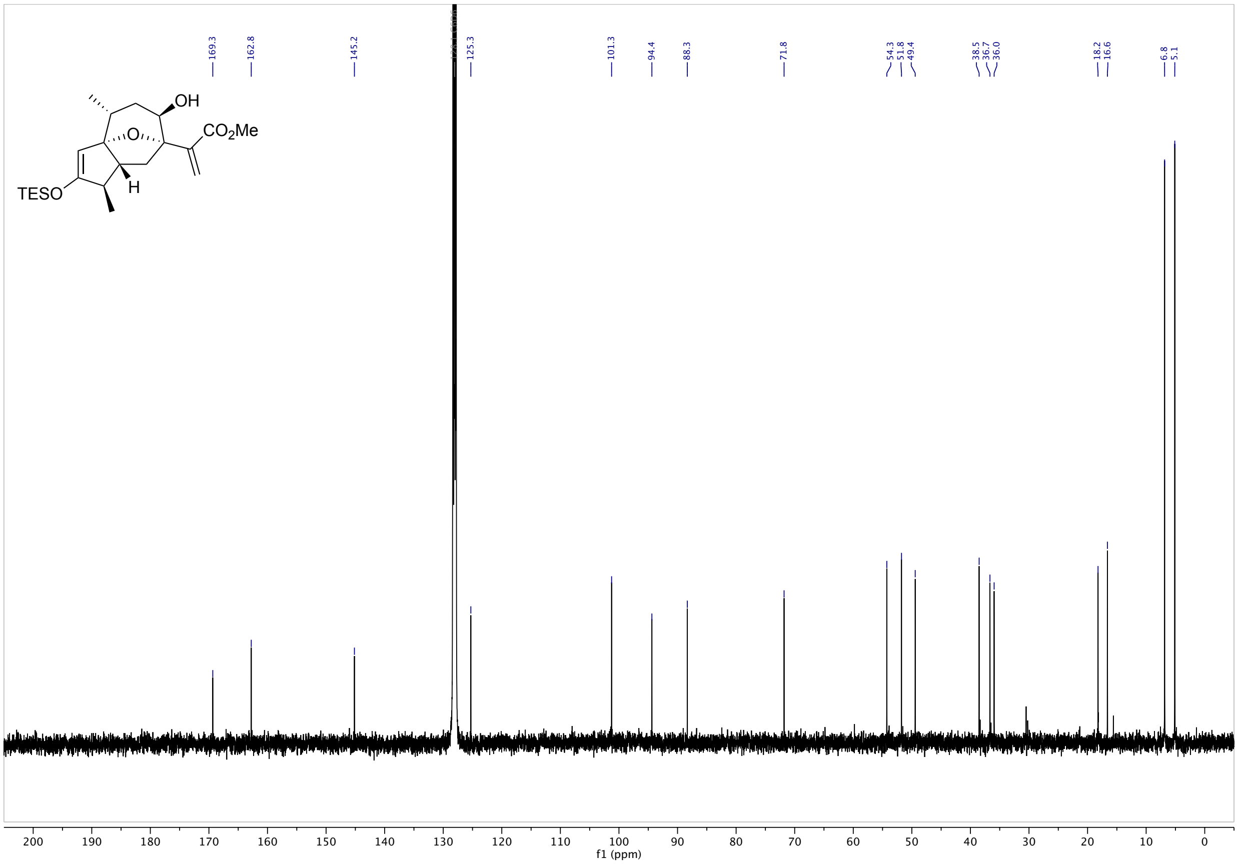

^{1}H NMR (500 MHz, C_6D_6) (35)

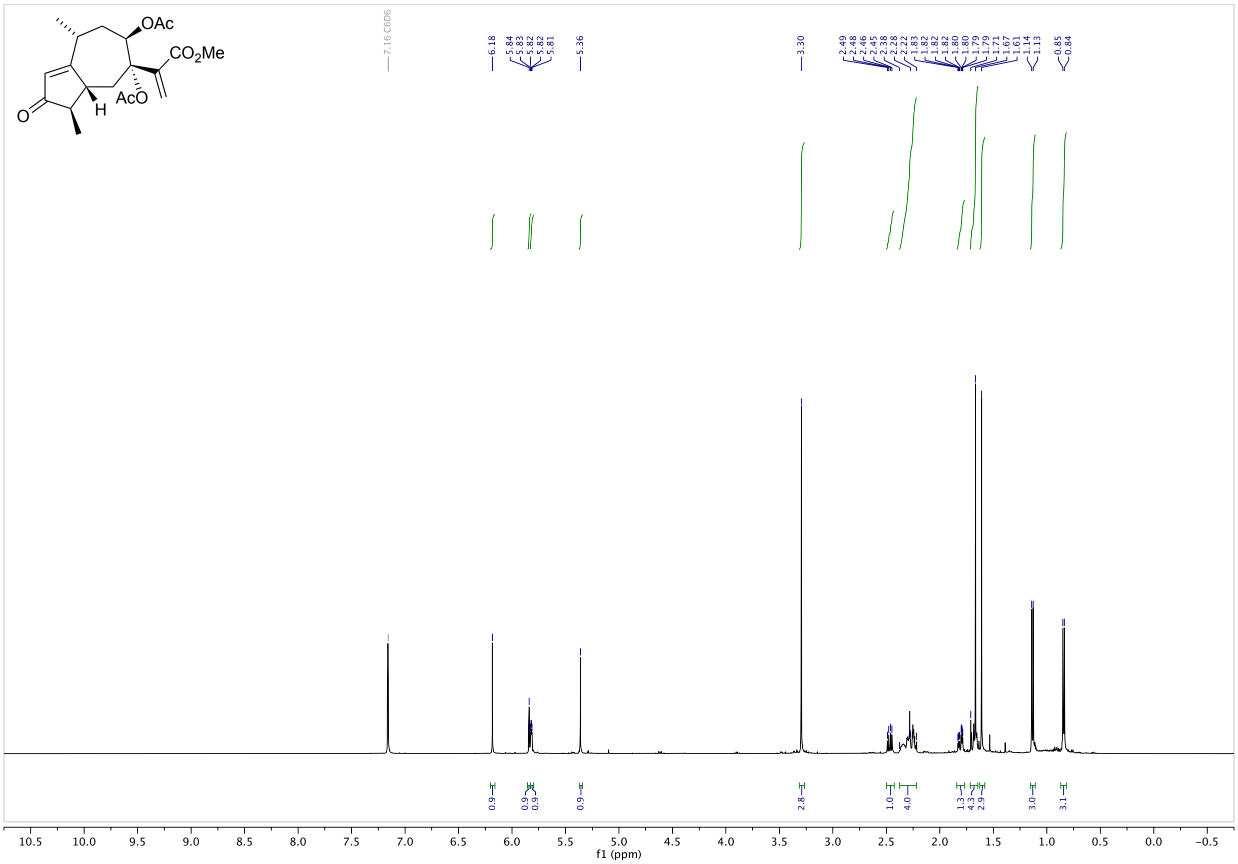

^{13}C NMR (126 MHz, C_6D_6) (35)

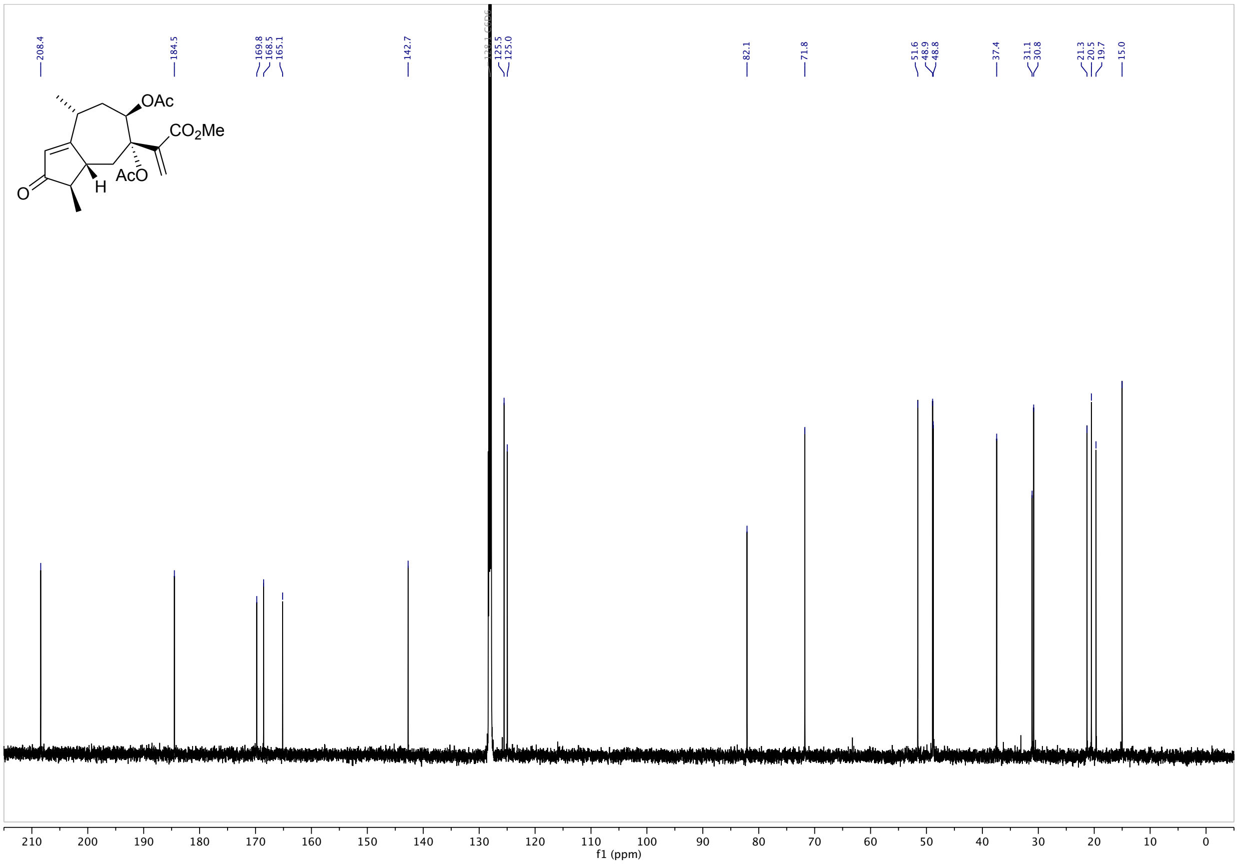

^{1}H NMR (700 MHz, C_6D_6) **(36)** – crude

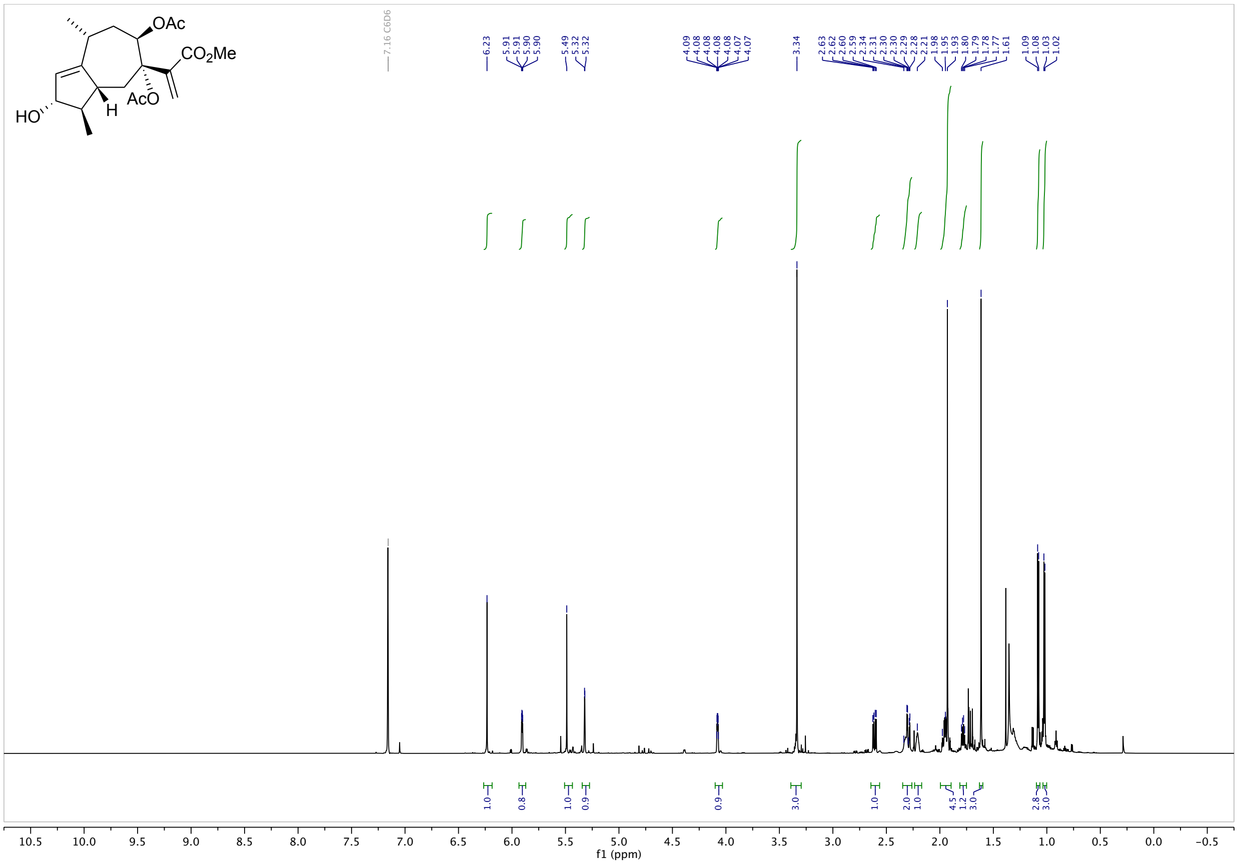

^{13}C NMR (176 MHz, C_6D_6) **(36)** – crude

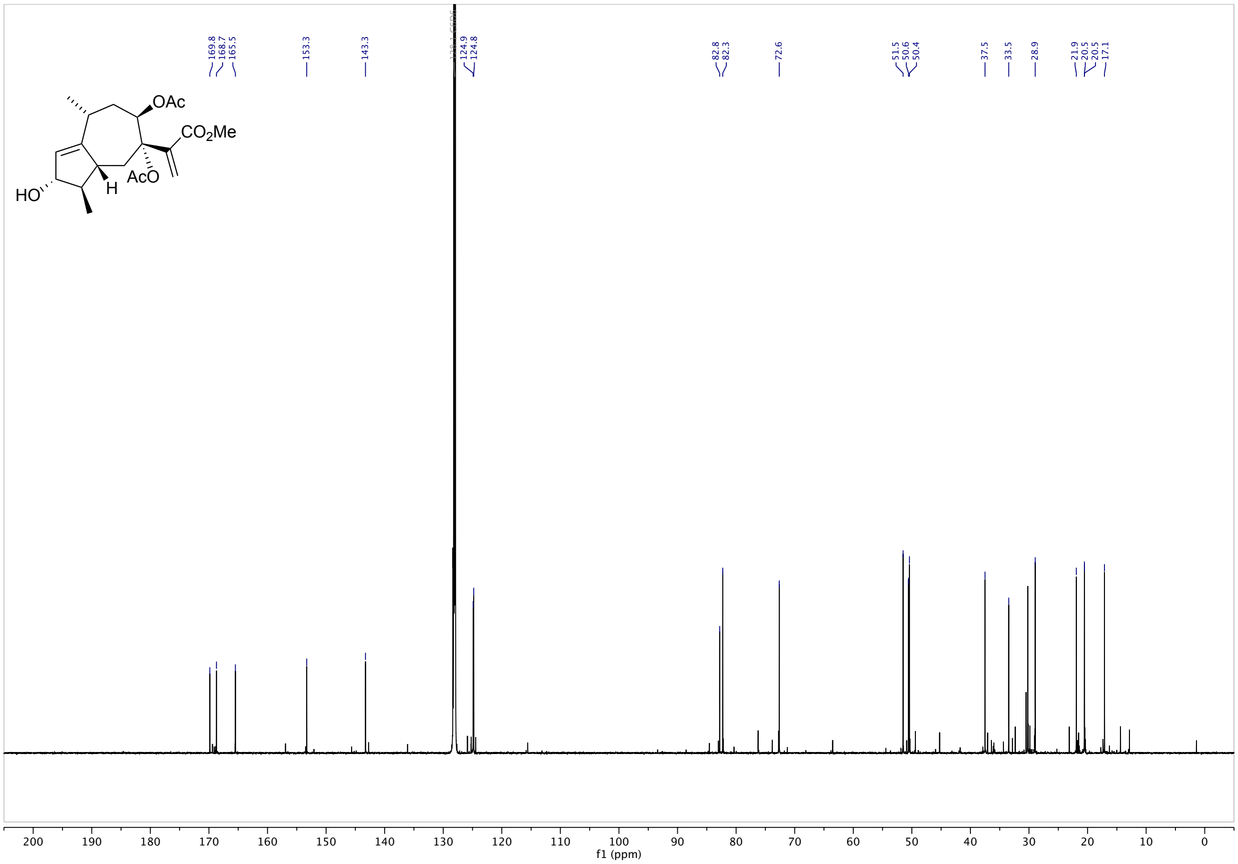

^{1}H NMR (500 MHz, C_6D_6) **(37)** (+ minor regioisomer)

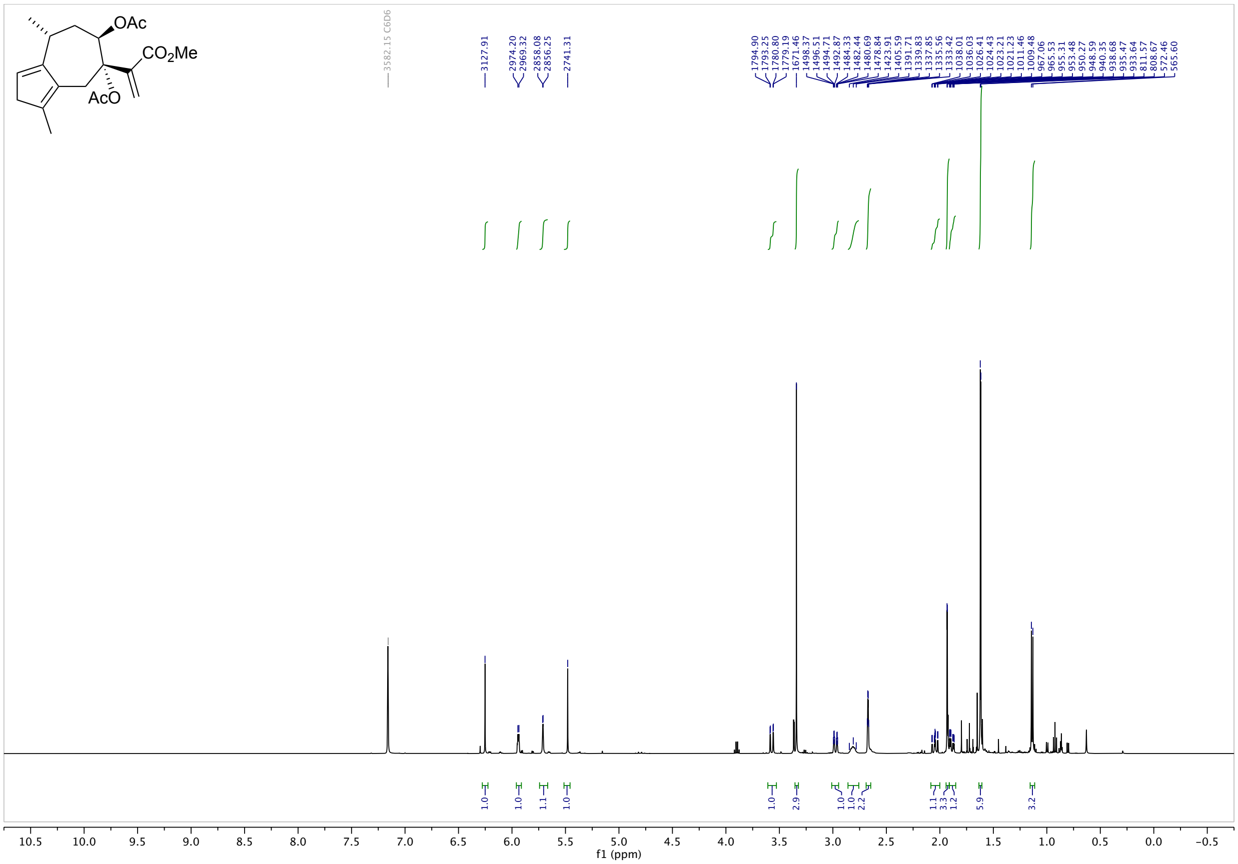

^{13}C NMR (126 MHz, C_6D_6) **(37)** (+ minor regioisomer)

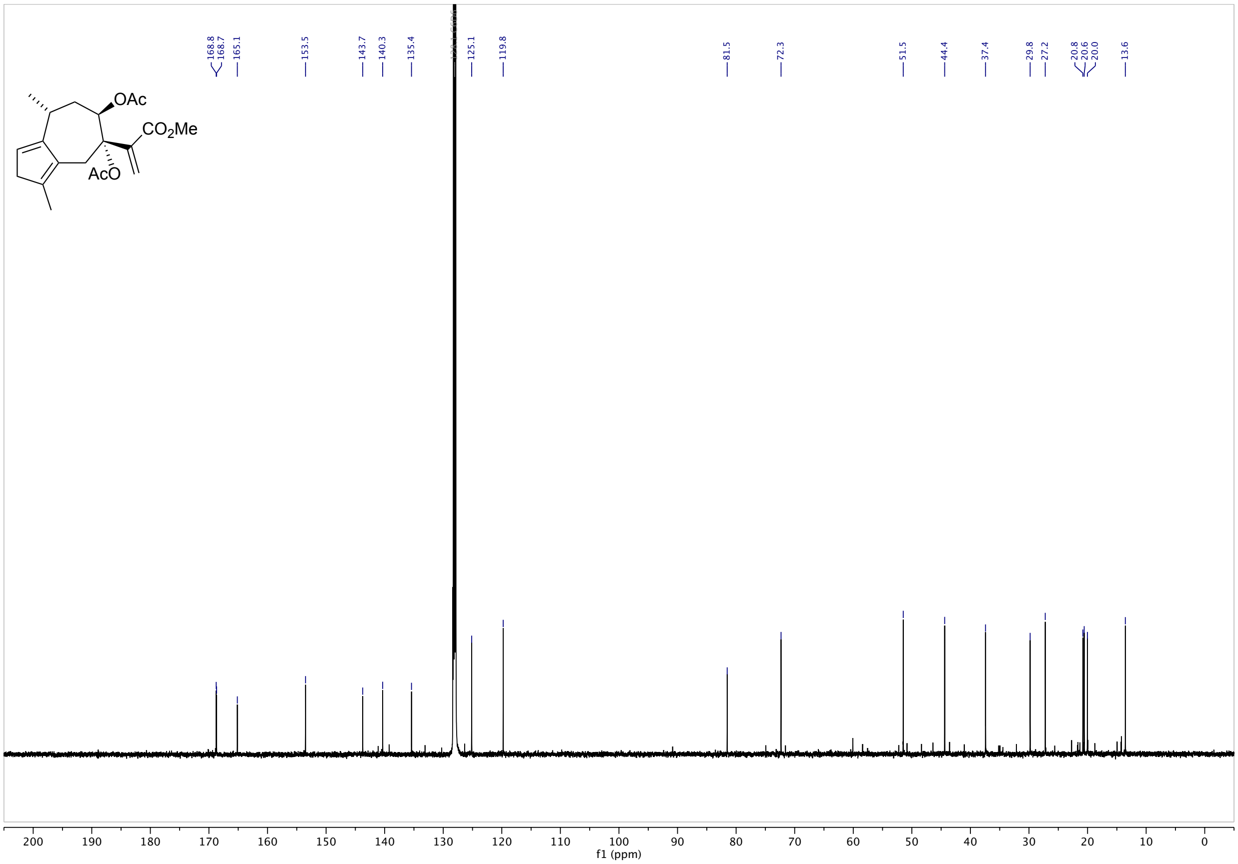

^{1}H NMR (700 MHz, C_6D_6) (38)

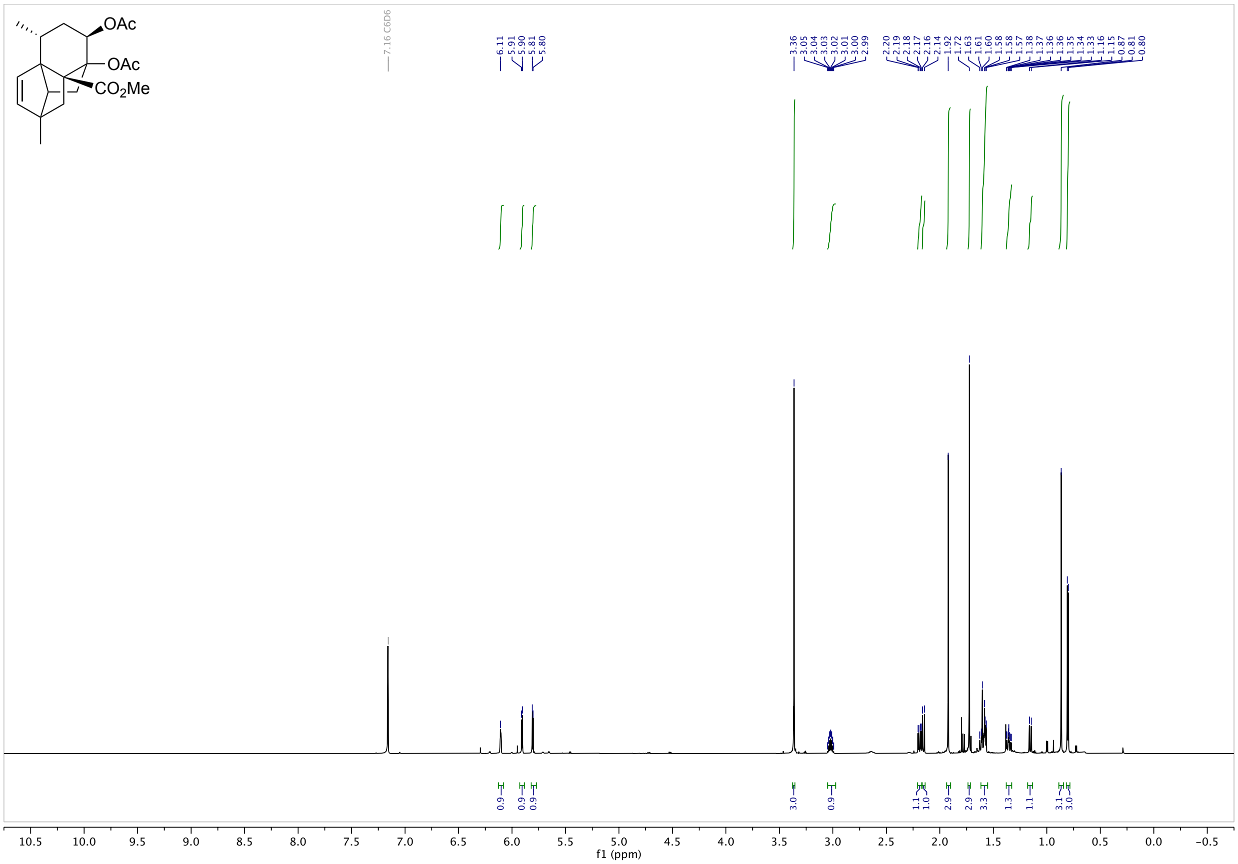

^{13}C NMR (176 MHz, C_6D_6) (38)

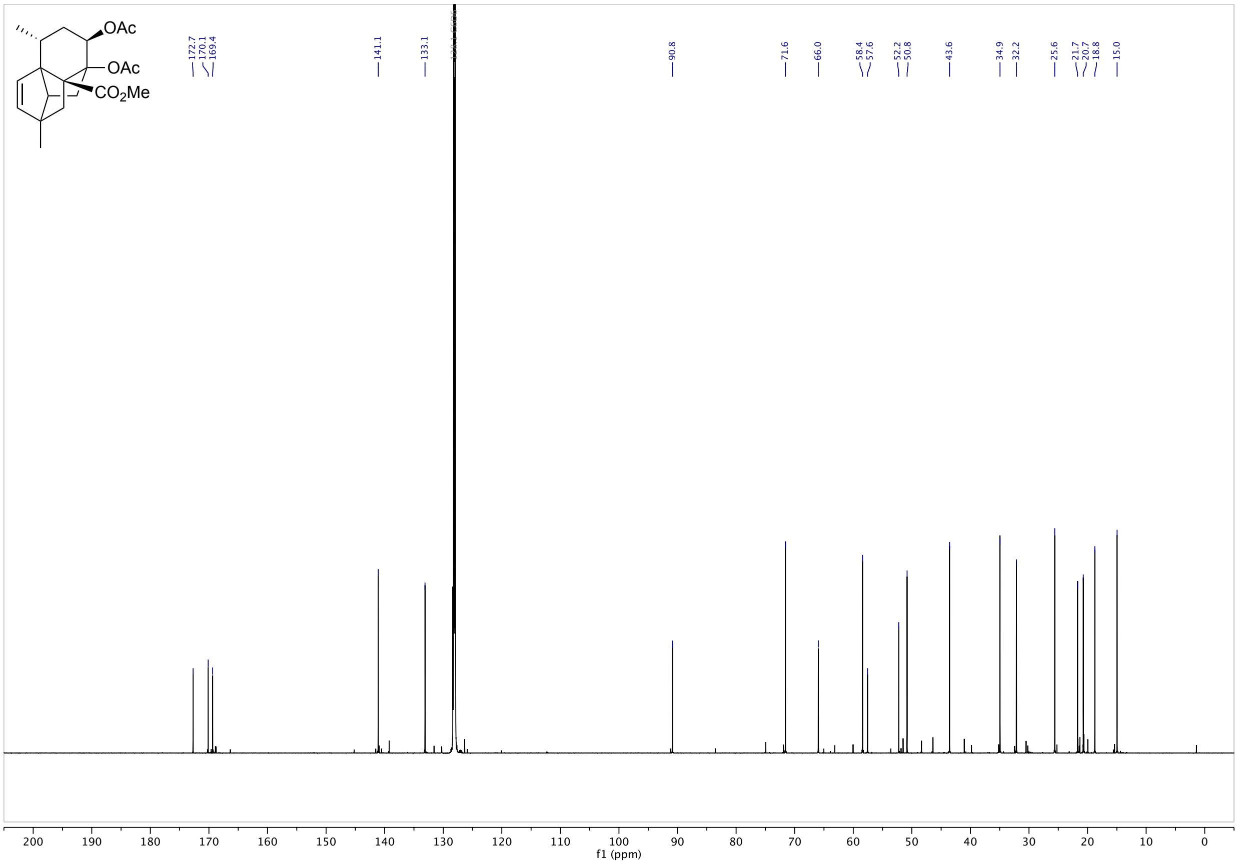

^{1}H NMR (700 MHz, C_6D_6) (39)

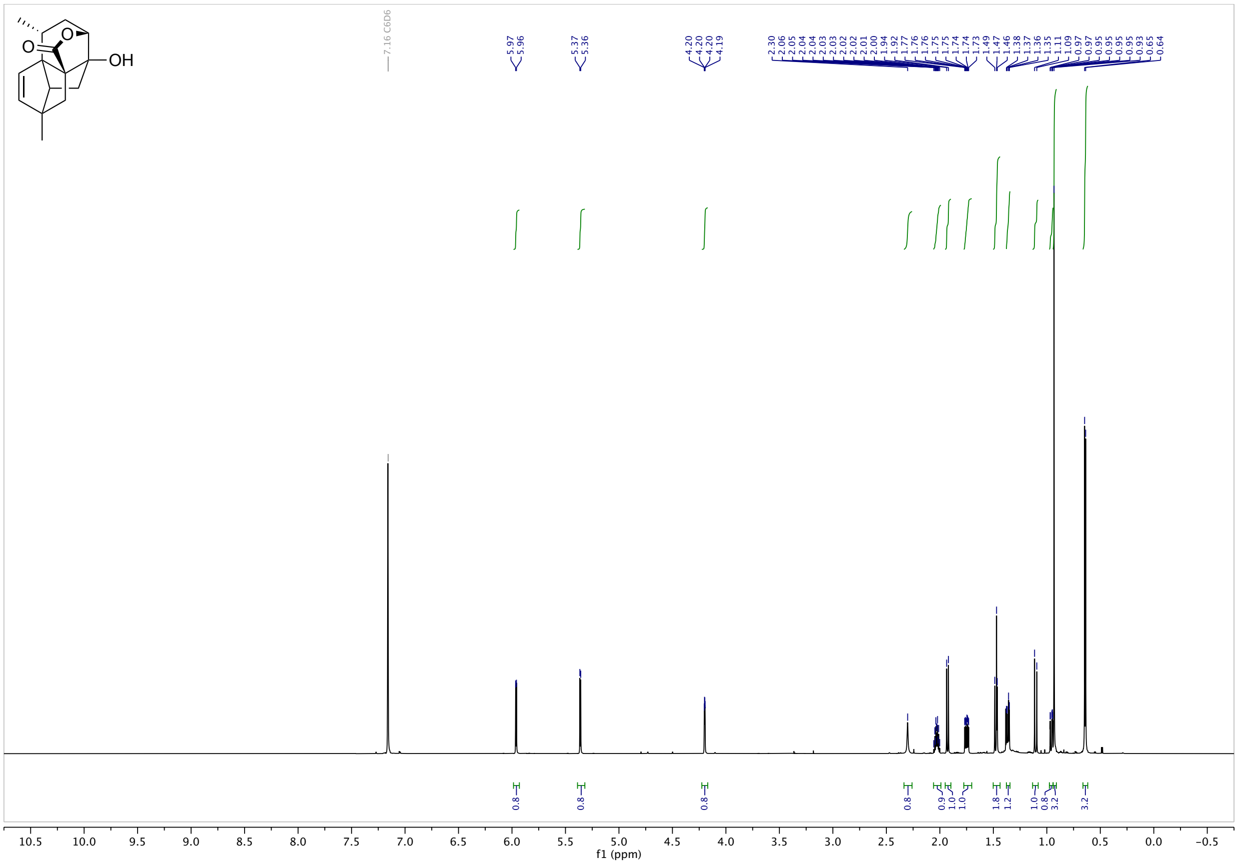

^{13}C NMR (176 MHz, C_6D_6) (39)

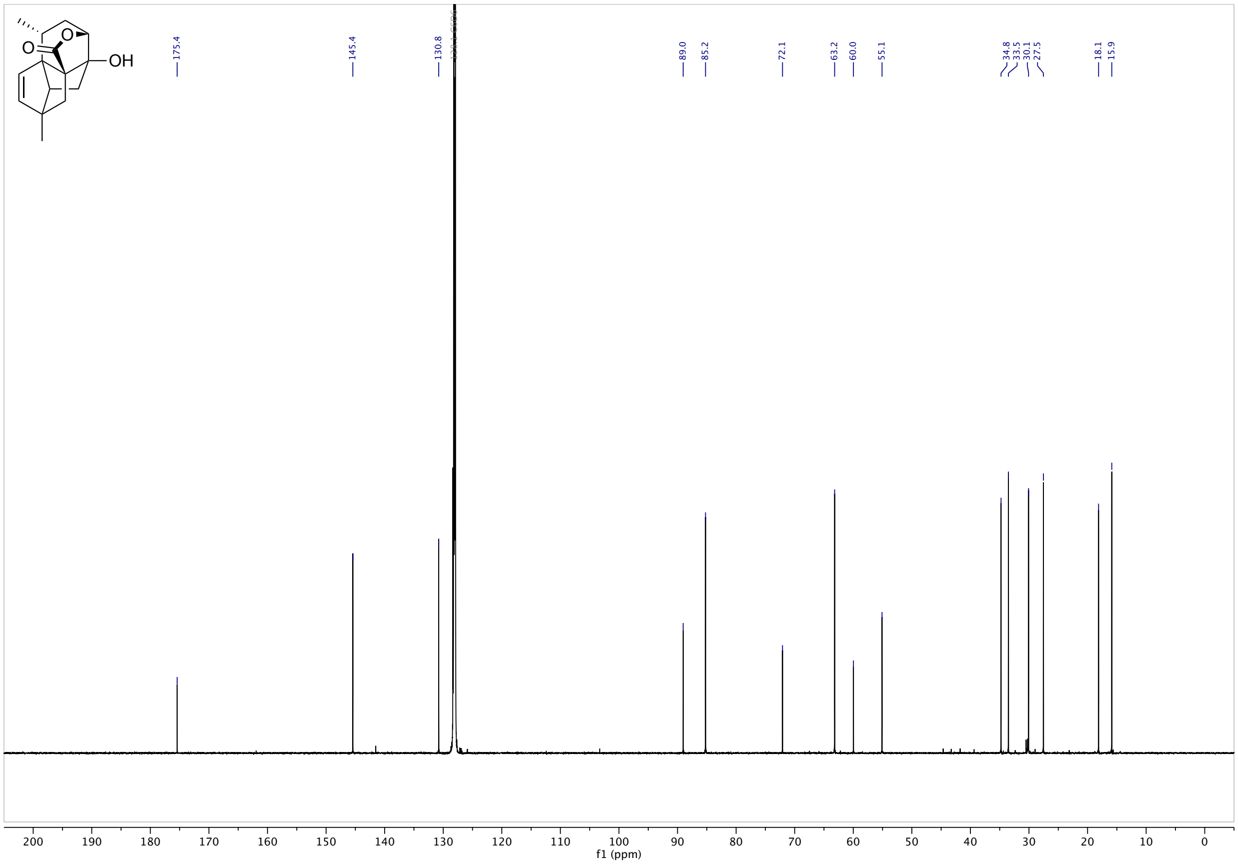

^{1}H–^{1}H COSY (700 MHz, C_6D_6) (39)

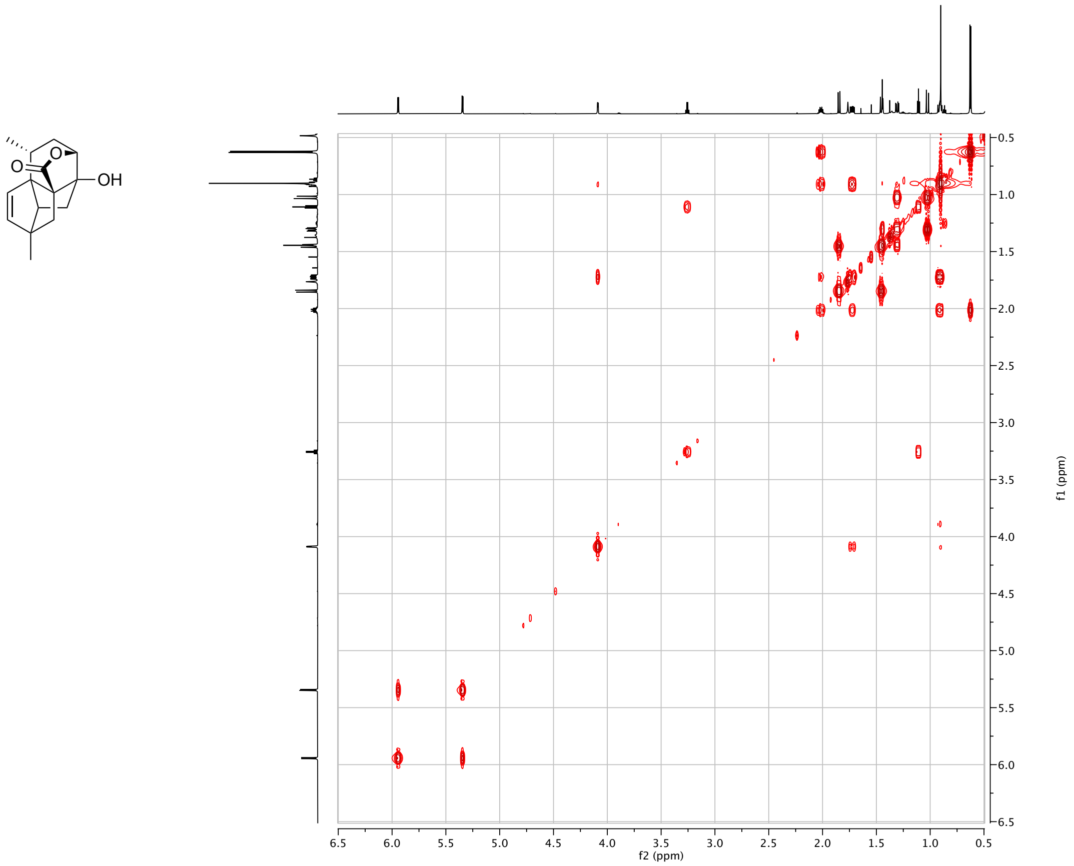

^{1}H–^{13}C HSQC (C_6D_6) (39)

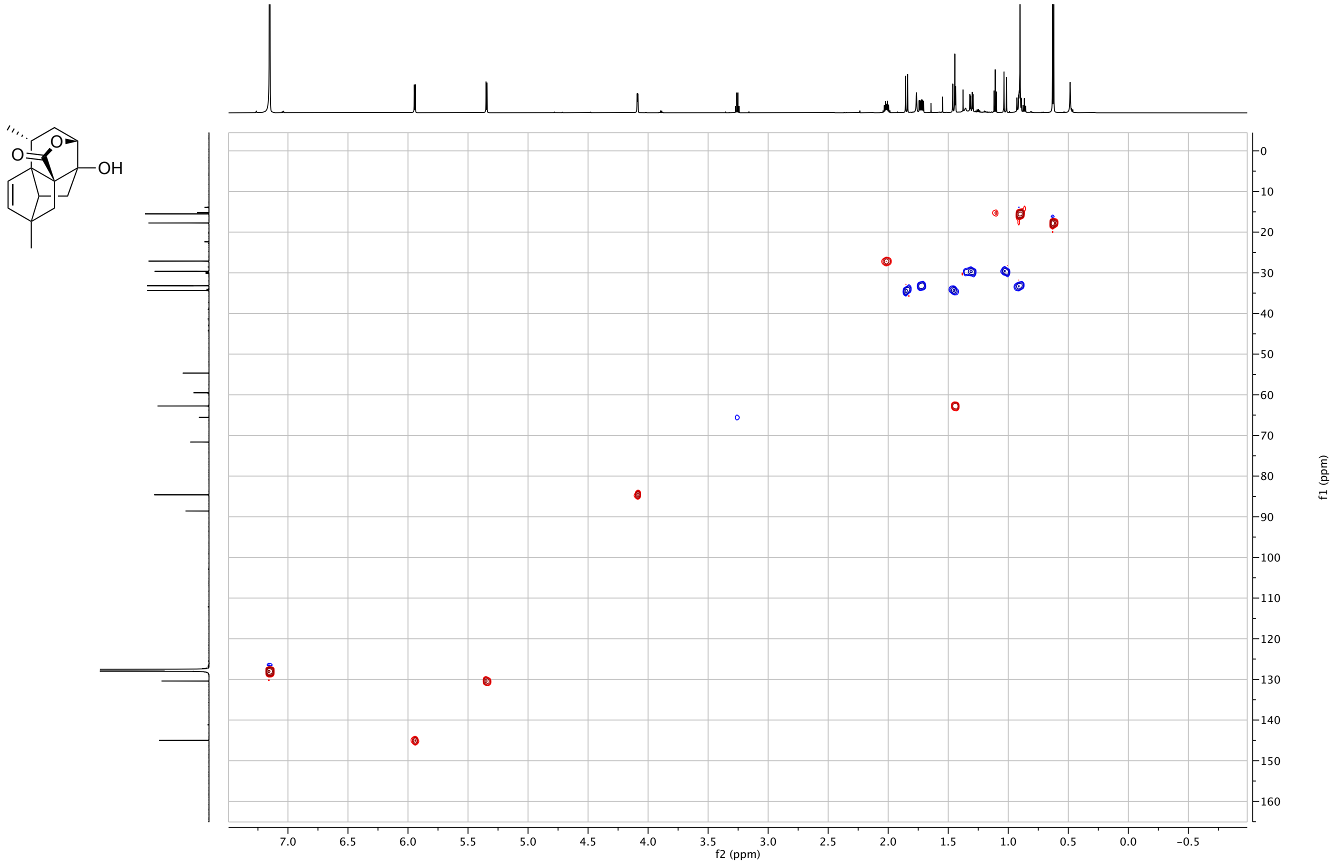

^1H–^{13}C HMBC (C_6D_6) (39)

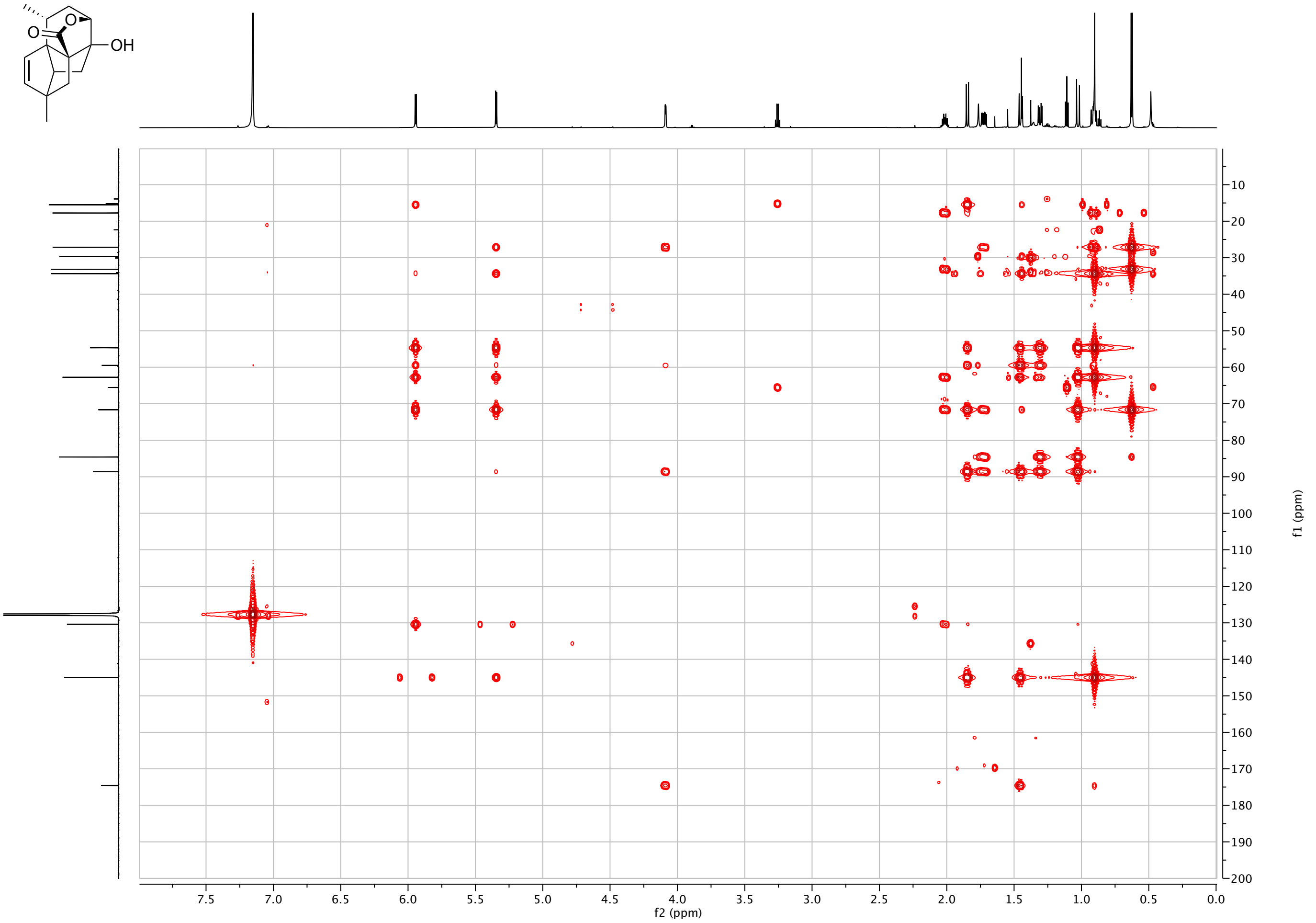

^{1}H NMR (600 MHz, C_6D_6) (40)

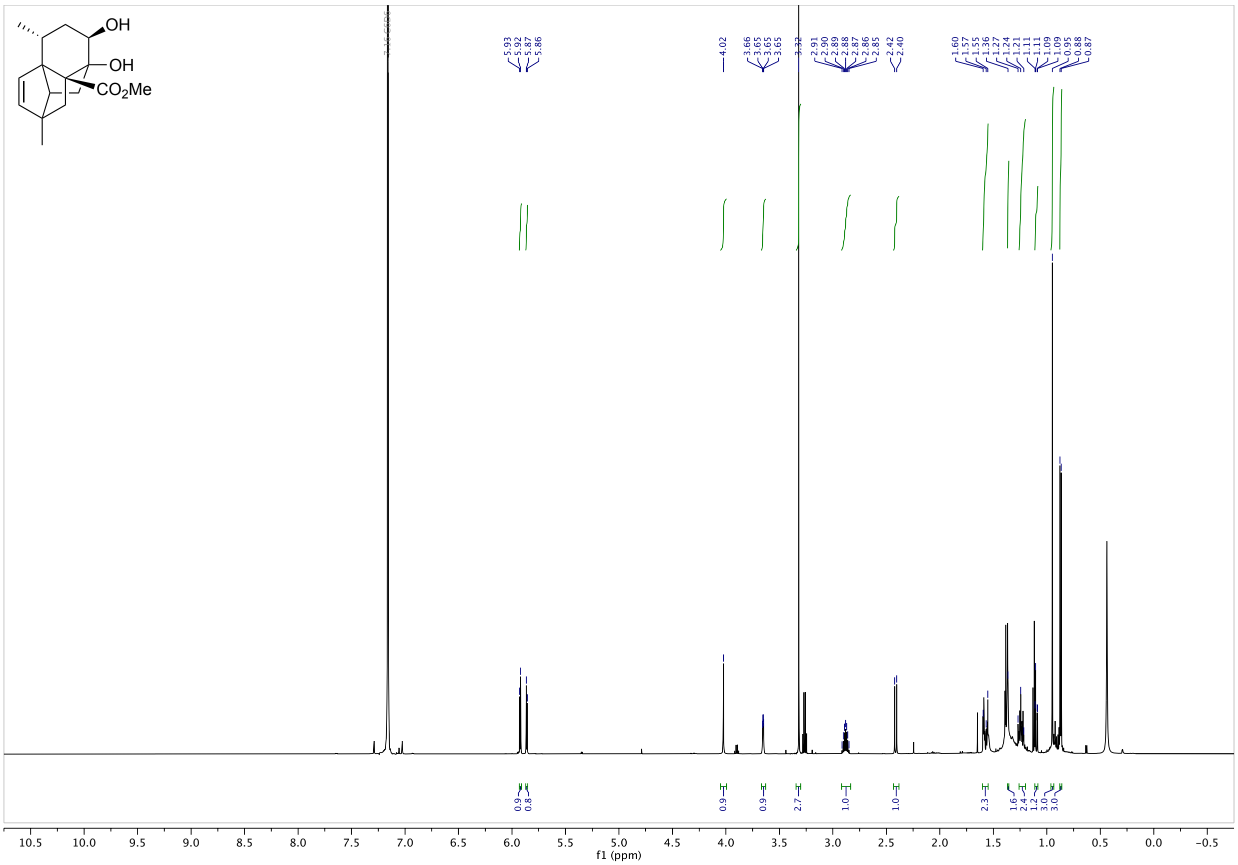

^{13}C NMR (151 MHz, C_6D_6) (40)

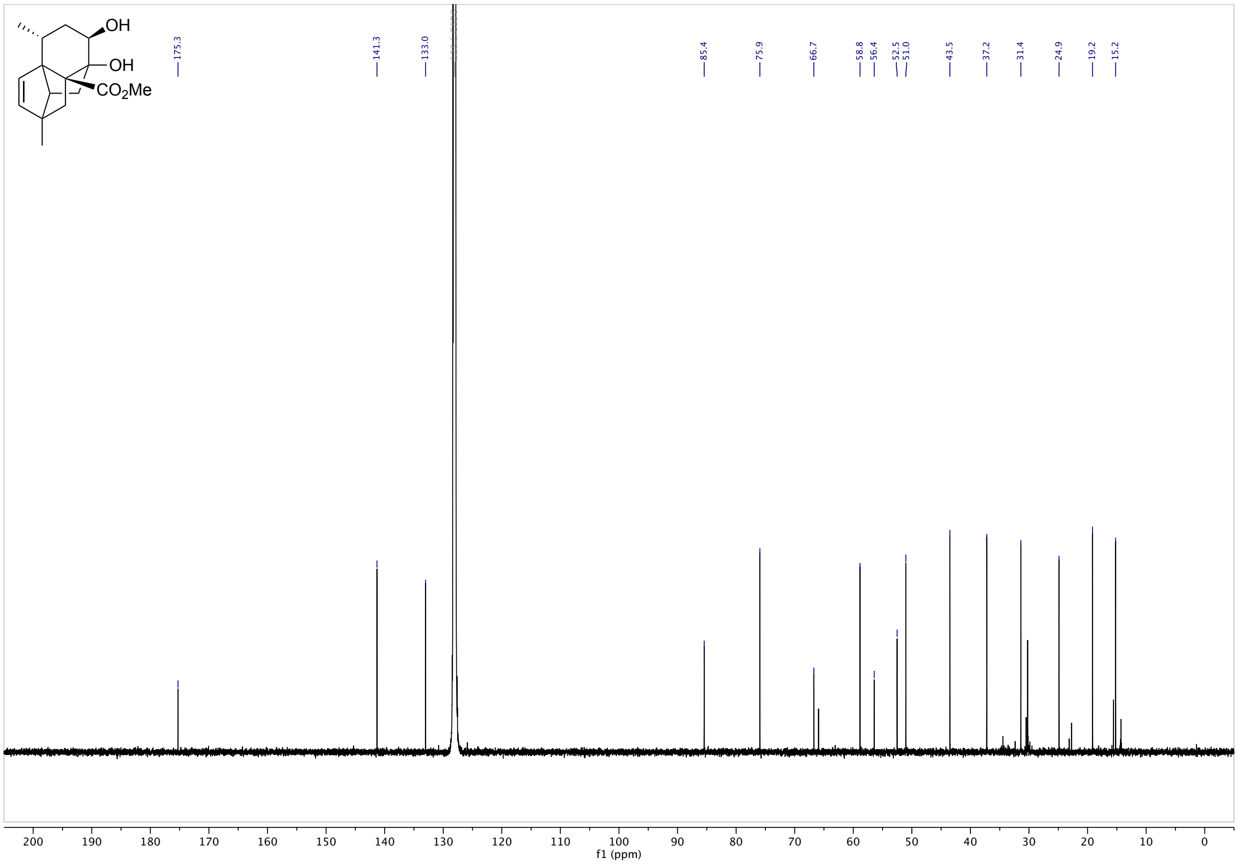

^{1}H NMR (500 MHz, C_6D_6) (41)

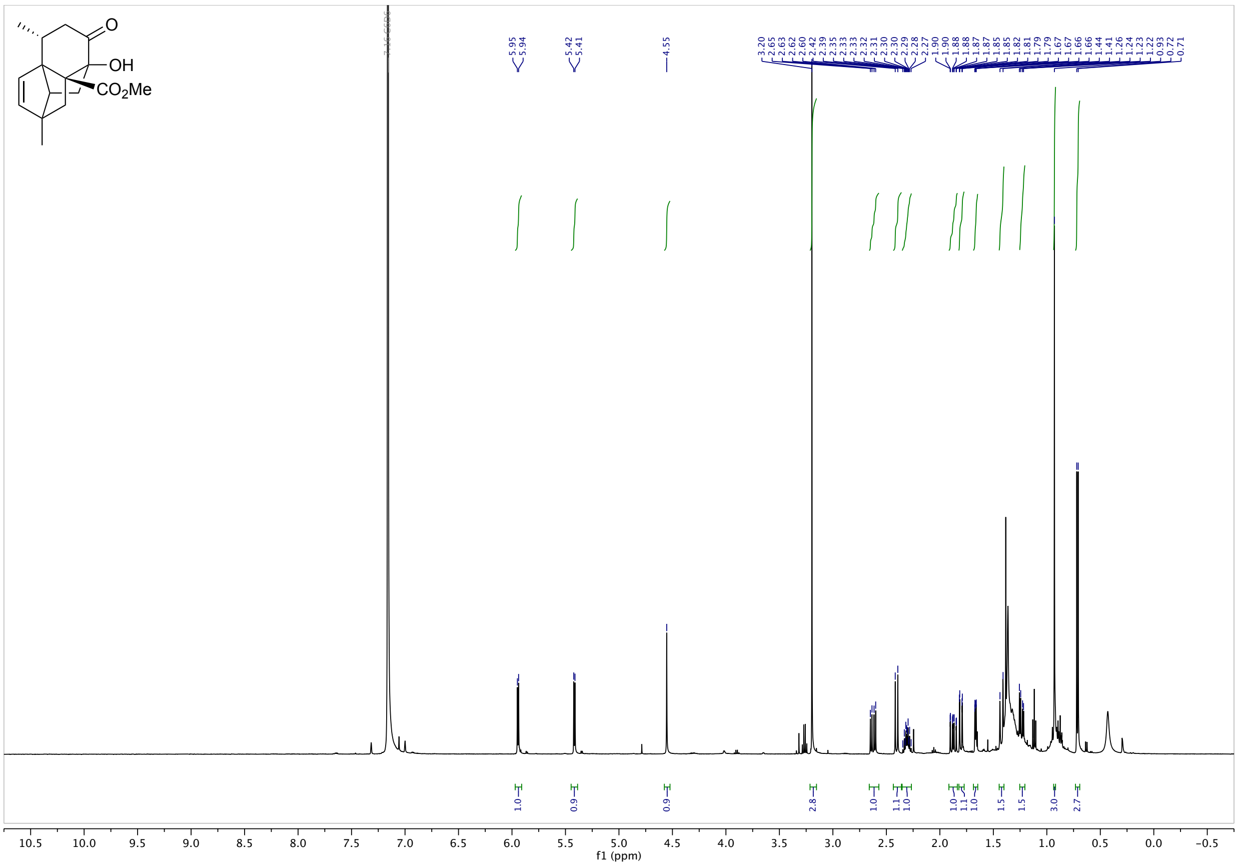

^{13}C NMR (126 MHz, C_6D_6) (41)

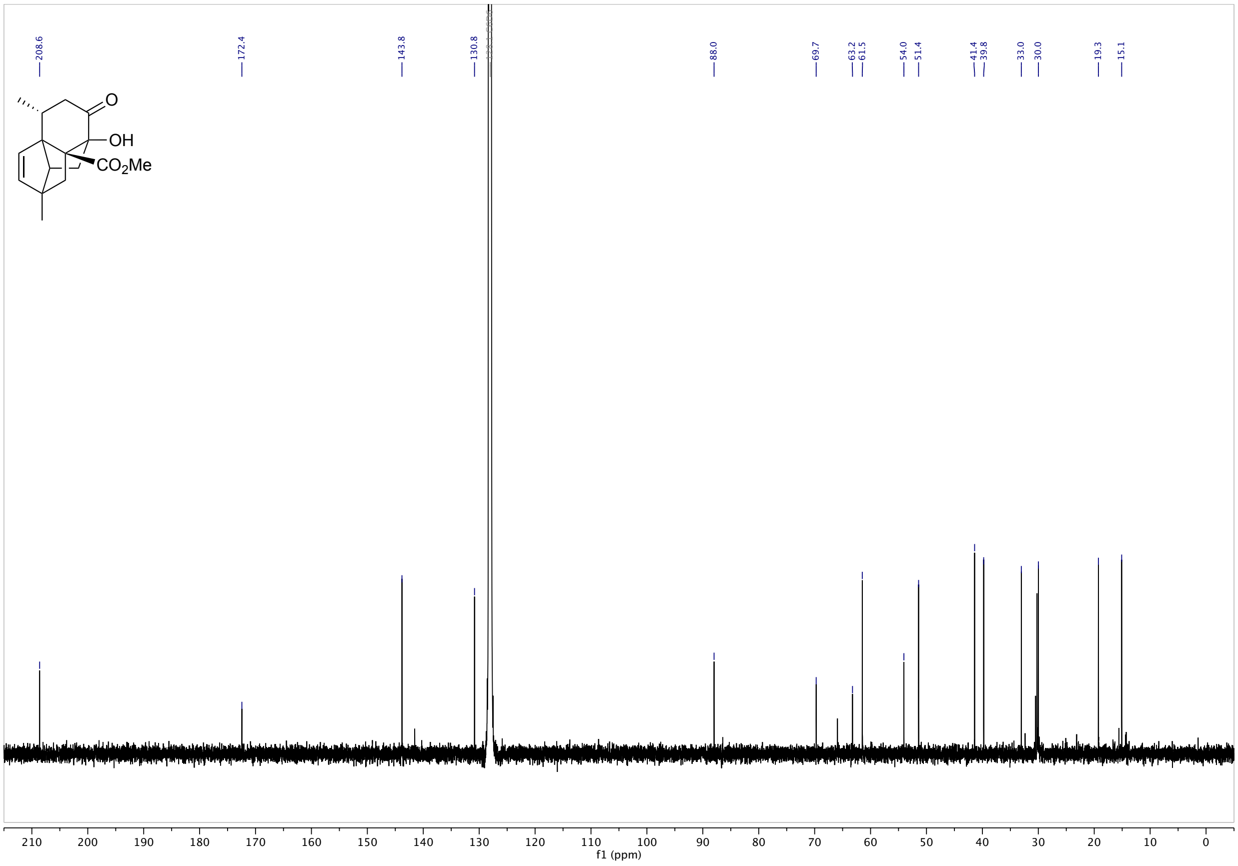

^{1}H NMR (600 MHz, $CDCl_3$) (42)

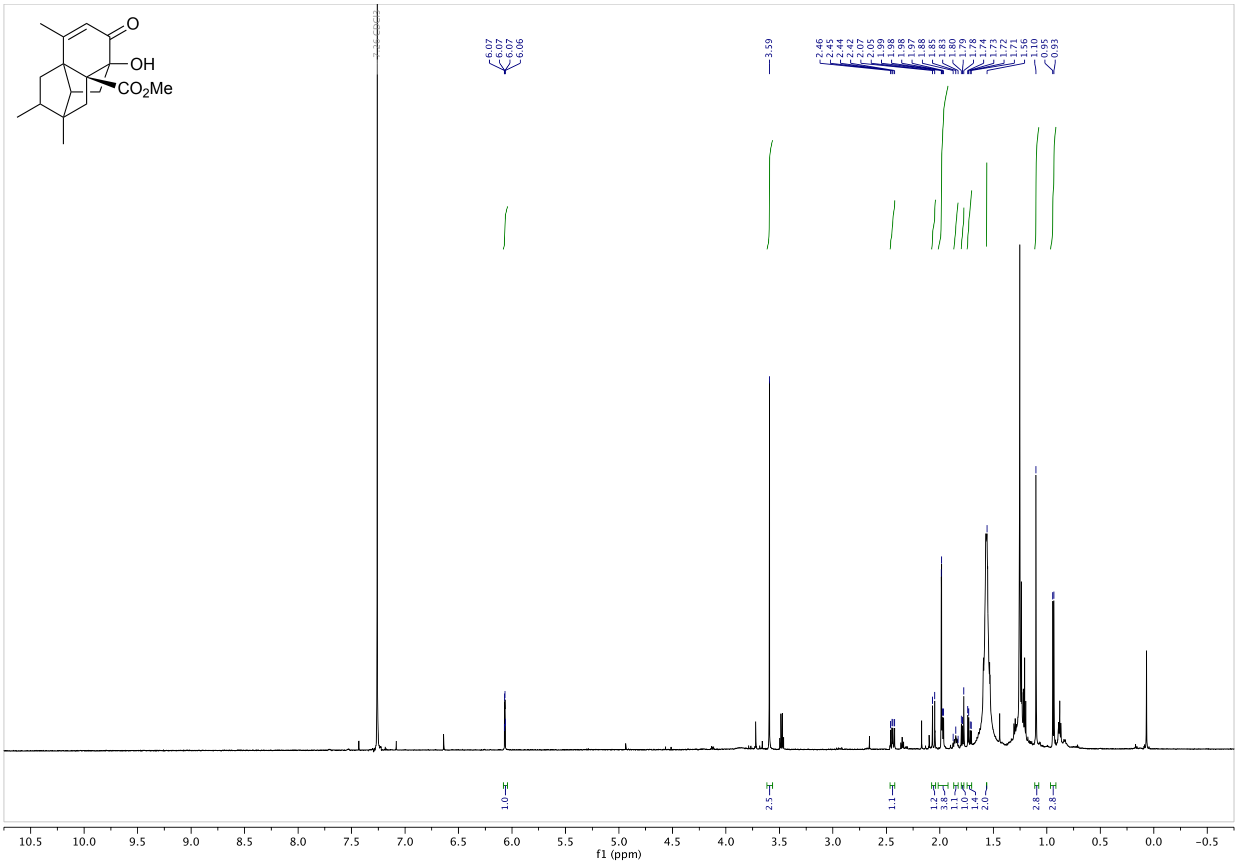

^{13}C NMR (151 MHz, $CDCl_3$) (42)

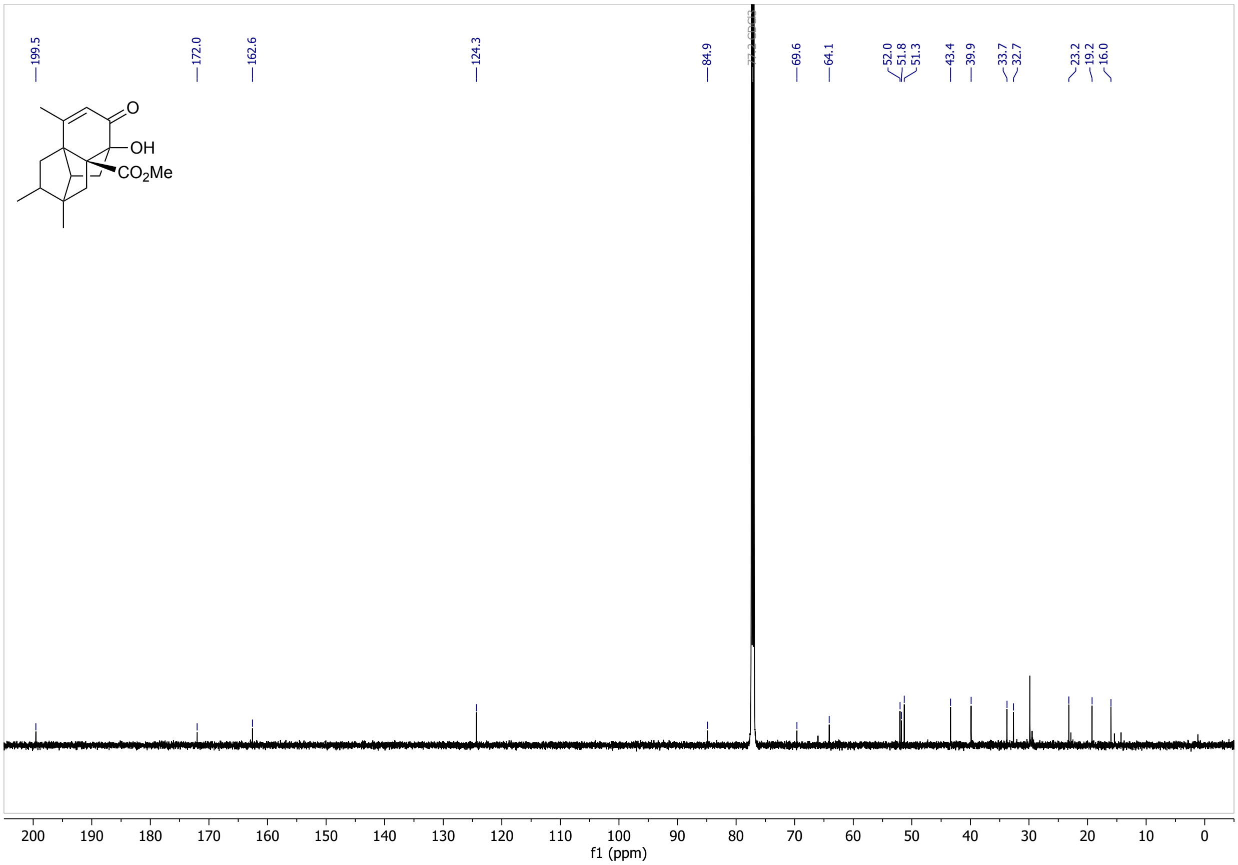

Diagnostic NOESY data for minor oxidopyrylium cycloadduct 28 (600 MHz, CDCl$_3$)

(red – α-face; green – β-face)

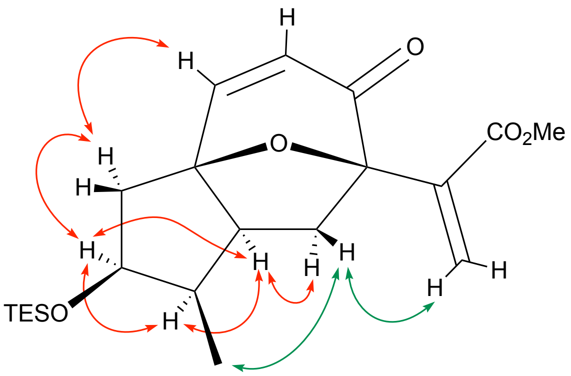

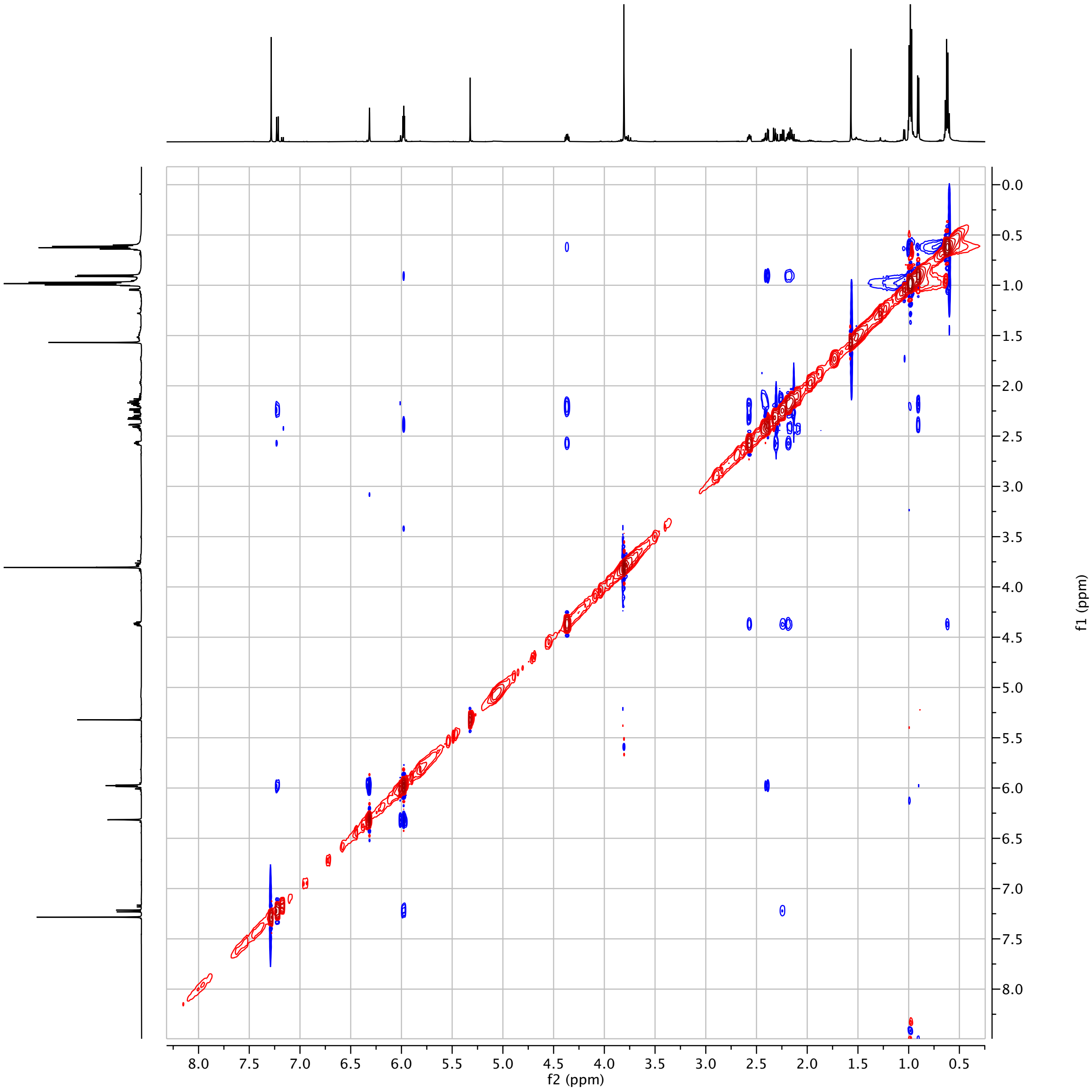

Diagnostic NOESY data for S3 derived from major oxidopyrylium cycloadduct 27 (600 MHz, $CDCl_3$)

(red – α-face; green – β-face; blue – expected and observed α,β-face correlations)

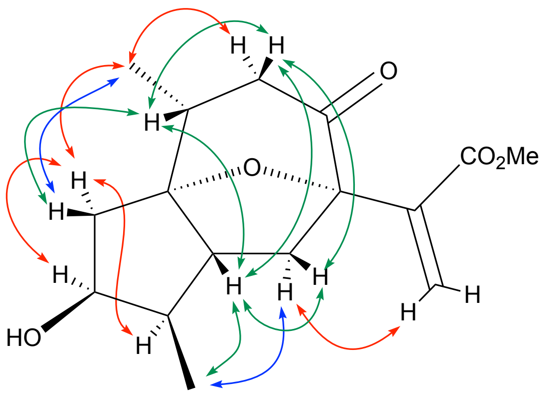

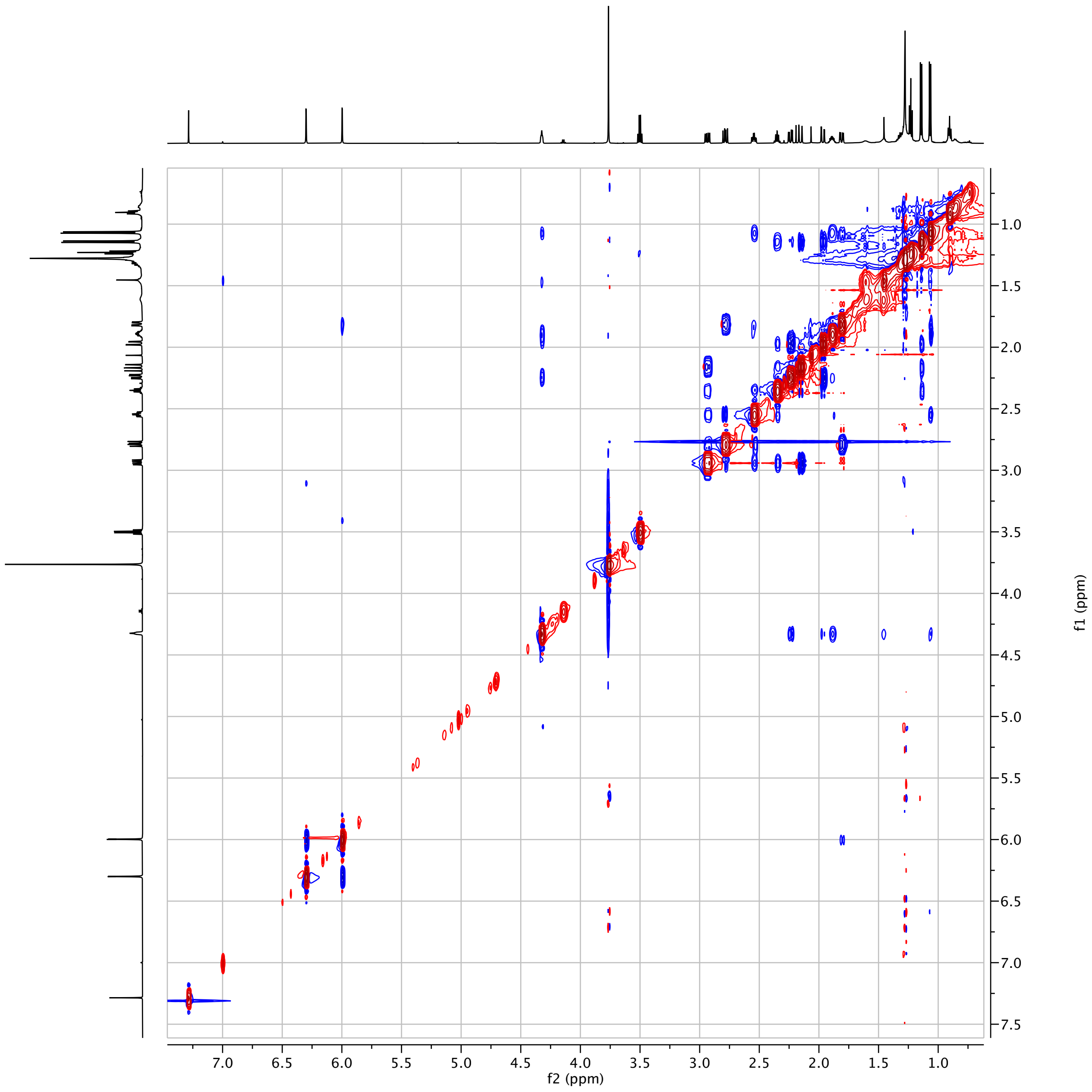

Diagnostic NOESY data for crude 36 (600 MHz, C_6D_6)

(red – α-face; green – β-face; blue – expected and observed α,β-face correlations)

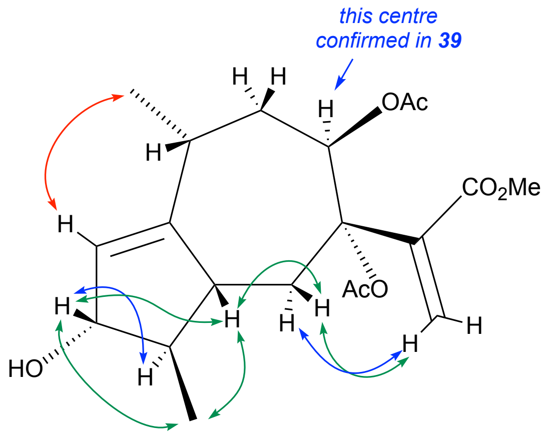

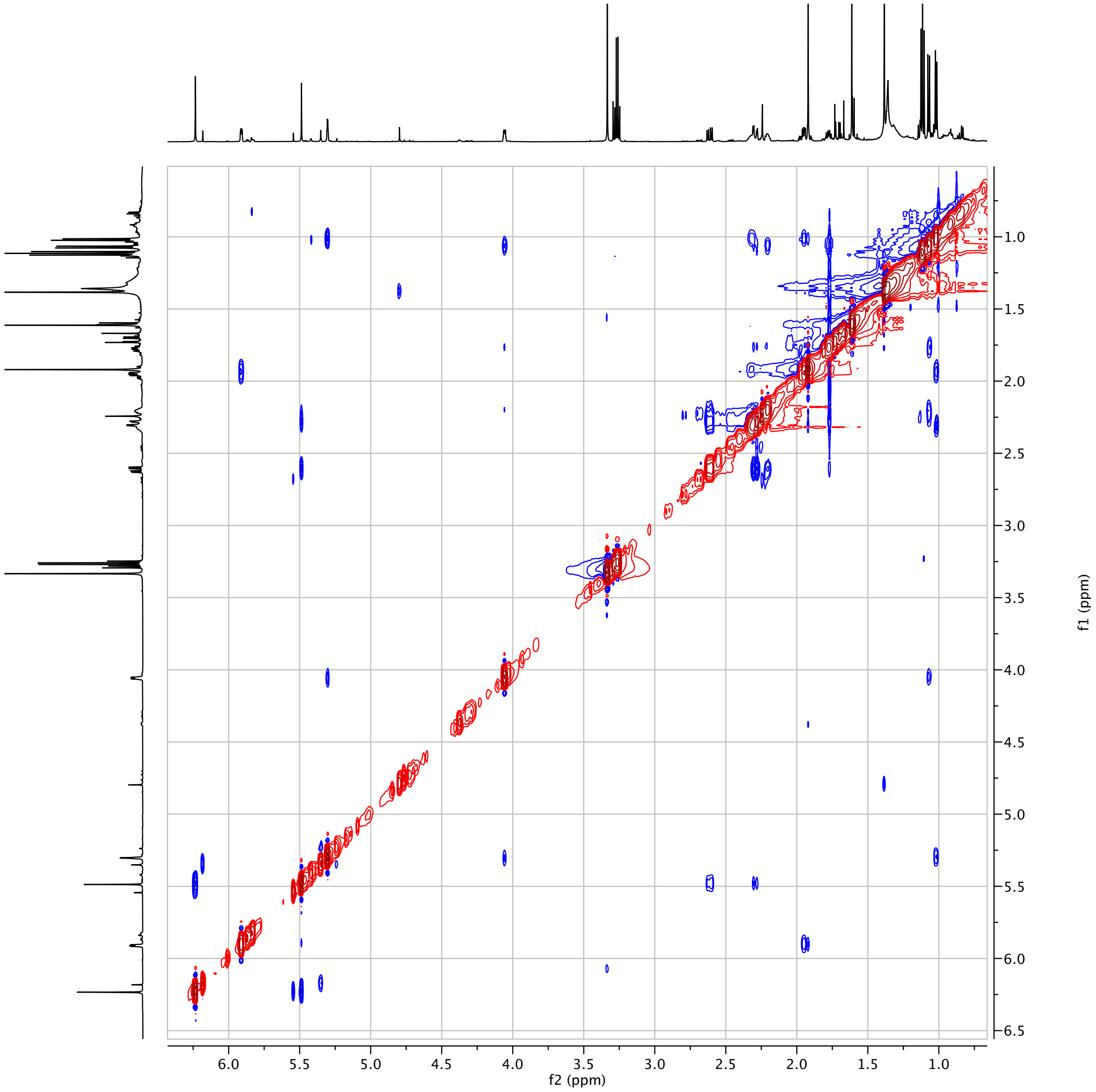

Crystallographic details for 16

Low temperature single crystal X-ray diffraction data were collected using a (Rigaku) Oxford Diffraction SuperNova diffractometer. A summary of the refinement details is contained in reference 14 of the manuscript with full refinement details provided in the CIF available from the Cambridge Crystallographic Data Centre (CCDC 2311121) via www. ccdc.cam.ac.uk/data_request/cif.

CCDC code	2311121	
Empirical formula	$C_{14}H_{22}O$	
Formula weight	206.33	
Temperature	150 K	
Wavelength	1.54184 Å	
Crystal system / Spacegroup	Orthorhombic	$P\ 2_12_12_1$
Unit cell dimensions	a = 11.65800(10) Å	α = 90°
	b = 13.89720(10) Å	β = 90°
	c = 21.91580(10) Å	γ = 90°
Volume	3550.66(4) Å^3	
Z	12	
Density (calculated)	1.158 Mg/m^3	
Crystal size	0.22 × 0.11 × 0.05 mm^3	
Independent reflections	7412 [R(int) = 0.036]	
Completeness to theta = 76.200°	99.9%	
Absorption correction	Semi-empirical from equivalents	
Refinement method	Full-matrix least-squares on F^2	
Data / restraints / parameters	7412 / 0 / 407	
Goodness-of-fit on F^2	1.0011	
Final R indices [I>2sigma(I)]	R1 = 0.0291, wR2 = 0.0773	
R indices (all data)	R1 = 0.0305, wR2 = 0.0787	
Absolute structure parameter	–0.05(4)	

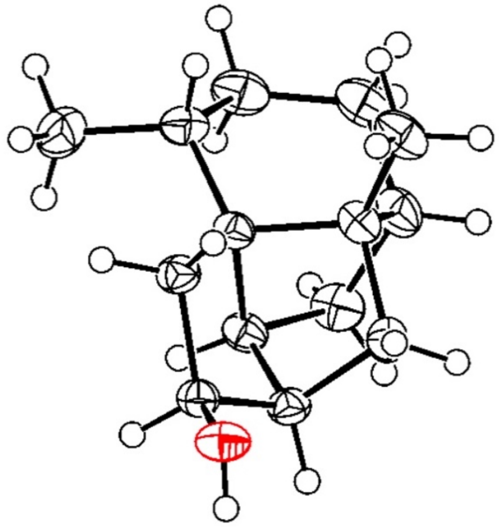

ORTEP of compound **16**; thermal ellipsoids are at the 50% probability level.
